# Global, regional, and national burden of diabetes and kidney diseases, 1990–2021: a trend and health inequality analyses based on the Global Burden of Disease Study 2021

**DOI:** 10.1080/0886022X.2025.2552956

**Published:** 2025-09-29

**Authors:** Juntao Tan, Jinglong Du, Jiaxiu Liu, Wenlong Zhao, Yanbing Liu

**Affiliations:** College of Artificial Intelligence Medicine, Chongqing Medical University, Chongqing, China

**Keywords:** Diabetes and kidney diseases, GBD, sociodemographic index, cross-country inequality

## Abstract

**Background:**

Diabetes and kidney diseases pose a major global public health challenge, impacting both health and socioeconomic development. Comprehensive analyses combining long-term trend decomposition (1990–2021) and inequality measurements are lacking.

**Methods:**

Using data from the Global Burden of Diseases, Injuries, and Risk Factors Study (GBD) 2021, we conducted comprehensive analyses to examine the disease burden through two complementary approaches (1): decomposition analysis to quantify the relative contributions of population growth, aging effects, and epidemiologic changes; and (2) inequality assessment using both the slope index of inequality and concentration index to evaluate socioeconomic disparities in disease burden across countries.

**Results:**

According to GBD 2021, the global figures for diabetes and kidney diseases in 2021 included 1,081,017,594 prevalent cases, 44,905,586 incident cases, 123,704,574 DALYs, and 3,195,034 deaths. The age-standardized rates (ASR) of estimated annual percentage change (EAPC) and average annual percentage change (AAPC) for both prevalence and incidence were positive across all countries and territories, denoting an upward trend. Population (36.92%), aging (28.64%), and epidemiologic change (i.e., changes in age-specific disease risk independent of demographic shifts, driven by diagnostics, risk factors, or treatments; 34.44%) were key drivers over 1990–2021. Significant absolute and relative inequalities in the burden of diabetes and kidney diseases, measured by sociodemographic index (SDI), were observed and showed a substantial increase over time.

**Conclusion:**

Understanding these patterns—particularly the rising burden in high-SDI nations and widening cross-country inequalities—is crucial for tailoring interventions for diabetes and kidney diseases.

## 1. Introduction

Diabetes mellitus is an endocrine disorder characterized by β-cell dysfunction or destruction, leading to relative or absolute insulin deficiency and consequent hyperglycemia [1,2]. Globally, diabetes and its complications pose an escalating public health threat. In 2021, the International Diabetes Federation (IDF) reported that 536.6 million adults were affected by diabetes, driving healthcare expenditures to $966 billion—a figure projected to exceed $1,054 billion by 2045 [3]. Notably, diabetic kidney disease (DKD), the primary microvascular complication of diabetes, affects approximately 30% of patients and represents a leading cause of chronic kidney disease worldwide [4]. Consistent with this trend, the Global Burden of Disease (GBD) 2019 study confirmed a rising disease burden: age-standardized disability-adjusted life years (DALYs) for diabetes increased from 1.1 (95% confidence interval [CI]: 1.0–1.2) in 1990 to 2.8 (95% CI: 2.5–3.1) in 2021, elevating its ranking from the 20th to the 8th leading cause of DALYs. Similarly, chronic kidney disease DALYs rose from 0.8 (95% CI: 0.8–0.9) to 1.6 (95% CI: 1.5–1.8), advancing from 29th to 18th place [5].

While previous studies report fragmented estimates, a comprehensive assessment integrating 1) multidimensional trend analysis, 2) decomposition of drivers, and 3) sociodemographic index (SDI)-based inequality metrics for diabetes and kidney diseases over 1990–2021 is lacking. This gap impedes targeted resource allocation.

To advance understanding of global diabetes and kidney disease epidemiology, we performed a comprehensive analysis using GBD 2021 data to evaluate disease burden, temporal trends, and socioeconomic disparities. Our study incorporated four key analytical approaches (1): a descriptive epidemiological assessment of prevalence, incidence, DALYs, and death across global, regional, and national levels (2); multidimensional trend analysis examining both global patterns and local variations (3); decomposition analysis quantifying contributions from population dynamics, aging, and epidemiological transitions; and (4) cross-national inequality assessment employing both the slope index of inequality (SII) and concentration index to measure socioeconomic gradients in disease burden.

## 2. Methods

### 2.1. Data collection and case definition

To quantify global health challenges, the GBD study—a multinational collaborative database—systematically integrates diverse data sources [6–9]. These include population censuses, disease registries, household surveys, civil registrations, healthcare utilization records, environmental monitoring (e.g., air pollution, satellite imaging), and epidemiological notifications [10,11]. By synthesizing these inputs, GBD generates consistent, transparent estimates of death and disability burdens for diseases and injuries worldwide. Critically, it provides standardized metrics (e.g., prevalence, incidence, DALYs) to evaluate health loss, enabling tailored interventions aligned with the WHO’s Sustainable Development Goals [12].

We obtained data from the 2021 Global Burden of Disease (GBD) study through the Global Health Data Exchange (GHDx) query tool (https://vizhub.healthdata.org/gbd-results/), which provides comprehensive estimates for 369 diseases and injuries and 88 risk factors across 204 countries and territories. In this study, rates, numbers, and 95% uncertainty intervals (UIs) of prevalence, incidence, DALYs, and deaths related to diabetes and kidney diseases were reported. All rates (including incidence rates) are expressed per 100,000 person-years, using mid-year population estimates as denominators [13]. Additionally, we incorporated the SDI as a key metric, which integrates three fundamental development indicators—total fertility rate among women under 25 years, mean educational attainment in individuals aged 15 years or older, and lag-distributed per capita income—to comprehensively assess the socioeconomic determinants of health status across different regions [14].

It is important to note that the GBD 2021 estimates for diabetes and kidney diseases are derived from modeled epidemiological data and do not involve primary collection or central review of biopsy specimens for case definition or scoring. Case definitions within the GBD framework are based on clinical diagnosis, laboratory criteria (e.g., HbA1c, eGFR, albuminuria), and relevant ICD codes, standardized across input data sources. The 1990–2021 timeframe was selected to capture three decades of global epidemiological transition, encompassing key shifts in population aging, SDI trajectories, and disease management paradigms.

### 2.2. Descriptive analysis

We conducted a comprehensive descriptive analysis of diabetes and kidney disease burden across multiple geographical scales and demographic dimensions, comparing ASRs of prevalence, incidence, DALYs, and deaths between 1990 and 2019. This analysis encompassed sex-specific differences, 21 GBD regions, 204 countries and territories, and all five SDI quintiles to provide a complete epidemiological profile of these conditions.

### 2.3. Trend analysis

The ASR and estimated annual percentage change (EAPC) were used to assess trends in the prevalence, incidence, DALYs, and deaths of diabetes and kidney diseases. A significantly increasing trend in ASR was identified when both the EAPC value and the lower boundary of its 95% CI exceeded zero, while a decreasing trend was confirmed when both the EAPC value and the upper boundary of its 95% CI were below zero. Furthermore, we applied the joinpoint regression model (JRM) to examine the local trends in the disease burden from 1990 to 2021, calculating the average annual percent change (AAPC) as a weighted average of the slope coefficients between joinpoints, with weights corresponding to the duration of each segment [15]. The AAPC was then converted into a percentage change for interpretation. Statistically significant upward trends were determined when both the AAPC value and the lower bound of its 95% CI were greater than zero, whereas downward trends were established when both the AAPC value and the upper bound of its 95% CI were less than zero. EAPC assumes a linear trend over the entire period, while AAPC takes into account joinpoints, providing a more nuanced trend analysis by averaging the slopes between joinpoints.

### 2.4. Decomposition analysis

To comprehensively assess the factors driving changes in the prevalence, incidence, DALYs, and deaths of diabetes and kidney diseases between 1990 and 2021, we performed a sex-stratified decomposition analysis (for both males and females) that accounted for population growth, aging effects, and epidemiologic changes. This approach allowed us to quantify the relative contributions of demographic shifts and disease-specific risk factors to the observed trends in disease burden over the study period. In decomposition analysis, ‘population’ refers to growth in total population size; ‘aging’ denotes shifts in age distribution toward older groups; and ‘epidemiologic change’ captures alterations in age-specific disease risk due to factors beyond demography (e.g., diagnostics, risk factors, treatments).

### 2.5. Cross-country inequality analysis

To evaluate socioeconomic inequalities in the burden of diabetes and kidney diseases across countries, we applied two standardized measures—the SII and the concentration index—following WHO methodological guidelines, with disparities quantified based on the SDI [16]. The SII measures absolute inequality by estimating the difference between the highest and lowest values of a health indicator across a socioeconomic gradient. The concentration index measures relative inequality by summarizing the extent to which a health indicator is concentrated among a particular socioeconomic group. The concentration index (relative inequality) ranges from −1 to 1; positive values indicate higher burden among high-SDI populations. These indices describe the magnitude of inequality but do not adjust for other potential confounding variables beyond the socio-economic dimension represented by SDI.

### 2.6. Statistical analysis

All statistical analyses were conducted using Stata (version 15.0), the Joinpoint Regression program (version 5.2.0), and R (version 4.3.3).

## 3. Results

### 3.1. Descriptive analysis of diabetes and kidney disease burden

The global burden of diabetes and kidney diseases increased substantially from 1990 to 2021. The number of prevalent cases rose from 455,036,849 (95% UI: 397,609,741–520,001,233) to 1,081,017,594 (95% UI: 971,891,393–1,201,444,768; +137.57%; Table 1). The number of incident cases increased from 16,198,875 (95% UI: 12,728,191–19,998,124) to 44,905,586 (95% UI: 36,221,360–54,123,171; +177.21%; Supplementary Table 1). DALYs grew from 48,811,345 (95% UI: 43,209,260–54,928,854) to 123,704,574 (95% UI: 107,440,163–143,210,093; +153.43%; Supplementary Table 2), while death counts increased from 1,237,570 (95% UI: 1,145,414–1,329,536) to 3,195,034 (95% UI: 2,887,971–3,428,787; +158.17%; Supplementary Table 3). Regionally, South Asia reported the highest absolute numbers for prevalence, incidence, DALYs, and deaths. Oceania exhibited the highest ASR for prevalence, DALYs, and mortality rate, while North Africa and the Middle East showed the highest ASR for incidence (Table 1; Supplementary Tables 1-3). Nationally, China had the highest number of prevalence and incidence cases, whereas India recorded the highest DALYs and deaths. The Marshall Islands showed the highest ASR for prevalence, Qatar for incidence, and Fiji for both DALYs and mortality rate (ASR) (Supplementary Tables 4-7). Furthermore, within SDI quintiles in 2021, the middle SDI quintile had the highest absolute case numbers. Mortality rate (ASR) decreased with increasing SDI, while prevalence ASR peaked in the low-middle SDI quintile. The high SDI quintile exhibited the highest incidence ASR, and low SDI quintile showed the highest ASR for both DALYs and mortality rate.

**Table 1.** The case number and ASR of prevalence of diabetes and kidney diseases in 1990 and 2021, and its temporal trends from 1990 to 2021.

location	1990		2021		EAPC (95% CI)	AAPC (95% CI)	P value
	Case number (95% UI)	ASR (95% UI)	Case number (95% UI)	ASR (95% UI)			
**Global**	455,036,849	10374.06	1,081,017,594	12739.27	0.67%	0.66%	<0.001
	(397,609,741 to 520,001,233)	(9139.00 to 11758.79)	(971,891,393 to 1,201,444,768)	(11438.19 to 14176.99)	(0.64 to 0.70)	(0.63 to 0.70)	
**Sex**							
Male	216,883,709	10259.31	528,996,635	12896.16	0.75%	0.74%	<0.001
	(189,054,069 to 248,294,911)	(9041.10 to 11617.15)	(476,281,505 to 587,663,665)	(11616.42 to 14318.47)	(0.72 to 0.77)	(0.71 to 0.77)	
Female	238,153,140	10462.19	552,020,959	12579.91	0.60%	0.60%	<0.001
	(208,389,093 to 271,844,167)	(9204.98 to 11876.89)	(495,832,764 to 614,079,619)	(11264.19 to 14037.88)	(0.57 to 0.63)	(0.57 to 0.62)	
**SDI**							
High SDI	96,790,965	9218.11	210,163,996	12262.06	0.93%	0.93%	<0.001
	(86,409,683 to 108,383,017)	(8199.42 to 10362.22)	(194,566,468 to 226,883,089)	(11219.78 to 13393.81)	(0.88 to 0.98)	(0.91 to 0.94)	
High-middle SDI	102,691,407	9933.39	209,673,765	11897.98	0.62%	0.59%	<0.001
	(89,788,720 to 117,100,367)	(8721.76 to 11280.64)	(188,808,670 to 232,702,062)	(10635.07 to 13302.95)	(0.59 to 0.65)	(0.56 to 0.62)	
Middle SDI	139,334,629	10985.85	350,776,375	12922.22	0.54%	0.53%	<0.001
	(121,166,025 to 160,046,276)	(9684.31 to 12448.16)	(313,952,436 to 391,739,569)	(11559.71 to 14436.39)	(0.51 to 0.57)	(0.51 to 0.56)	
Low-middle SDI	86,821,511	11305.04	228,201,964	13784.13	0.61%	0.65%	<0.001
	(74,520,610 to 100,845,367)	(9850.36 to 12937.15)	(202,026,483 to 257,335,850)	(12301.49 to 15422.07)	(0.58 to 0.65)	(0.61 to 0.69)	
Low SDI	28,953,998	9938.46	81,288,241	11736.5	0.50%	0.54%	<0.001
	(24,930,405 to 33,506,625)	(8696.04 to 11321.35)	(71,383,040 to 92,603,355)	(10475.28 to 13161.39)	(0.48 to 0.52)	(0.52 to 0.56)	
**Region**							
Andean Latin America	1,923,183	7670.84	6,266,439	10013.75	0.91%	0.86%	<0.001
	(1,669,489 to 2,211,691)	(6750.18 to 8703.54)	(5,602,381 to 6,981,286)	(8989.29 to 11108.93)	(0.88 to 0.93)	(0.85 to 0.87)	
Australasia	1,754,210	7654.12	4,143,219	8945.9	0.55%	0.51%	<0.001
	(1,556,112 to 1,971,473)	(6775.81 to 8623.40)	(3,758,086 to 4,551,284)	(8017.18 to 9945.58)	(0.52 to 0.57)	(0.48 to 0.55)	
Caribbean	2,990,639	10453.88	7,318,826	13933.12	0.92%	0.93%	<0.001
	(2,666,190 to 3,342,410)	(9385.10 to 11603.14)	(6,668,563 to 7,993,854)	(12675.09 to 15241.17)	(0.91 to 0.92)	(0.92 to 0.94)	
Central Asia	6,246,096	11880.79	12,787,250	14204.42	0.61%	0.58%	<0.001
	(5,499,332 to 7,102,845)	(10551.38 to 13384.56)	(11,519,215 to 14,185,161)	(12852.09 to 15681.67)	(0.60 to 0.62)	(0.57 to 0.59)	
Central Europe	12,617,398	8826.42	19,368,488	10301.9	0.46%	0.49%	<0.001
	(11,323,270 to 14,038,790)	(7895.25 to 9849.64)	(17,782,604 to 21,061,377)	(9328.10 to 11358.95)	(0.45 to 0.48)	(0.47 to 0.50)	
Central Latin America	13,162,874	12702.98	38,002,224	14649.74	0.42%	0.47%	<0.001
	(11,768,073 to 14,711,235)	(11509.91 to 14016.14)	(34,819,499 to 41,531,878)	(13441.24 to 15987.29)	(0.41 to 0.44)	(0.45 to 0.48)	
Central Sub-Saharan Africa	3,203,888	10905.75	10,032,934	12879.12	0.50%	0.54%	<0.001
	(2,788,050 to 3,676,554)	(9669.82 to 12270.08)	(8,934,630 to 11,271,057)	(11682.88 to 14210.90)	(0.48 to 0.51)	(0.53 to 0.55)	
East Asia	99,538,918	9829.15	224,926,234	11462.46	0.58%	0.51%	<0.001
	(86,907,715 to 113,496,880)	(8670.98 to 11099.34)	(202,103,578 to 249,674,155)	(10245.30 to 12790.56)	(0.50 to 0.65)	(0.46 to 0.56)	
Eastern Europe	27,495,175	10539.15	35,740,440	11978.07	0.41%	0.41%	<0.001
	(23,556,777 to 31,883,446)	(9000.96 to 12258.13)	(31,490,016 to 40,437,932)	(10409.25 to 13723.55)	(0.40 to 0.42)	(0.41 to 0.42)	
Eastern Sub-Saharan Africa	7,492,653	7133.78	20,843,467	8231.28	0.46%	0.46%	<0.001
	(6,308,565 to 8,864,693)	(6117.49 to 8295.15)	(17,755,682 to 24,412,865)	(7159.44 to 9453.86)	(0.45 to 0.48)	(0.45 to 0.47)	
High-income Asia Pacific	22,250,423	11173.44	45,678,495	13478.08	0.51%	0.60%	<0.001
	(19,652,316 to 25,159,915)	(9859.46 to 12647.23)	(41,949,144 to 49,612,719)	(12167.98 to 14885.92)	(0.45 to 0.57)	(0.55 to 0.66)	
High-income North America	33,243,998	9983.87	78,654,724	14025.1	1.22%	1.10%	<0.001
	(29,686,676 to 37,222,188)	(8885.25 to 11220.16)	(73,324,261 to 84,604,734)	(12935.88 to 15255.28)	(1.18 to 1.26)	(1.03 to 1.17)	
North Africa and Middle East	24,874,148	11570.65	87,763,674	16117.6	1.08%	1.07%	<0.001
	(21,449,549 to 28,836,978)	(10142.17 to 13196.20)	(78,671,420 to 97,680,344)	(14578.41 to 17784.00)	(1.04 to 1.12)	(1.06 to 1.09)	
Oceania	499,248	12304.57	1,812,712	17878.29	1.20%	1.21%	<0.001
	(439,575 to 565,559)	(11008.37 to 13733.34)	(1,636,968 to 1,997,959)	(16333.45 to 19486.67)	(1.18 to 1.22)	(1.19 to 1.22)	
South Asia	89,191,951	12008.36	238,299,004	14097.17	0.46%	0.53%	<0.001
	(76,355,891 to 103,847,396)	(10437.81 to 13771.85)	(209,583,449 to 270,303,846)	(12481.79 to 15885.03)	(0.42 to 0.51)	(0.45 to 0.62)	
Southeast Asia	39,228,527	11937.58	98,811,421	14018.32	0.47%	0.53%	<0.001
	(32,972,317 to 46,368,984)	(10216.37 to 13870.55)	(86,027,704 to 113,144,032)	(12280.40 to 15959.13)	(0.44 to 0.51)	(0.51 to 0.55)	
Southern Latin America	3,761,373	8035.1	8,830,310	10768.94	0.99%	0.95%	<0.001
	(3,305,198 to 4,275,325)	(7071.27 to 9119.20)	(7,947,575 to 9,774,013)	(9646.48 to 11975.97)	(0.97 to 1.01)	(0.92 to 0.97)	
Southern Sub-Saharan Africa	3,576,276	10732.63	8,589,550	12892.15	0.61%	0.59%	<0.001
	(3,117,627 to 4,099,996)	(9504.09 to 12116.82)	(7,644,530 to 9,630,362)	(11600.04 to 14308.48)	(0.60 to 0.62)	(0.59 to 0.60)	
Tropical Latin America	11,600,661	10669.32	30,020,689	11663.57	0.31%	0.31%	<0.001
	(10,169,100 to 13,227,390)	(9479.16 to 12012.31)	(26,875,767 to 33,518,992)	(10436.93 to 13028.50)	(0.29 to 0.34)	(0.27 to 0.36)	
Western Europe	39,466,600	7568.48	70,819,215	9701.22	0.76%	0.80%	<0.001
	(35,626,737 to 43,711,361)	(6779.34 to 8450.41)	(65,297,990 to 76,637,906)	(8816.40 to 10643.70)	(0.71 to 0.80)	(0.76 to 0.85)	
Western Sub-Saharan Africa	10,918,612	9909.73	32,308,280	11736.89	0.59%	0.55%	<0.001
	(9,539,007 to 12,483,539)	(8788.29 to 11160.97)	(28,510,507 to 36,548,137)	(10554.01 to 13044.53)	(0.57 to 0.60)	(0.53 to 0.57)	

ASR: age-standardized rate; EAPC: estimated annual percentage change; AAPC: average annual percent change; SDI: sociodemographic index; UI: uncertainty interval; CI: confidence interval.

### 3.2. Trend analysis

**Figure 1.**
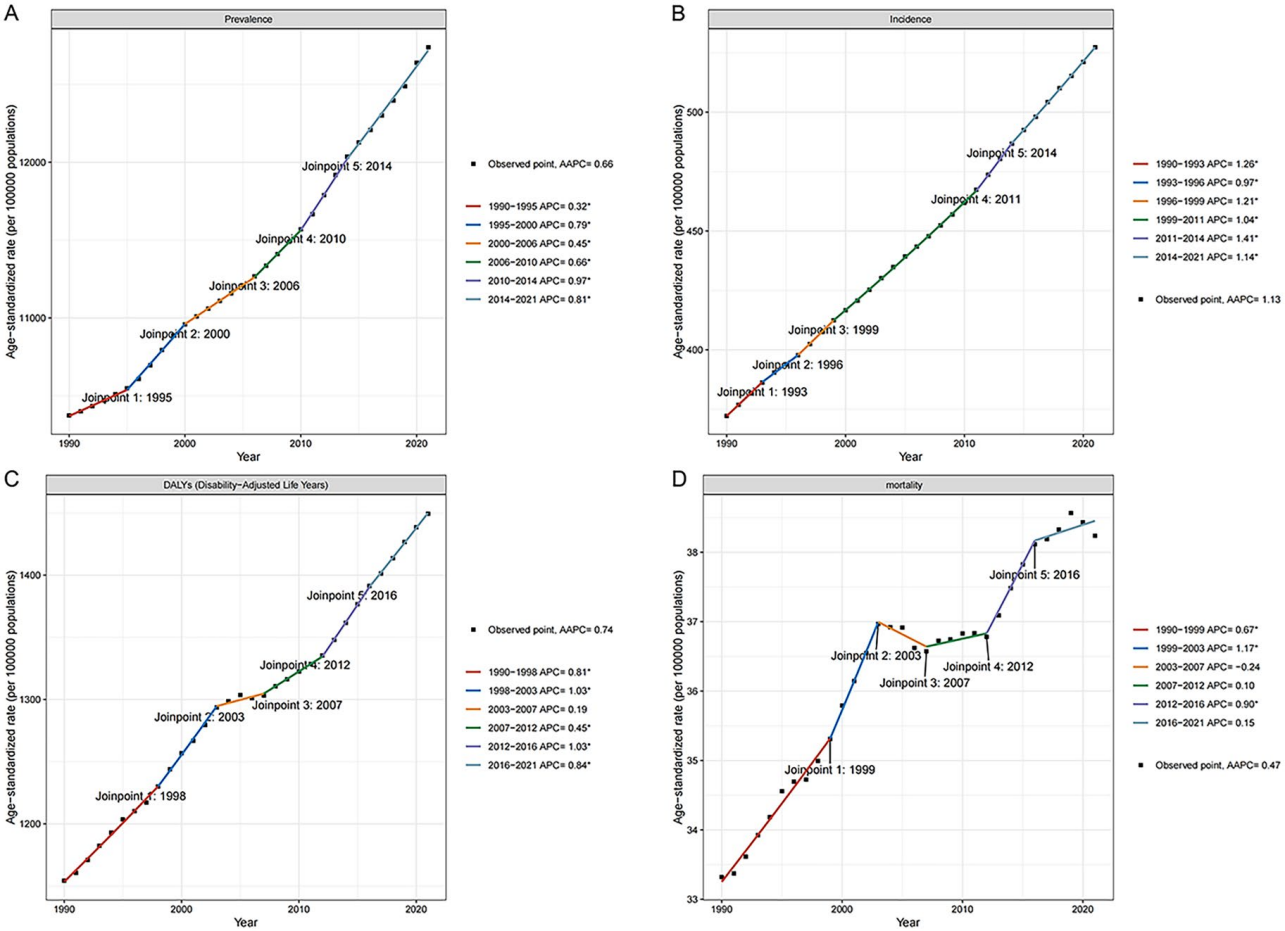
(A) The joinpoint regression analysis on the ASR of prevalence. (B) The joinpoint regression analysis on the ASR of incidence. (C) The joinpoint regression analysis on the ASR of DALYs. (D) The joinpoint regression analysis on mortality rate (ASR). ASR: age-standardized rate; DALYs: disability-adjusted life years.

Globally, ASR demonstrated an overall upward trend, though local trends varied across joinpoints (Figure 1). Joinpoint analysis revealed sustained increases in ASR for prevalence and incidence throughout 1990–2021. For DALYs, five periods of increase (1990–1998, 1998–2003, 2007–2012, 2012–2016, 2016–2021) were observed with stable trends during 2003–2007. Mortality rate (ASR) increased during three periods (1990–1999, 1999–2003, 2012–2016) and remained stable during 2003–2007, 2007–2012, and 2016–2021.

Analysis of regions with the highest AAPC revealed distinct patterns: For prevalence (Supplementary Figure 1), Oceania showed the most rapid increase (APC = 1.55, 1990–1994), followed by high-income North America (APC = 1.70, 1996–2000), North Africa and Middle East (APC = 1.54, 2011–2014), and Southern Latin America (APC = 1.16, 1995–2005). Incidence trends in North Africa and Middle East, Andean Latin America, Central Asia, and Eastern Europe declined toward the end of their observation periods (Supplementary Figure 2). Central Asia and Southern Sub-Saharan Africa demonstrated decreasing DALY trends during 2016–2021 and 2015–2021 respectively, while high-income North America and Eastern Europe showed increases (Supplementary Figure 3). Eastern Europe experienced a marked mortality rate (ASR) surge (APC = 20.01, 2012–2016; Supplementary Figure 4). Country-level results are shown in Supplementary Figures 5-8.

**Figure 2.**
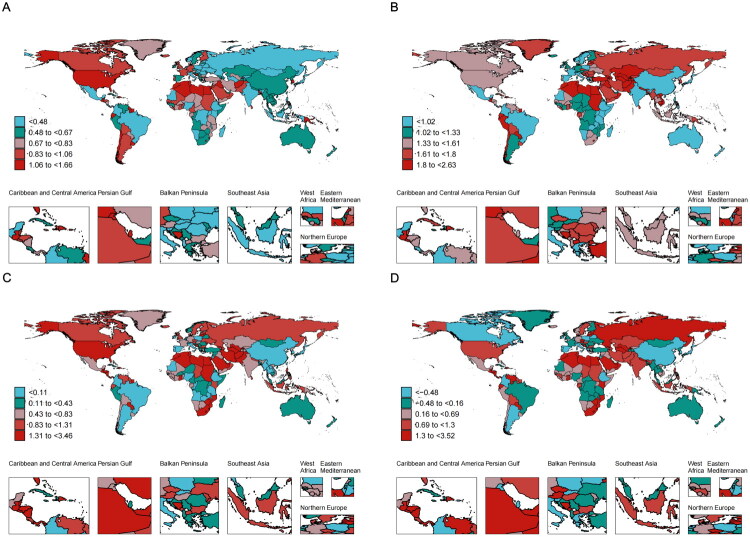
(A) The EAPC of ASR for prevalence from 1990 to 2021. (B) The EAPC of ASR for incidence from 1990 to 2021. (C) The EAPC of ASR for DALYs from 1990 to 2021. (D) The EAPC of mortality rate (ASR) from 1990 to 2021. ASR: age-standardized rate; EAPC: estimated annual percentage change; DALYs: disability-adjusted life years.

**Figure 3.**
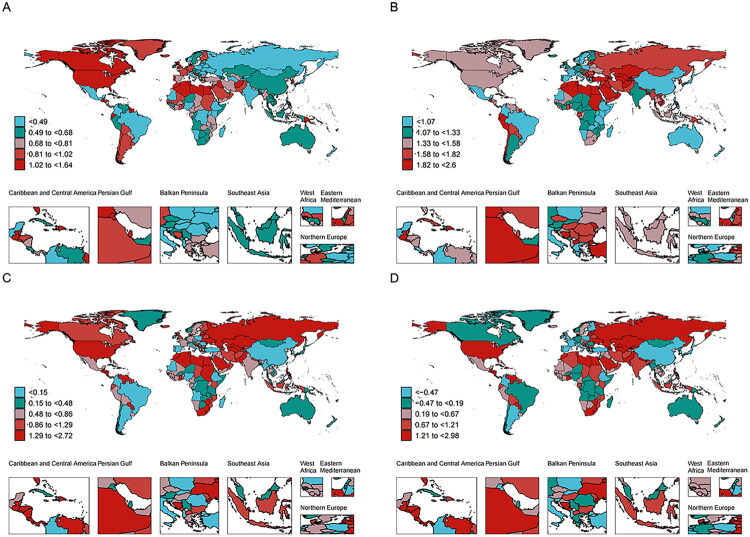
(A) The ASR of AAPC of prevalence from 1990 to 2021. (B) The ASR of AAPC of incidence from 1990 to 2021. (C) The ASR of AAPC of DALYs from 1990 to 2021. (D) The AAPC of mortality rate (ASR) from 1990 to 2021. ASR: age-standardized rate; AAPC: average annual percentage change; DALYs: disability-adjusted life years.

The variability in the estimates was assessed using 95% CIs [Table 1, Supplementary Tables 1-3]. The magnitude of the positive growth trends, as indicated by EAPC and AAPC values, was statistically significant with narrow CIs (e.g., global prevalence EAPC: 0.67%, 95% CI 0.64–0.70), suggesting reliable and meaningful health impacts. Country-level results are shown in Supplementary Tables 4-7. Globally, EAPCs for ASR were: prevalence 0.67% (95% CI: 0.64–0.70), incidence 1.12% (95% CI: 1.10–1.13), DALYs 0.69% (95% CI: 0.66–0.72), and mortality rate 0.45% (95% CI: 0.40–0.50; Table 1; Supplementary Tables 1-3). Corresponding AAPCs were: prevalence 0.66% (95% CI: 0.63–0.70), incidence 1.13% (95% CI: 1.09–1.17), DALYs 0.74% (95% CI: 0.69–0.79), and mortality rate 0.47% (95% CI: 0.37–0.57; Table 1**;** Supplementary Tables 1-3). Nationally, all 204 countries/territories showed positive growth trends (ASR EAPC/AAPC >0) for prevalence and incidence. The Marshall Islands had the highest EAPC for prevalence (1.66%; 95% CI: 1.60–1.72) and Morocco for incidence (2.63%; 95% CI: 2.58–2.68), while Lesotho showed the highest EAPCs for DALYs (3.46%; 95% CI: 3.00–3.93) and mortality rate (3.52%; 95% CI: 3.00–4.03; Supplementary Tables 4-7; Figures 2 and 3).

### 3.3. Decomposition analysis

**Figure 4.**
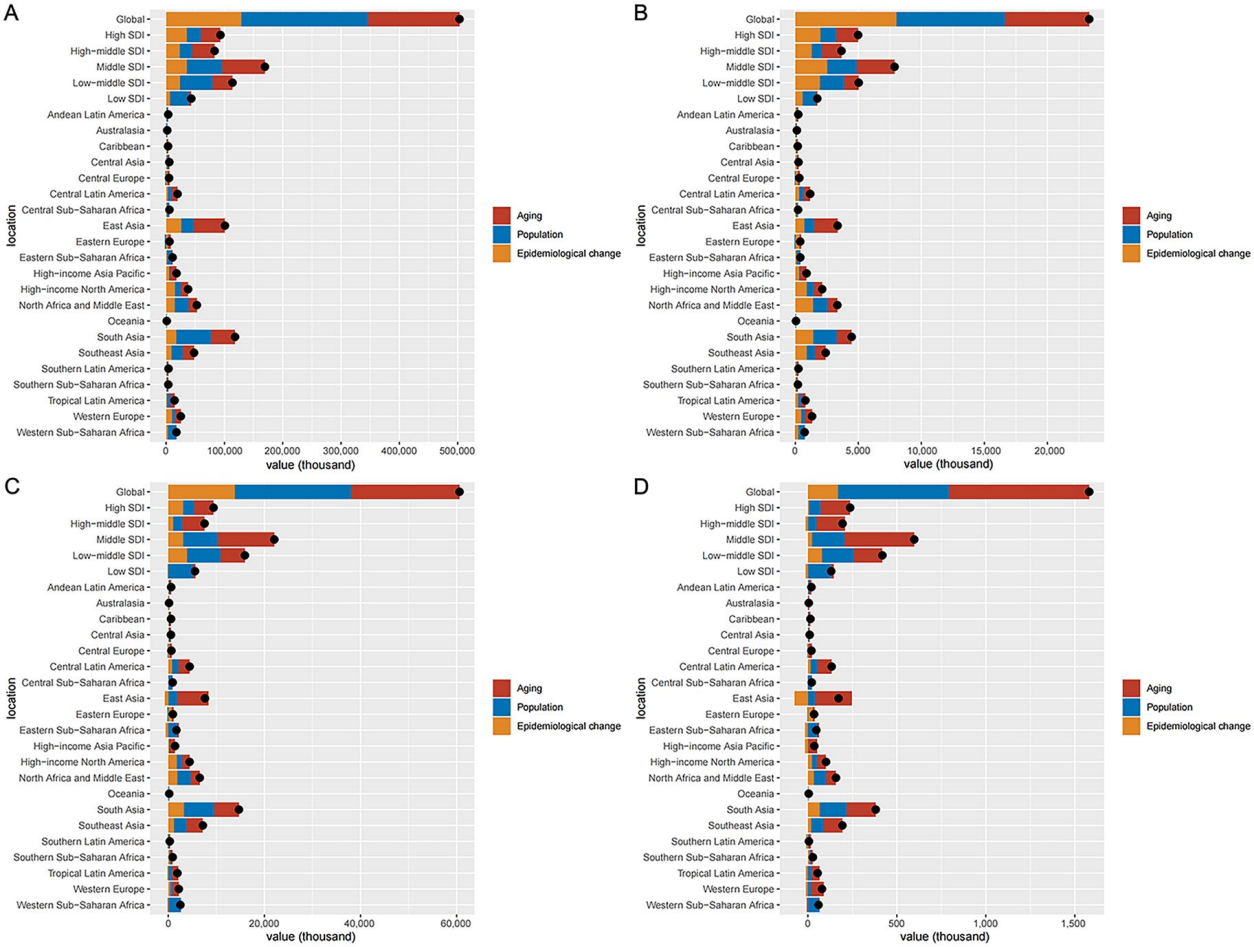
(A) Changes prevalence of diabetes and kidney diseases according to population, aging and epidemiologic change from 1990 to 2021. (B) Changes incidence of diabetes and kidney diseases according to population, aging and epidemiologic change from 1990 to 2021. (C) Changes DALYs of diabetes and kidney diseases according to population, aging and epidemiologic change from 1990 to 2021. (D) Changes death of diabetes and kidney diseases according to population, aging and epidemiologic change from 1990 to 2021. The x-axis values are presented in thousands for clarity. DALYs: disability-adjusted life years.

We conducted a decomposition analysis on the number of cases to explore the impact of population growth, aging, and epidemiologic change on the prevalence, incidence, DALYs, and deaths of diabetes and kidney diseases from 1990 to 2021. Globally, the prevalence of diabetes and kidney diseases increased by 43.04%, 31.32%, and 25.64%, respectively, between 1990 and 2021, resulting in population growth, aging, and epidemiologic change (Figure 4 and Supplementary Table 8). Population, aging, and epidemiologic change accounted for 36.92%, 28.64%, and 34.44% of the global increase in the incidence of diabetes and kidney diseases, respectively, between 1990 and 2021 (Figure 4 and Supplementary Table 9). The global increase in DALYs and deaths of diabetes and kidney diseases were most pronounced in the context of population and aging, with rates of 40.03% and 49.79%, respectively (Figure 4, Supplementary Tables 10-11). The most pronounced contributions of population, aging, and epidemiologic change to the rise in prevalence of diabetes and kidney diseases occurred in the low SDI quintile (74.41%), high-middle SDI quintile (46.48%), and high SDI quintile (37.82%), respectively. The effects of population, aging, and epidemiologic change on the prevalence, incidence, DALYs, and deaths of diabetes and kidney diseases varied across subgroups when stratified by sexes (Supplementary Figure 9). A list of countries included in each region is provided in Supplementary Table 12.

### 3.4. Cross-country inequality analysis

**Figure 5.**
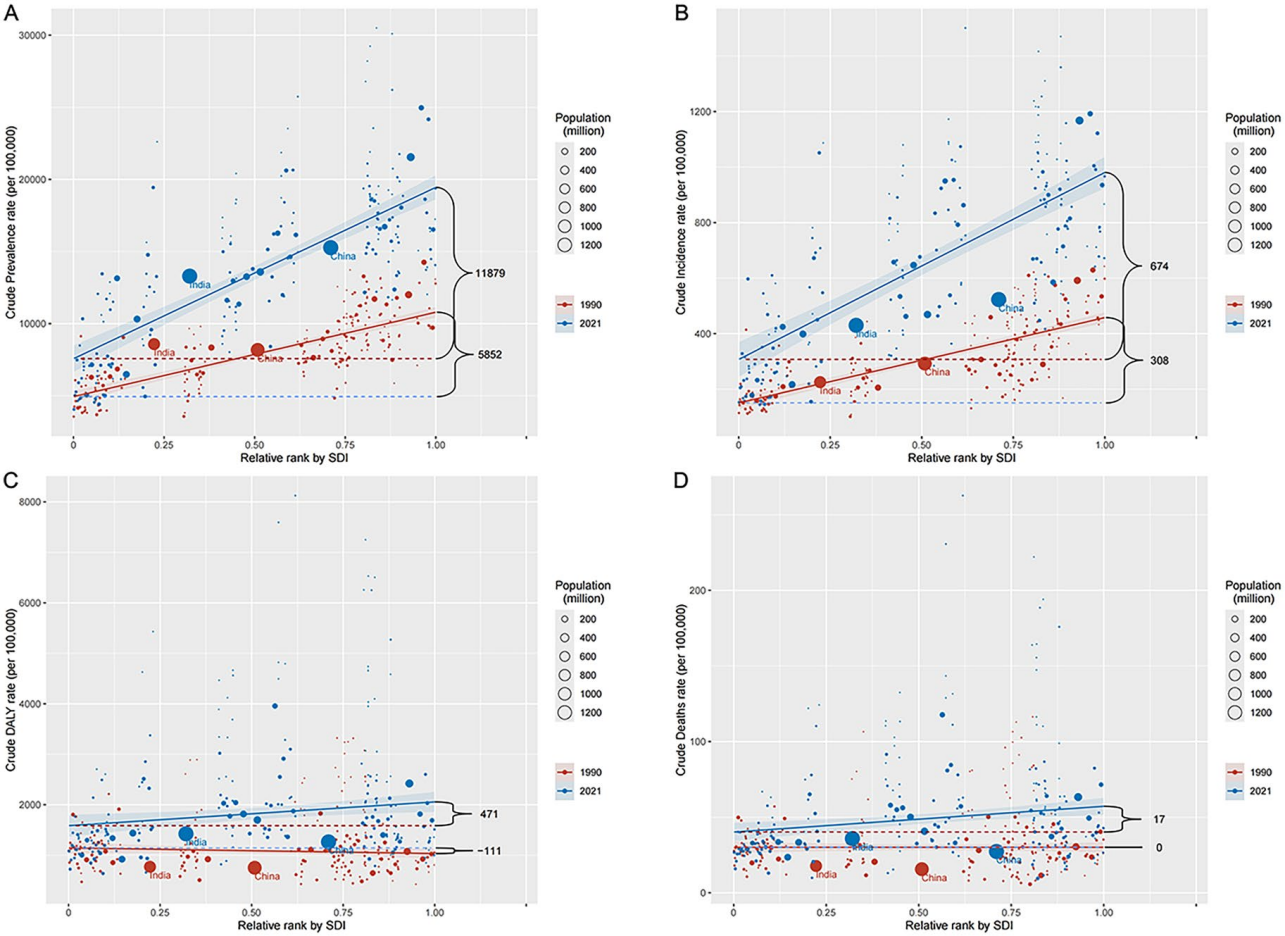
(A) SDI-related health inequality regression curve for the prevalence of diabetes and kidney diseases worldwide, 1990 and 2021. (B) SDI-related health inequality regression curve for the incidence of diabetes and kidney diseases worldwide, 1990 and 2021. (C) SDI-related health inequality regression curve for the DALYs of diabetes and kidney diseases worldwide, 1990 and 2021. (D) SDI-related health inequality regression curve for the death of diabetes and kidney diseases worldwide, 1990 and 2021. DALYs: disability-adjusted life years; SDI: sociodemographic index.

**Figure 6.**
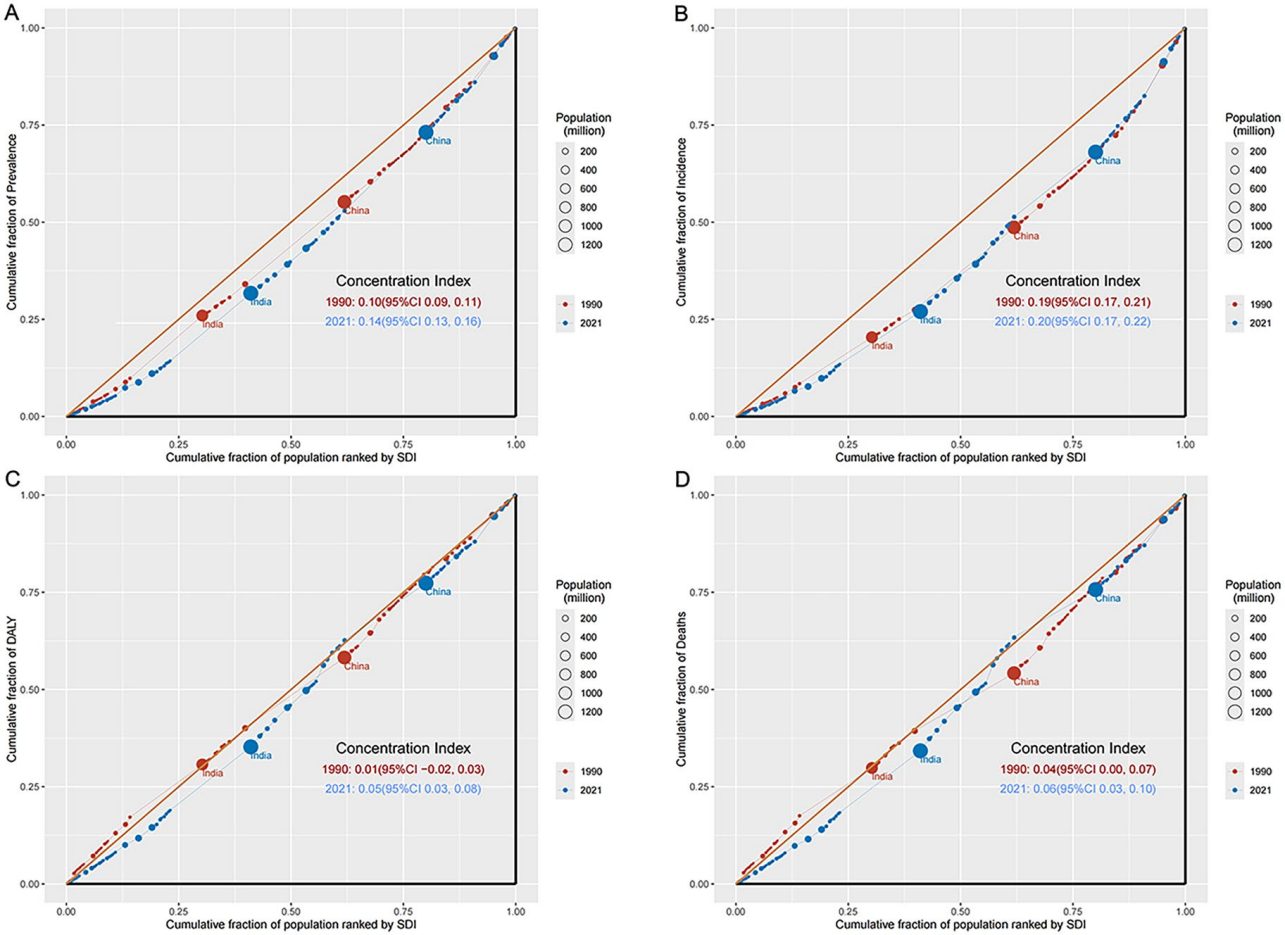
(A) SDI-related concentration curve for the prevalence of diabetes and kidney diseases worldwide, 1990 and 2021. (B) SDI-related concentration curve for the incidence of diabetes and kidney diseases worldwide, 1990 and 2021. (C) SDI-related concentration curve for the DALYs of diabetes and kidney diseases worldwide, 1990 and 2021. (D) SDI-related concentration curve for the death of diabetes and kidney diseases worldwide, 1990 and 2021. DALYs: disability-adjusted life years; SDI: sociodemographic index.

Significant absolute and relative inequalities in the burden of diabetes and kidney diseases, measured by SDI, were observed and showed a substantial increase over time (Figures 5 and 6). The data presented in Figures 5(A) and 6(A) reveal a striking divergence in prevalence rates between highest- and lowest-SDI countries, with the SII increasing from 5,852.11 (95% CI: 5,172.02–6,532.19) in 1990 to 11,878.59 (95% CI: 10,363.97–13,393.20) in 2021, accompanied by a rise in the concentration index from 0.10 (95% CI: 0.09–0.11) to 0.14 (95% CI: 0.13–0.16) over the same period, demonstrating a progressively unequal distribution of disease burden across the SDI spectrum. Notably, while socioeconomic disparities widened between nations, the epidemiological profiles of diabetes and kidney diseases showed increasing convergence within high-SDI countries, with prevalence, incidence, DALYs, and mortality rates exhibiting similar patterns across these affluent nations.

## 4. Discussion

Focusing on the absolute number of prevalent and incident cases provides a comprehensive view of the overall disease burden, which is crucial for resource allocation and public health planning. Absolute counts highlight the sheer volume of individuals affected, which is essential for understanding the scale of the problem and planning healthcare services. While proportions and rates are important for comparing disease burden across different populations, absolute numbers offer a direct measure of the total impact on the healthcare system and society. This approach is particularly relevant for diseases like diabetes and kidney diseases, which have significant implications for both individual health and healthcare infrastructure.

This study offers the most current global, regional, and national estimates of diabetes and kidney disease burden from 1990 to 2021, encompassing prevalence, incidence, DALYs, and deaths metrics. Through comprehensive trend, decomposition, and cross-country inequality analyses, we identified a consistent global increase in disease burden despite significant inter-country variations. High-SDI nations carried a disproportionate share of this burden, with socioeconomic disparities widening progressively over time. Our decomposition analysis revealed distinct regional patterns in kidney disease burden attributable to demographic aging, population growth, and epidemiological transitions. These findings underscore the growing public health challenge posed by diabetes and kidney diseases, highlighting the need for targeted interventions tailored to specific national contexts.

Diabetes and kidney diseases exhibited marked heterogeneity in global burden distribution in 2021, with South Asia bearing the highest absolute cases while Oceania and North Africa/Middle East showed the most elevated age-standardized rates. This geographic disparity underscores the necessity for tailored health policies. It is important to note that while the Marshall Islands, American Samoa, and Morocco may not have the highest absolute numbers of diabetes and kidney disease cases due to their smaller populations, they still face significant health challenges. The ASR of prevalence, incidence, DALYs, and mortality rate in these countries have shown substantial increases from 1990 to 2021, indicating a growing burden that requires attention. Similar to previous studies [17–20], our findings confirm accelerating global burden trends. This increase is attributed to the fact that diabetes and kidney diseases incur not only direct (drug expenses and outpatient care) and indirect (absenteeism, loss of family productivity, premature death) costs, as well as intangible (decreased quality of life, pain) personal costs [21–23], the estimated medical cost of diabetes and kidney diseases accounts for 5.1% of the total expenditure, which may be underestimated [24]. These trends suggest that additional resources and targeted interventions should be allocated to prevent and manage diabetes and kidney diseases in these regions to address the emerging health crisis effectively.

Quantifying cross-country inequalities in disease burden enables precise identification of diabetes and kidney disease distribution patterns and pinpoints specific countries and territories needing targeted interventions to enhance prevention, control, and healthcare efficiency [25]. Our findings indicate a higher prevalence and incidence of diabetes and kidney diseases in countries with higher SDI, which challenges the common assumption that better healthcare resources lead to lower disease burdens. There could be several explanations for this observation. Firstly, it is possible that diabetes and kidney diseases are better diagnosed and recorded in countries with higher SDI due to more accessible and sophisticated healthcare systems [26]. This could lead to an overrepresentation of cases in these countries [27]. Secondly, improved living standards, often associated with higher SDI, can lead to lifestyle changes such as increased calorie intake and reduced physical activity, which are known risk factors for diabetes and obesity-related kidney diseases. Additionally, the aging population in high SDI countries, which is a significant risk factor for kidney diseases, might also contribute to the observed burden [28,29]. It is important to note that while diabetes and kidney diseases are incurable, their management and progression can vary significantly based on the availability and quality of healthcare, which is generally better in high SDI countries [30,31]. This could potentially lead to a longer duration of the disease, increasing the overall burden measured in terms of DALYs.

Our analysis reveals distinct regional patterns: Oceania’s persistently high ASR likely reflects synergistic effects of rapid nutrition transition and genetic susceptibility [32]. Eastern Europe’s mortality rate spike (2012–2016 APC = 20.01%) coincides with healthcare system fragmentation post-Soviet transition, limiting access to renoprotective therapies [33]. South Asia’s absolute burden dominance necessitates decentralized screening programs leveraging community health workers [34], while high-SDI regions require integrated cardiorenal clinics to address multimorbidity.

Our analysis revealed a concerning temporal trend in socioeconomic disparities, with both the SII and concentration index values for diabetes and kidney disease burden in 2021 significantly exceeding their 1990 counterparts, demonstrating a progressive widening of SDI-related health inequalities across nations over the three-decade study period. These findings suggest a need for specific health service policies and resource allocation plans for various countries and territories to alleviate these disparities [35]. In high SDI countries, comprehensive disease management programs have shown success. For instance, the Chronic Disease Management Programme (CDMP) in Singapore provides essential subsidies that cover outpatient care, diagnostics, and medications, improving participation rates and reducing healthcare costs [36]. Similarly, the National Health Insurance (NHI) in Japan ensures comprehensive diabetes care, funding billions of yen annually for insulin, glucose monitors, and advanced technology like CGMs, which has reduced diabetes complications and maintained low hospitalization rates [37]. However, even in high-income countries, the majority of prevalent chronic kidney disease remains undiagnosed, highlighting the need for improved screening and awareness programs. In contrast, low SDI countries face challenges due to population growth and a shortage of medical resources. Successful initiatives include the Ayushman Bharat program in India, which provides free or subsidized diabetes treatment for low-income families, and the National Diabetes Prevention and Control Plan in the UAE, which supports public health campaigns and subsidizes essential medications, leading to better disease management [38].

Our decomposition analysis demonstrated a clear socioeconomic gradient in disease determinants, revealing that aging’s contribution to diabetes and kidney disease burden progressively increases with higher SDI levels while population factors diminish in importance. As nations transitioned from low to high SDI status, aging’s proportional contribution rose substantially across all metrics: from 9.99% to 35.67% for prevalence, 6.06% to 34.86% for incidence, 8.96% to 42.59% for DALYs, and strikingly from 11.29% to 70.00% for mortality rate. Conversely, population-related contributions showed consistent declines from 74.41% to 26.51% (prevalence), 61.58% to 25.06% (incidence), 92.64% to 24.80% (DALYs), and 99.16% to 27.50% (mortality rate). These patterns align with existing evidence, including Brazilian data showing a 118.6% increase in diabetes DALYs (1990–2015) where 72.1% of the rise was attributable to aging versus 42% from population growth [39], as well as global studies confirming the age-dependent escalation of diabetes and kidney disease incidence, mortality rate, and DALYs [40]. Countries with high SDI must prioritize obesity management due to its strong association with diabetes and kidney diseases. Practical strategies include implementing public health policies that promote physical activity and healthy eating, such as Norway’s integrated welfare system, which combines free nutritional education in schools, subsidized recreational facilities, and urban planning prioritizing walkability and cycling infrastructure [41]. Additionally, healthcare systems can integrate obesity screening and counseling into routine checkups, as seen in the successful ‘Healthy Weight for Life’ program in Australia [42]. Furthermore, health communication campaigns that raise awareness about the risks of obesity and the benefits of a healthy lifestyle can be effective, as demonstrated by the ‘Change4Life’ campaign in the UK [43]. These initiatives can significantly contribute to reducing the risk of diabetes and kidney diseases by addressing the underlying issue of obesity.

Given the substantial global burden of diabetes and kidney diseases, comprehensive prevention and management strategies must address both primary risk modification and secondary intervention [44,45]. Hypertension—a major contributor to kidney disease alongside diabetes—requires particular attention, as early detection and treatment can prevent or delay disease progression [46]. This urgency is underscored by prognostic evidence: albuminuria and impaired eGFR in diabetic patients individually correlate with 18% and 24% 10-year mortality rate respectively, but when coexisting, mortality rate escalates to nearly 50% [47,48]. Clinical guidelines (KDIGO) therefore recommend integrated approaches including (1): routine screening for kidney markers (albuminuria/eGFR) in high-risk populations (2); lifestyle interventions like sodium restriction (<2 g/day) and tailored protein intake (0.8 g/kg/day for non-dialysis patients) (3); minimum 150 min/week of physical activity [49]; and (4) evidence-based self-management programs [50]. Complementing these clinical measures, public health initiatives should promote population-level healthy behaviors to reduce modifiable risks like obesity and hypertension.

This study has certain limitations. First, while our decomposition analysis attributes changes in burden to broad demographic and epidemiological components, and our inequality analysis quantifies gradients by SDI, our methodological approach primarily describes associations and trends rather than establishing causal relationships adjusted for individual-level confounders. Factors such as genetic predisposition, specific environmental exposures, variations in healthcare access within SDI groups, and unmeasured behavioral risks could confound the observed associations and trends. Future research utilizing individual-level data with multivariate adjustment is warranted to elucidate these complex relationships. Second, the reliance on 2021 GBD data presents constraints, as the dataset may be outdated. In regions with underdeveloped healthcare systems, incomplete or inaccurate reporting could introduce estimation bias in modeling analyses. Third, variations in healthcare infrastructure and data collection protocols across countries may compromise the comparability of results. Fourth, the available disease burden data exhibits a significant time lag (currently limited to 1990–2021). Consequently, future real-world studies are needed to validate these findings and facilitate more precise, comprehensive assessments.

Despite these limitations, this study offers several notable strengths. Through comprehensive analyses—including descriptive, trend, decomposition, and cross-country inequality assessments—we have significantly advanced the understanding of diabetes and kidney disease epidemiology. These findings necessitate region-specific policies (1): High-SDI countries should implement mandatory geriatric DKD screening programs and value-based reimbursement for SGLT2i/GLP-1RA therapies (2); Low-middle-SDI regions require WHO-coordinated financing for urine albumin-to-creatinine ratio (ACR) testing and task-shifting to nurses (3); International funders should prioritize microfinancing for dialysis access in low-SDI nations with rising mortality rate (ASR) (e.g., Fiji).

## 5. Conclusion

Diabetes and kidney diseases remain major global public health challenges. Although their prevalence, incidence, DALYs, and mortality rates vary considerably across regions, these conditions have shown a consistent upward trend worldwide from 1990 to 2021. Notably, the burden of diabetes and kidney diseases has increasingly shifted toward high-SDI countries, where prevalence, incidence, DALYs, and mortality rates are now disproportionately concentrated. Over time, socioeconomic disparities—measured by the SDI—have further widened, exacerbating inequalities in disease burden between nations. The findings highlight the need for health decision-makers to prioritize research into targeted interventions and adapt healthcare systems to address the distinct patterns and inequalities in diabetes and kidney disease burden observed across nations.

## Supplementary Material

Supplementary information.docx

## Data Availability

The data used in the present study can be obtained from the Global Burden of Diseases, Injuries, and Risk Factors Study (GBD) 2021. (Available at: https://vizhub.healthdata.org/gbd-results/)
