# Supplementary material for: Global, regional, and national burden of diabetes and kidney diseases, 1990–2021: a trend and health inequality analyses based on the Global Burden of Disease Study 2021
Source: Ren Fail. 2025 Sep 29;47(1):2552956. doi: 10.1080/0886022X.2025.2552956 (PMC12486446; doi:10.1080/0886022X.2025.2552956)
Supplement: Supplementary information.docx [file IRNF_A_2552956_SM3026.docx]

**Supplementary Material**

**Table S1. The case number and ASR of incidence of diabetes and kidney diseases in 1990 and 2021, and its temporal trends from 1990 to 2021.**

| **location** | **1990** | | **2021** | | **EAPC (95% CI)** | **AAPC (95% CI)** | **P value** |
| --- | --- | --- | --- | --- | --- | --- | --- |
|  | **Case number (95% UI)** | **ASR (95% UI)** | **Case number (95% UI)** | **ASR (95% UI)** |  |  |  |
| **Global** | 16198875 | 372.15 | 44905586 | 527.33 | 1.12% | 1.13% | <0.001 |
|  | (12728191 to 19998124) | (291.92 to 460.08) | (36221360 to 54123171) | (424.64 to 636.71) | (1.10 to 1.13) | (1.09 to 1.17) |  |
| **Sex** |  |  |  |  |  |  |  |
| Male | 7712996 | 367.66 | 21777598 | 528.21 | 1.16% | 1.17% | <0.001 |
|  | (6080340 to 9512701) | (289.04 to 454.4) | (17584828 to 26285112) | (425.45 to 638.95) | (1.15 to 1.17) | (1.15 to 1.20) |  |
| Female | 8485879 | 375.47 | 23127988 | 525.49 | 1.08% | 1.09% | <0.001 |
|  | (6640418 to 10510731) | (293.75 to 465.19) | (18624449 to 27893117) | (422.88 to 634.4) | (1.06 to 1.09) | (1.06 to 1.12) |  |
| **SDI** |  |  |  |  |  |  |  |
| High SDI | 4538311 | 429.06 | 10766546 | 630.32 | 1.22% | 1.25% | <0.001 |
|  | (3548649 to 5633885) | (336.78 to 531.28) | (8759492 to 12948886) | (513.62 to 757.26) | (1.18 to 1.26) | (1.23 to 1.27) |  |
| High-middle SDI | 3512151 | 339.93 | 8238531 | 470.53 | 1.07% | 1.06% | <0.001 |
|  | (2733900 to 4356898) | (264.20 to 422.37) | (6554309 to 10024984) | (372.56 to 575.14) | (1.05 to 1.09) | (1.03 to 1.08) |  |
| Middle SDI | 4770888 | 370.01 | 14356966 | 525.61 | 1.12% | 1.14% | <0.001 |
|  | (3719943 to 5932678) | (288.36 to 460.09) | (11568232 to 17317676) | (422.27 to 635.71) | (1.09 to 1.14) | (1.12 to 1.16) |  |
| Low-middle SDI | 2508130 | 322.4 | 8585463 | 511.04 | 1.46% | 1.49% | <0.001 |
|  | (1981828 to 3093690) | (254.67 to 397.30) | (6868045 to 10461070) | (408.80 to 622.25) | (1.42 to 1.50) | (1.46 to 1.52) |  |
| Low SDI | 852218 | 284.25 | 2914117 | 406.8 | 1.10% | 1.16% | <0.001 |
|  | (681053 to 1043226) | (227.71 to 347.00) | (2332181 to 3561145) | (327.05 to 494.87) | (1.05 to 1.15) | (1.12 to 1.20) |  |
| **Region** |  |  |  |  |  |  |  |
| Andean Latin America | 74420 | 306.2 | 356407 | 577.14 | 2.23% | 2.07% | <0.001 |
|  | (58842 to 91962) | (241.61 to 378.64) | (286688 to 431688) | (463.63 to 699.90) | (2.14 to 2.31) | (2.04 to 2.11) |  |
| Australasia | 89961 | 384.18 | 243932 | 505.97 | 0.92% | 0.90% | <0.001 |
|  | (72178 to 110682) | (308.12 to 472.54) | (191807 to 298752) | (399.80 to 618.11) | (0.88 to 0.96) | (0.86 to 0.93) |  |
| Caribbean | 127330 | 441.99 | 376121 | 719.63 | 1.60% | 1.59% | <0.001 |
|  | (102661 to 152384) | (356.88 to 527.97) | (305450 to 449150) | (583.67 to 860.43) | (1.56 to 1.64) | (1.55 to 1.62) |  |
| Central Asia | 142559 | 248.98 | 439861 | 460.38 | 2.15% | 2% | <0.001 |
|  | (111703 to 175243) | (194.83 to 305.84) | (354277 to 525901) | (368.90 to 553.04) | (2.10 to 2.20) | (1.92 to 2.07) |  |
| Central Europe | 471241 | 322.81 | 920757 | 504.23 | 1.42% | 1.44% | <0.001 |
|  | (379530 to 567628) | (259.71 to 389.95) | (747776 to 1102533) | (411.10 to 602.39) | (1.34 to 1.49) | (1.38 to 1.51) |  |
| Central Latin America | 627981 | 585.85 | 2114344 | 806.56 | 1.03% | 1.04% | <0.001 |
|  | (497510 to 773587) | (464.43 to 720.66) | (1734350 to 2519590) | (660.39 to 962.53) | (0.99 to 1.07) | (0.93 to 1.16) |  |
| Central Sub-Saharan Africa | 85051 | 277.02 | 340254 | 406.02 | 1.25% | 1.24% | <0.001 |
|  | (67546 to 104731) | (222.28 to 338.19) | (268409 to 417163) | (323.48 to 493.98) | (1.19 to 1.31) | (1.20 to 1.28) |  |
| East Asia | 3574764 | 341.86 | 7819771 | 419.67 | 0.63% | 0.65% | <0.001 |
|  | (2676035 to 4589830) | (256.08 to 438.43) | (6043511 to 9777870) | (323.01 to 526.56) | (0.59 to 0.67) | (0.59 to 0.71) |  |
| Eastern Europe | 611452 | 234.82 | 1131517 | 390.57 | 1.66% | 1.65% | <0.001 |
|  | (481056 to 752033) | (184.51 to 289.74) | (893671 to 1386899) | (309.01 to 479.37) | (1.63 to 1.70) | (1.62 to 1.69) |  |
| Eastern Sub-Saharan Africa | 244391 | 241.69 | 717995 | 304.21 | 0.67% | 0.74% | <0.001 |
|  | (198338 to 294914) | (197.52 to 290.45) | (579511 to 867643) | (247.47 to 364.57) | (0.59 to 0.74) | (0.72 to 0.76) |  |
| High-income Asia Pacific | 963018 | 479.09 | 2160233 | 631.79 | 0.84% | 0.91% | <0.001 |
|  | (758405 to 1181982) | (375.96 to 590.00) | (1702471 to 2649413) | (500.63 to 772.07) | (0.78 to 0.90) | (0.85 to 0.98) |  |
| High-income North America | 1632507 | 494.26 | 4261155 | 768.6 | 1.45% | 1.45% | <0.001 |
|  | (1241144 to 2078478) | (379.00 to 625.32) | (3475431 to 5125576) | (627.61 to 923.56) | (1.39 to 1.51) | (1.42 to 1.48) |  |
| North Africa and Middle East | 943195 | 455.85 | 4824243 | 879.72 | 2.14% | 2.14% | <0.001 |
|  | (742964 to 1164209) | (356.69 to 564.98) | (3895323 to 5817768) | (706.04 to 1064.95) | (2.09 to 2.19) | (2.10 to 2.18) |  |
| Oceania | 20628 | 474.59 | 84115 | 744.72 | 1.43% | 1.46% | <0.001 |
|  | (16503 to 25154) | (382.78 to 574.85) | (66896 to 102020) | (594.15 to 901.45) | (1.38 to 1.48) | (1.43 to 1.48) |  |
| South Asia | 2365012 | 314 | 7868698 | 457.97 | 1.14% | 1.20% | <0.001 |
|  | (1834846 to 2960358) | (243.96 to 392.06) | (6129142 to 9811045) | (356.86 to 570.49) | (1.10 to 1.18) | (1.13 to 1.27) |  |
| Southeast Asia | 1110257 | 351.3 | 3973532 | 557.09 | 1.49% | 1.51% | <0.001 |
|  | (891041 to 1354663) | (282.14 to 427.86) | (3263771 to 4713916) | (455.82 to 663.24) | (1.46 to 1.53) | (1.46 to 1.55) |  |
| Southern Latin America | 178452 | 382.46 | 495967 | 601.97 | 1.55% | 1.48% | <0.001 |
|  | (139854 to 220896) | (299.24 to 474.14) | (397234 to 603102) | (483.13 to 730.81) | (1.47 to 1.63) | (1.44 to 1.52) |  |
| Southern Sub-Saharan Africa | 118529 | 365.4 | 375545 | 570.04 | 1.48% | 1.44% | <0.001 |
|  | (93672 to 146469) | (290.41 to 449.47) | (305274 to 452044) | (463.70 to 685.20) | (1.44 to 1.53) | (1.41 to 1.48) |  |
| Tropical Latin America | 465951 | 422.94 | 1467651 | 569.6 | 1% | 0.97% | <0.001 |
|  | (365956 to 576541) | (331.47 to 523.67) | (1176070 to 1790060) | (455.49 to 696.03) | (0.98 to 1.02) | (0.89 to 1.04) |  |
| Western Europe | 2018963 | 369.58 | 3726287 | 495.31 | 0.93% | 0.95% | <0.001 |
|  | (1562115 to 2526741) | (286.99 to 460.97) | (2984046 to 4549109) | (396.34 to 605.14) | (0.90 to 0.96) | (0.93 to 0.97) |  |
| Western Sub-Saharan Africa | 333212 | 290.88 | 1207200 | 428.82 | 1.26% | 1.26% | <0.001 |
|  | (263251 to 408848) | (230.16 to 356.19) | (964926 to 1474464) | (345.30 to 520.30) | (1.22 to 1.30) | (1.22 to 1.30) |  |

*ASR: age-standardized rate; EAPC: estimated annual percentage change; AAPC: average annual percent change; SDI: sociodemographic index; UI: uncertainty interval; CI: confidence interval.*

**Table S2. The case number and ASR of DALYs of diabetes and kidney diseases in 1990 and 2021, and its temporal trends from 1990 to 2021.**

| **location** | **1990** | | **2021** | | **EAPC (95% CI)** | **AAPC (95% CI)** | **P value** |
| --- | --- | --- | --- | --- | --- | --- | --- |
|  | **Case number (95% UI)** | **ASR (95% UI)** | **Case number (95% UI)** | **ASR (95% UI)** |  |  |  |
| **Global** | 48811345 | 1154.44 | 123704574 | 1449.38 | 0.69% | 0.74% | <0.001 |
|  | (43209260 to 54928854) | (1025.26 to 1296.14) | (107440163 to 143210093) | (1257.96 to 1677.64) | (0.66 to 0.72) | (0.69 to 0.79) |  |
| **Sex** |  |  |  |  |  |  |  |
| Male | 24828841 | 1247.53 | 64034137 | 1584.54 | 0.75% | 0.79% | <0.001 |
|  | (21577550 to 28186254) | (1094.20 to 1410.06) | (55408655 to 74243425) | (1371.97 to 1834.05) | (0.73 to 0.78) | (0.75 to 0.82) |  |
| Female | 23982504 | 1078.81 | 59670437 | 1329.51 | 0.61% | 0.68% | <0.001 |
|  | (21159260 to 27084502) | (952.66 to 1216.63) | (51619667 to 69422954) | (1149.08 to 1548.45) | (0.57 to 0.65) | (0.62 to 0.74) |  |
| **SDI** |  |  |  |  |  |  |  |
| High SDI | 8468414 | 795.94 | 20227912 | 1076.58 | 0.93% | 1% | <0.001 |
|  | (7406313 to 9706636) | (694.71 to 913.96) | (16682513 to 24375478) | (883.73 to 1308.60) | (0.86 to 1.00) | (0.85 to 1.14) |  |
| High-middle SDI | 8821537 | 884.35 | 18705569 | 1000.01 | 0.31% | 0.40% | <0.001 |
|  | (7682501 to 10134973) | (770.48 to 1014.06) | (15628302 to 22279210) | (832.20 to 1195.38) | (0.24 to 0.37) | (0.30 to 0.51) |  |
| Middle SDI | 15765050 | 1359.7 | 42853878 | 1589.81 | 0.47% | 0.51% | <0.001 |
|  | (13982805 to 17721237) | (1211.34 to 1522.72) | (37661582 to 49296691) | (1397.04 to 1825.79) | (0.43 to 0.51) | (0.44 to 0.57) |  |
| Low-middle SDI | 10357009 | 1457.81 | 29627564 | 1948.81 | 0.96% | 0.94% | <0.001 |
|  | (8942747 to 11777860) | (1284.18 to 1645.59) | (26087841 to 33834857) | (1722.00 to 2216.00) | (0.93 to 0.98) | (0.85 to 1.04) |  |
| Low SDI | 5336149 | 1927.52 | 12158943 | 2025.78 | 0.07% | 0.16% | <0.001 |
|  | (4636217 to 6039315) | (1706.49 to 2163.98) | (10516985 to 14123413) | (1771.88 to 2327.55) | (0.00 to 0.14) | (0.08 to 0.24) |  |
| **Region** |  |  |  |  |  |  |  |
| Andean Latin America | 341200 | 1442.81 | 1114154 | 1853.54 | 0.73% | 0.87% | <0.001 |
|  | (300019 to 387134) | (1273.74 to 1631.59) | (931419 to 1331804) | (1551.43 to 2213.27) | (0.55 to 0.90) | (0.49 to 1.24) |  |
| Australasia | 141116 | 619.72 | 347561 | 688.56 | 0.35% | 0.33% | 0.013 |
|  | (121694 to 163535) | (533.22 to 719.17) | (281235 to 421197) | (554.12 to 842.53) | (0.31 to 0.39) | (0.07 to 0.60) |  |
| Caribbean | 575493 | 2102.94 | 1303820 | 2460.31 | 0.55% | 0.52% | <0.001 |
|  | (510636 to 652366) | (1870.79 to 2376.18) | (1099781 to 1561151) | (2073.41 to 2949.28) | (0.50 to 0.60) | (0.48 to 0.57) |  |
| Central Asia | 423371 | 808.94 | 1235566 | 1396.97 | 1.47% | 1.73% | <0.001 |
|  | (361789 to 496362) | (689.13 to 949.99) | (1045030 to 1483117) | (1183.21 to 1672.41) | (1.20 to 1.75) | (1.59 to 1.88) |  |
| Central Europe | 1380442 | 950.01 | 2112197 | 1021.05 | 0.31% | 0.22% | 0.016 |
|  | (1203316 to 1588318) | (828.38 to 1093.28) | (1762437 to 2546437) | (846.52 to 1239.91) | (0.23 to 0.39) | (0.04 to 0.41) |  |
| Central Latin America | 2278298 | 2417.53 | 7788719 | 3038.39 | 0.73% | 0.70% | <0.001 |
|  | (2066877 to 2524644) | (2198.38 to 2668.33) | (6913359 to 8900228) | (2698.71 to 3468.17) | (0.65 to 0.82) | (0.50 to 0.90) |  |
| Central Sub-Saharan Africa | 716313 | 2617.06 | 1826599 | 2766.76 | 0.11% | 0.18% | <0.001 |
|  | (576956 to 877024) | (2138.04 to 3170.66) | (1443317 to 2322057) | (2201.40 to 3437.97) | (0.04 to 0.18) | (0.13 to 0.23) |  |
| East Asia | 9301856 | 974.9 | 19015190 | 926.89 | -0.27% | -0.16% | <0.001 |
|  | (7912383 to 10893735) | (832.27 to 1136.22) | (15469355 to 23160032) | (750.41 to 1132.99) | (-0.38 to -0.16) | (-0.25 to -0.07) |  |
| Eastern Europe | 1351411 | 511.9 | 2648573 | 806.35 | 0.91% | 1.54% | <0.001 |
|  | (1152455 to 1590018) | (436.72 to 601.98) | (2259126 to 3160252) | (685.38 to 964.90) | (0.58 to 1.24) | (1.19 to 1.89) |  |
| Eastern Sub-Saharan Africa | 2268980 | 2394.85 | 4400982 | 2150.6 | -0.55% | -0.35% | <0.001 |
|  | (1950114 to 2580731) | (2100.41 to 2697.03) | (3811611 to 5097400) | (1885.77 to 2455.64) | (-0.62 to -0.48) | (-0.42 to -0.29) |  |
| High-income Asia Pacific | 1602456 | 810.53 | 3454387 | 874.94 | 0.15% | 0.24% | <0.001 |
|  | (1371910 to 1873565) | (693.65 to 945.92) | (2709722 to 4352646) | (673.56 to 1127.16) | (0.07 to 0.22) | (0.16 to 0.33) |  |
| High-income North America | 2931701 | 872.38 | 8642623 | 1438.65 | 1.55% | 1.64% | <0.001 |
|  | (2568495 to 3363945) | (762.90 to 1003.23) | (7240688 to 10300263) | (1204.30 to 1720.22) | (1.42 to 1.68) | (1.53 to 1.75) |  |
| North Africa and Middle East | 2982821 | 1575.08 | 10480650 | 2193.1 | 1.26% | 1.10% | <0.001 |
|  | (2533599 to 3701346) | (1346.06 to 1969.96) | (8901020 to 12496521) | (1875.98 to 2591.28) | (1.16 to 1.36) | (1.05 to 1.15) |  |
| Oceania | 121766 | 3548.06 | 374152 | 4286.48 | 0.58% | 0.60% | <0.001 |
|  | (95259 to 152724) | (2835.45 to 4399.19) | (309369 to 452146) | (3582.30 to 5129.33) | (0.51 to 0.64) | (0.54 to 0.66) |  |
| South Asia | 8664309 | 1304.23 | 26223804 | 1694.84 | 0.81% | 0.87% | <0.001 |
|  | (7360128 to 9914308) | (1130.28 to 1477.87) | (22645367 to 30241117) | (1470.06 to 1944.59) | (0.73 to 0.89) | (0.69 to 1.05) |  |
| Southeast Asia | 5064144 | 1699.42 | 13973376 | 2076.12 | 0.59% | 0.63% | <0.001 |
|  | (4374478 to 5777938) | (1488.15 to 1930.96) | (12219301 to 15978517) | (1814.23 to 2372.57) | (0.56 to 0.61) | (0.60 to 0.67) |  |
| Southern Latin America | 596822 | 1289.77 | 1091540 | 1277.95 | -0.04% | 0% | 0.996 |
|  | (542044 to 657485) | (1169.60 to 1420.67) | (930312 to 1277237) | (1087.12 to 1499.21) | (-0.17 to 0.10) | (-0.24 to 0.25) |  |
| Southern Sub-Saharan Africa | 558788 | 1863.9 | 1826591 | 3060.68 | 1.87% | 1.59% | <0.001 |
|  | (494640 to 631393) | (1656.54 to 2095.61) | (1637255 to 2038910) | (2749.59 to 3402.99) | (1.52 to 2.23) | (1.19 to 2.00) |  |
| Tropical Latin America | 1684605 | 1667.91 | 4153494 | 1615.13 | -0.13% | -0.04% | 0.717 |
|  | (1517080 to 1874992) | (1500.81 to 1854.08) | (3611710 to 4772656) | (1404.37 to 1855.10) | (-0.18 to -0.08) | (-0.29 to 0.20) |  |
| Western Europe | 3788394 | 687.49 | 6460701 | 756.14 | 0.32% | 0.31% | <0.001 |
|  | (3299530 to 4339683) | (594.45 to 792.87) | (5193559 to 7863651) | (596.50 to 942.84) | (0.26 to 0.38) | (0.21 to 0.41) |  |
| Western Sub-Saharan Africa | 2037060 | 1877.29 | 5229895 | 2181.59 | 0.47% | 0.49% | <0.001 |
|  | (1685478 to 2377214) | (1605.89 to 2157.64) | (4357823 to 6259502) | (1863.42 to 2562.13) | (0.42 to 0.52) | (0.43 to 0.55) |  |

*DALYs: disability-adjusted life years; ASR: age-standardized rate; EAPC: estimated annual percentage change; AAPC: average annual percent change; SDI: sociodemographic index; UI: uncertainty interval; CI: confidence interval.*

**Table S3. The case number of deaths and ASR of mortality rate of diabetes and kidney diseases in 1990 and 2021, and its temporal trends from 1990 to 2021.**

| **location** | **1990** | | **2021** | | **EAPC (95% CI)** | **AAPC (95% CI)** | **P value** |
| --- | --- | --- | --- | --- | --- | --- | --- |
|  | **Case number (95% UI)** | **ASR (95% UI)** | **Case number (95% UI)** | **ASR (95% UI)** |  |  |  |
| **Global** | 1237570 | 33.32 | 3195034 | 38.24 | 0.45% | 0.47% | <0.001 |
|  | (1145414 to 1329536) | (30.66 to 35.79) | (2887971 to 3428787) | (34.42 to 41.08) | (0.40 to 0.50) | (0.37 to 0.57) |  |
| **Sex** |  |  |  |  |  |  |  |
| Male | 604699 | 37.20 | 1596685 | 42.98 | 0.50% | 0.50% | <0.001 |
|  | (543902 to 665124) | (33.54 to 40.92) | (1442168 to 1731136) | (38.60 to 46.60) | (0.45 to 0.55) | (0.37 to 0.62) |  |
| Female | 632871 | 30.67 | 1598349 | 34.58 | 0.36% | 0.40% | <0.001 |
|  | (575356 to 688002) | (27.72 to 33.35) | (1410886 to 1742121) | (30.55 to 37.69) | (0.31 to 0.41) | (0.30 to 0.49) |  |
| **SDI** |  |  |  |  |  |  |  |
| High SDI | 249033 | 22.64 | 550226 | 23.51 | 0.08% | 0.15% | 0.020 |
|  | (228949 to 260245) | (20.74 to 23.70) | (470596 to 597211) | (20.50 to 25.39) | (-0.03 to 0.19) | (0.02 to 0.27) |  |
| High-middle SDI | 219163 | 24.5 | 469466 | 24.46 | -0.03% | 0.01% | 0.883 |
|  | (201825 to 237309) | (22.31 to 26.57) | (414384 to 516751) | (21.51 to 26.95) | (-0.11 to 0.05) | (-0.17 to 0.19) |  |
| Middle SDI | 375717 | 40.82 | 1109358 | 44.56 | 0.32% | 0.34% | <0.001 |
|  | (346540 to 407857) | (37.53 to 44.34) | (1006287 to 1193935) | (40.13 to 48.05) | (0.25 to 0.38) | (0.29 to 0.38) |  |
| Low-middle SDI | 253839 | 44.85 | 762359 | 58.27 | 0.90% | 0.85% | <0.001 |
|  | (225092 to 284321) | (39.90 to 50.28) | (688912 to 838322) | (52.44 to 64.08) | (0.86 to 0.93) | (0.66 to 1.05) |  |
| Low SDI | 138123 | 65.51 | 300203 | 66.3 | 0% | 0.07% | 0.311 |
|  | (121504 to 155044) | (57.83 to 73.55) | (263652 to 340828) | (58.52 to 74.78) | (-0.10 to 0.09) | (-0.07 to 0.21) |  |
| **Region** |  |  |  |  |  |  |  |
| Andean Latin America | 9794 | 48.97 | 35661 | 62.01 | 0.70% | 0.75% | 0.017 |
|  | (8625 to 11112) | (43.15 to 55.52) | (28915 to 43089) | (50.33 to 74.82) | (0.45 to 0.94) | (0.13 to 1.37) |  |
| Australasia | 4508 | 20.02 | 11022 | 18.18 | -0.23% | -0.36% | 0.133 |
|  | (4035 to 4887) | (17.79 to 21.77) | (9300 to 12187) | (15.50 to 20.06) | (-0.37 to -0.10) | (-0.83 to 0.11) |  |
| Caribbean | 16249 | 64.64 | 33950 | 62.9 | -0.02% | -0.08% | 0.100 |
|  | (14900 to 17685) | (59.18 to 70.18) | (28699 to 39561) | (53.21 to 73.35) | (-0.08 to 0.05) | (-0.17 to 0.01) |  |
| Central Asia | 7456 | 15.33 | 23581 | 29.59 | 1.74% | 2.05% | <0.001 |
|  | (6915 to 8089) | (14.15 to 16.73) | (20757 to 26513) | (26.07 to 33.16) | (1.34 to 2.14) | (1.69 to 2.41) |  |
| Central Europe | 34425 | 24.11 | 54933 | 23.69 | 0.06% | -0.06% | 0.730 |
|  | (32836 to 35809) | (22.88 to 25.13) | (49111 to 59817) | (21.17 to 25.85) | (-0.08 to 0.19) | (-0.39 to 0.28) |  |
| Central Latin America | 60064 | 76.3 | 224427 | 91.28 | 0.68% | 0.49% | <0.001 |
|  | (57723 to 61945) | (72.89 to 78.85) | (199828 to 248972) | (81.26 to 101.16) | (0.56 to 0.79) | (0.24 to 0.74) |  |
| Central Sub-Saharan Africa | 18821 | 95.21 | 45330 | 95.84 | -0.07% | 0.01% | 0.865 |
|  | (14903 to 23347) | (76.11 to 116.84) | (34375 to 58638) | (72.78 to 122.20) | (-0.16 to 0.02) | (-0.10 to 0.12) |  |
| East Asia | 196077 | 25.8 | 416937 | 20.88 | -0.73% | -0.68% | <0.001 |
|  | (171197 to 225840) | (22.54 to 29.74) | (345053 to 495197) | (17.23 to 24.76) | (-0.88 to -0.58) | (-0.83 to -0.53) |  |
| Eastern Europe | 22225 | 8.24 | 62407 | 17.71 | 1.55% | 2.74% | <0.001 |
|  | (21481 to 22876) | (7.95 to 8.48) | (56495 to 67795) | (16.04 to 19.25) | (0.65 to 2.45) | (2.01 to 3.48) |  |
| Eastern Sub-Saharan Africa | 63297 | 89.9 | 122884 | 82.15 | -0.49% | -0.29% | <0.001 |
|  | (54843 to 71588) | (78.34 to 101.72) | (106605 to 141050) | (71.46 to 93.66) | (-0.56 to -0.42) | (-0.37 to -0.20) |  |
| High-income Asia Pacific | 40661 | 22.18 | 84848 | 13.72 | -1.64% | -1.57% | <0.001 |
|  | (36741 to 43557) | (19.78 to 23.85) | (67212 to 95880) | (11.28 to 15.37) | (-1.72 to -1.57) | (-1.83 to -1.31) |  |
| High-income North America | 84024 | 23.43 | 224733 | 32.82 | 0.90% | 1.13% | <0.001 |
|  | (76491 to 88165) | (21.38 to 24.58) | (196733 to 241379) | (29.07 to 35.10) | (0.74 to 1.04) | (0.95 to 1.31) |  |
| North Africa and Middle East | 81070 | 55.47 | 260688 | 67.06 | 0.89% | 0.66% | <0.001 |
|  | (68689 to 105548) | (46.72 to 73.27) | (224876 to 296843) | (57.80 to 76.01) | (0.74 to 1.04) | (0.47 to 0.86) |  |
| Oceania | 3231 | 120.44 | 8963 | 131.26 | 0.24% | 0.27% | <0.001 |
|  | (2465 to 4124) | (94.28 to 151.48) | (7295 to 11000) | (108.14 to 159.45) | (0.17 to 0.31) | (0.22 to 0.31) |  |
| South Asia | 200834 | 38.6 | 651837 | 49.31 | 0.78% | 0.85% | <0.001 |
|  | (174766 to 224110) | (33.61 to 42.99) | (575096 to 725594) | (43.42 to 54.85) | (0.66 to 0.89) | (0.44 to 1.27) |  |
| Southeast Asia | 126883 | 51.89 | 369543 | 61.73 | 0.55% | 0.58% | <0.001 |
|  | (111383 to 145545) | (45.75 to 59.63) | (323729 to 415701) | (53.85 to 69.45) | (0.50 to 0.60) | (0.54 to 0.63) |  |
| Southern Latin America | 20901 | 47.51 | 35027 | 38.85 | -0.59% | -0.70% | <0.001 |
|  | (19324 to 22341) | (43.65 to 50.86) | (31174 to 37942) | (34.65 to 42.07) | (-0.83 to -0.36) | (-1.09 to -0.32) |  |
| Southern Sub-Saharan Africa | 16235 | 64.4 | 56947 | 111.69 | 2.08% | 1.80% | <0.001 |
|  | (14377 to 18298) | (56.87 to 72.63) | (51638 to 62490) | (100.94 to 122.46) | (1.65 to 2.52) | (1.35 to 2.25) |  |
| Tropical Latin America | 42575 | 50.06 | 115016 | 46.05 | -0.22% | -0.19% | 0.314 |
|  | (39956 to 44468) | (46.30 to 52.54) | (103547 to 122069) | (41.29 to 48.94) | (-0.32 to -0.12) | (-0.56 to 0.18) |  |
| Western Europe | 134093 | 22.47 | 224930 | 18.71 | -0.40% | -0.57% | <0.001 |
|  | (122014 to 141040) | (20.39 to 23.66) | (186126 to 247835) | (15.80 to 20.49) | (-0.50 to -0.31) | (-0.82 to -0.31) |  |
| Western Sub-Saharan Africa | 54145 | 66.8 | 131370 | 76.32 | 0.42% | 0.43% | <0.001 |
|  | (45440 to 63021) | (57.13 to 76.95) | (109664 to 154844) | (65.07 to 88.28) | (0.36 to 0.47) | (0.36 to 0.51) |  |

*ASR: age-standardized rate; EAPC: estimated annual percentage change; AAPC: average annual percent change; SDI: sociodemographic index; UI: uncertainty interval; CI: confidence interval.*

**Table S4. The case number and ASR of prevalence of diabetes and kidney diseases in 1990 and 2021, and its temporal trends from 1990 to 2021 in 204 countries and territories.**

| **location** | **1990** | | **2021** | | **EAPC (95% CI)** | **AAPC (95% CI)** | **P value** |
| --- | --- | --- | --- | --- | --- | --- | --- |
|  | **Case number (95% UI)** | **ASR (95% UI)** | **Case number (95% UI)** | **ASR (95% UI)** |  |  |  |
| Afghanistan | 907742(803219 to 1025636) | 12806.04(11350.44 to 14434.66) | 3227289(2869344 to 3609927) | 20385.46(18532.13 to 22307.21) | 1.54 (1.51 to 1.58) | 1.51 (1.49 to 1.53) | <0.001 |
| Albania | 194802(170011 to 222440) | 8023.58(7091.57 to 9045.13) | 343144(306985 to 382158) | 8969.15(7940.29 to 10084.06) | 0.37 (0.35 to 0.38) | 0.36 (0.35 to 0.38) | <0.001 |
| Algeria | 1832288(1576101 to 2129461) | 11747.27(10266.37 to 13434.19) | 6692882(6014851 to 7425165) | 16477.34(14891.21 to 18181.27) | 1.06 (1.01 to 1.1) | 1.09 (1.07 to 1.12) | <0.001 |
| American Samoa | 4977(4414 to 5592) | 15854.64(14264.48 to 17572.98) | 13331(12153 to 14564) | 26368.5(24037.19 to 28812.84) | 1.6 (1.52 to 1.68) | 1.64 (1.6 to 1.68) | <0.001 |
| Andorra | 4459(3909 to 5072) | 7444.54(6549.94 to 8440.3) | 12895(11659 to 14216) | 9559.56(8567.3 to 10631.75) | 0.82 (0.78 to 0.85) | 0.81 (0.8 to 0.83) | <0.001 |
| Angola | 603612(525500 to 693360) | 11196.73(9946.33 to 12602.72) | 2337136(2070968 to 2632061) | 13508.58(12187.48 to 14953.58) | 0.63 (0.61 to 0.64) | 0.61 (0.6 to 0.62) | <0.001 |
| Antigua and Barbuda | 6285(5623 to 6992) | 11632.04(10430.68 to 12912.49) | 16632(15057 to 18254) | 15371.88(13910.51 to 16892.05) | 0.9 (0.89 to 0.91) | 0.9 (0.88 to 0.91) | <0.001 |
| Argentina | 2532920(2228318 to 2872078) | 7898.21(6944.46 to 8960.22) | 5532185(4936971 to 6174355) | 10411.53(9257.08 to 11660.57) | 0.92 (0.9 to 0.94) | 0.9 (0.87 to 0.92) | <0.001 |
| Armenia | 356706(314171 to 406433) | 11996.55(10641.05 to 13560.31) | 529471(477193 to 586016) | 13393.82(11990.86 to 14934.31) | 0.31 (0.26 to 0.36) | 0.36 (0.34 to 0.37) | <0.001 |
| Australia | 1439135(1273441 to 1619001) | 7526.96(6646.82 to 8489.61) | 3439335(3104072 to 3798349) | 8826.14(7865.35 to 9868.24) | 0.55 (0.53 to 0.56) | 0.5 (0.46 to 0.55) | <0.001 |
| Austria | 708635(621003 to 803533) | 6596.13(5733.65 to 7548.72) | 1212715(1093992 to 1339790) | 8110.8(7208.3 to 9086.01) | 0.69 (0.68 to 0.7) | 0.67 (0.65 to 0.69) | <0.001 |
| Azerbaijan | 661532(578665 to 756363) | 11634.56(10284.49 to 13172.06) | 1534392(1374557 to 1710952) | 13800.69(12401.28 to 15335.1) | 0.57 (0.56 to 0.57) | 0.55 (0.54 to 0.56) | <0.001 |
| Bahamas | 20798(18397 to 23396) | 10899.84(9767.81 to 12110.87) | 62927(56812 to 69453) | 14621.13(13218.62 to 16116.72) | 0.96 (0.96 to 0.97) | 0.95 (0.95 to 0.96) | <0.001 |
| Bahrain | 44553(37975 to 51962) | 14085.68(12494.33 to 15827.33) | 287512(257549 to 319896) | 20441.22(18691 to 22305.13) | 1.21 (1.18 to 1.25) | 1.21 (1.17 to 1.25) | <0.001 |
| Bangladesh | 6855598(5882901 to 7959568) | 10623.12(9275.16 to 12121.87) | 21643093(19377932 to 24219856) | 14097.51(12681.94 to 15701.91) | 0.89 (0.83 to 0.95) | 0.92 (0.9 to 0.94) | <0.001 |
| Barbados | 30906(27805 to 34209) | 11296.06(10122.9 to 12547.15) | 65501(59979 to 71083) | 14654.38(13297.48 to 16058.18) | 0.76 (0.72 to 0.81) | 0.84 (0.8 to 0.88) | <0.001 |
| Belarus | 1269999(1084846 to 1478182) | 10517.78(8943.03 to 12303.94) | 1555633(1359150 to 1775830) | 11434.54(9848.77 to 13237.29) | 0.22 (0.2 to 0.24) | 0.27 (0.25 to 0.28) | <0.001 |
| Belgium | 1052585(941503 to 1177689) | 7630.63(6775.64 to 8595.62) | 1791535(1629392 to 1961067) | 9687.12(8689.87 to 10732.27) | 0.77 (0.74 to 0.81) | 0.77 (0.74 to 0.81) | <0.001 |
| Belize | 11368(10037 to 12864) | 10128.47(9047.88 to 11329.13) | 49587(44669 to 55118) | 13892.77(12614.25 to 15326.65) | 1.06 (1.04 to 1.07) | 1.02 (1 to 1.04) | <0.001 |
| Benin | 245084(213177 to 281171) | 9517.48(8407.78 to 10759.77) | 956296(848856 to 1076116) | 12697.38(11447.55 to 14072.22) | 0.89 (0.84 to 0.94) | 0.93 (0.88 to 0.99) | <0.001 |
| Bermuda | 5816(5112 to 6585) | 8924.42(7869.29 to 10076.01) | 12050(10916 to 13233) | 11137.8(9958.4 to 12402.64) | 0.68 (0.65 to 0.72) | 0.72 (0.69 to 0.75) | <0.001 |
| Bhutan | 38989(33321 to 45348) | 10959.63(9580.4 to 12468.06) | 91512(80880 to 103238) | 12986.36(11569.41 to 14535.97) | 0.55 (0.54 to 0.57) | 0.55 (0.53 to 0.56) | <0.001 |
| Bolivia (Plurinational State of) | 328965(286142 to 377224) | 8349.88(7362.21 to 9444.33) | 1123190(995718 to 1263338) | 11089.49(9900.46 to 12388.63) | 0.94 (0.92 to 0.97) | 0.92 (0.88 to 0.95) | <0.001 |
| Bosnia and Herzegovina | 373626(330170 to 421124) | 8628.97(7659.64 to 9670.92) | 639854(583919 to 696749) | 11744.51(10580.28 to 12964.4) | 1.1 (1.06 to 1.15) | 1 (0.97 to 1.03) | <0.001 |
| Botswana | 72207(62288 to 83839) | 10139.64(8929.25 to 11536.13) | 228979(201857 to 258814) | 12226.1(10945.68 to 13610.32) | 0.64 (0.63 to 0.65) | 0.61 (0.6 to 0.62) | <0.001 |
| Brazil | 11343373(9941437 to 12937748) | 10688.78(9494.48 to 12038.08) | 29219894(26140056 to 32650532) | 11642.02(10408.44 to 13016.65) | 0.3 (0.27 to 0.32) | 0.3 (0.26 to 0.34) | <0.001 |
| Brunei Darussalam | 21202(18378 to 24393) | 13506.1(12061.45 to 15107.11) | 86877(78367 to 95759) | 19790.33(18038.54 to 21608.68) | 1.12 (1.06 to 1.17) | 1.24 (1.22 to 1.26) | <0.001 |
| Bulgaria | 974955(870994 to 1090880) | 8650.72(7675.79 to 9745.61) | 1268595(1163567 to 1377732) | 10761.45(9740.6 to 11839.31) | 0.74 (0.7 to 0.78) | 0.71 (0.69 to 0.73) | <0.001 |
| Burkina Faso | 440923(379369 to 509179) | 8453.1(7375.54 to 9630.67) | 1343232(1179712 to 1528136) | 10591.44(9460.63 to 11844.5) | 0.72 (0.68 to 0.77) | 0.73 (0.72 to 0.75) | <0.001 |
| Burundi | 232742(195199 to 276118) | 7310.3(6236.42 to 8530.92) | 629071(531703 to 741754) | 8321.21(7190.87 to 9605.12) | 0.41 (0.4 to 0.41) | 0.42 (0.41 to 0.43) | <0.001 |
| Cabo Verde | 22636(19813 to 25797) | 9448.09(8298.63 to 10727.92) | 66690(59490 to 74553) | 12905.71(11596.84 to 14326.48) | 1.11 (1.07 to 1.15) | 1.02 (1 to 1.03) | <0.001 |
| Cambodia | 635999(526262 to 761696) | 10592.51(8954.95 to 12428.35) | 1869686(1609088 to 2168696) | 12949.99(11292.75 to 14829.56) | 0.65 (0.62 to 0.67) | 0.66 (0.64 to 0.67) | <0.001 |
| Cameroon | 560867(487905 to 644145) | 9824.61(8687.1 to 11106.85) | 2292928(2033361 to 2578054) | 12588.64(11376.41 to 13898.48) | 0.84 (0.75 to 0.93) | 0.81 (0.74 to 0.87) | <0.001 |
| Canada | 2734790(2363245 to 3144666) | 8653.85(7465.21 to 9968.99) | 6978539(6309928 to 7680517) | 11822.42(10543.88 to 13198.4) | 0.98 (0.91 to 1.04) | 1.02 (1 to 1.04) | <0.001 |
| Central African Republic | 175020(153187 to 199818) | 11635.74(10363.44 to 13049.95) | 465338(415283 to 520761) | 14385.81(13044.59 to 15857.85) | 0.71 (0.7 to 0.73) | 0.68 (0.67 to 0.7) | <0.001 |
| Chad | 302253(261550 to 347647) | 8913.21(7810.63 to 10125.19) | 936119(819299 to 1064050) | 11081.15(9881.37 to 12377.57) | 0.7 (0.67 to 0.73) | 0.71 (0.7 to 0.71) | <0.001 |
| Chile | 942457(814382 to 1083919) | 8453.45(7381.93 to 9630.12) | 2787968(2502184 to 3099241) | 11525.64(10284.06 to 12885.87) | 1.06 (1.03 to 1.09) | 1 (0.95 to 1.05) | <0.001 |
| China | 96236211(83963212 to 109783046) | 9852.4(8685.22 to 11130.85) | 217243871(194995428 to 241358534) | 11477.23(10248.07 to 12817.45) | 0.58 (0.5 to 0.65) | 0.5 (0.44 to 0.56) | <0.001 |
| Colombia | 2392751(2110270 to 2705918) | 10942.3(9785.46 to 12208.9) | 6769458(6137615 to 7457034) | 12337.32(11177.45 to 13598.98) | 0.28 (0.23 to 0.34) | 0.4 (0.34 to 0.46) | <0.001 |
| Comoros | 20434(17272 to 23967) | 7762.22(6671.78 to 8957.58) | 56841(49538 to 65176) | 9543.66(8397.26 to 10836.38) | 0.68 (0.67 to 0.7) | 0.66 (0.63 to 0.69) | <0.001 |
| Congo | 148983(129600 to 171147) | 11066.25(9798.54 to 12490.73) | 492384(436402 to 555196) | 13323.36(11997.07 to 14801.61) | 0.6 (0.58 to 0.61) | 0.6 (0.59 to 0.61) | <0.001 |
| Cook Islands | 2332(2079 to 2595) | 15993.49(14372.47 to 17668.99) | 5413(4969 to 5854) | 24069.23(21933 to 26227.54) | 1.2 (1.12 to 1.28) | 1.32 (1.29 to 1.34) | <0.001 |
| Costa Rica | 254103(224802 to 286154) | 12218.19(10946.06 to 13583.78) | 805793(732515 to 882917) | 14815.98(13453.72 to 16249.19) | 0.65 (0.63 to 0.68) | 0.63 (0.6 to 0.67) | <0.001 |
| Coted'Ivoire | 591680(507118 to 685894) | 9537.8(8367.76 to 10811.87) | 1981863(1742227 to 2243932) | 11884.41(10638.4 to 13224.19) | 0.72 (0.72 to 0.73) | 0.71 (0.71 to 0.72) | <0.001 |
| Croatia | 507380(451486 to 568896) | 8660.46(7680.36 to 9739.92) | 745382(677828 to 816909) | 10175.95(9107.75 to 11340.15) | 0.51 (0.48 to 0.54) | 0.51 (0.48 to 0.54) | <0.001 |
| Cuba | 984804(866854 to 1115029) | 9216.17(8137.54 to 10403.63) | 1888142(1704798 to 2078336) | 11266.19(10085.01 to 12510.46) | 0.59 (0.55 to 0.63) | 0.65 (0.64 to 0.66) | <0.001 |
| Cyprus | 77091(69468 to 85189) | 9365.24(8430.16 to 10361.15) | 204300(185263 to 224726) | 10653.15(9590.82 to 11799.79) | 0.37 (0.34 to 0.4) | 0.42 (0.41 to 0.43) | <0.001 |
| Czechia | 1099151(981553 to 1228039) | 8603.64(7636.36 to 9667.41) | 1846421(1677351 to 2015304) | 10292.39(9214.71 to 11405.89) | 0.63 (0.6 to 0.66) | 0.58 (0.56 to 0.6) | <0.001 |
| Democratic People's Republic of Korea | 1564352(1368646 to 1784394) | 8697.5(7659.25 to 9857.57) | 3502213(3133715 to 3885952) | 10862.38(9703.53 to 12072.1) | 0.66 (0.63 to 0.68) | 0.72 (0.71 to 0.73) | <0.001 |
| Democratic Republic of the Congo | 2178076(1886407 to 2507196) | 10756.17(9501.91 to 12137.12) | 6438095(5713020 to 7256545) | 12497.25(11306.93 to 13824.57) | 0.41 (0.39 to 0.43) | 0.48 (0.47 to 0.5) | <0.001 |
| Denmark | 505192(446253 to 570574) | 7011.01(6146.42 to 7983.95) | 847267(769054 to 933766) | 8937.68(8018.04 to 9965.39) | 0.77 (0.76 to 0.79) | 0.78 (0.77 to 0.8) | <0.001 |
| Djibouti | 15814(13092 to 19011) | 6817.23(5795.27 to 7987.29) | 77761(66067 to 90941) | 8320.98(7210.27 to 9548.54) | 0.67 (0.66 to 0.68) | 0.64 (0.62 to 0.66) | <0.001 |
| Dominica | 7239(6497 to 8061) | 12035.74(10822.85 to 13382.91) | 13102(11898 to 14395) | 16192.92(14668.64 to 17826.74) | 0.94 (0.93 to 0.96) | 0.96 (0.95 to 0.97) | <0.001 |
| Dominican Republic | 450861(393637 to 514414) | 9623.01(8520.5 to 10825.68) | 1492464(1335685 to 1657665) | 14050.28(12611.32 to 15561.63) | 1.29 (1.26 to 1.32) | 1.23 (1.21 to 1.24) | <0.001 |
| Ecuador | 547521(475368 to 628754) | 8393.98(7389.11 to 9504.82) | 2089304(1881317 to 2310827) | 12221.66(11033.57 to 13483.47) | 1.28 (1.25 to 1.31) | 1.22 (1.2 to 1.23) | <0.001 |
| Egypt | 3583224(3028020 to 4227867) | 10029.84(8630.88 to 11629.08) | 12277553(10822000 to 13872969) | 15389.6(13744.57 to 17164.28) | 1.44 (1.36 to 1.51) | 1.39 (1.36 to 1.42) | <0.001 |
| El Salvador | 326116(285591 to 372056) | 9365.35(8286.47 to 10573.86) | 786922(710862 to 872427) | 12621.3(11418.06 to 13974.3) | 1.01 (0.97 to 1.05) | 0.98 (0.9 to 1.05) | <0.001 |
| Equatorial Guinea | 25799(22472 to 29560) | 10902.97(9641.16 to 12299.73) | 115702(101475 to 130971) | 13850.09(12461.89 to 15321.99) | 0.82 (0.8 to 0.83) | 0.77 (0.75 to 0.79) | <0.001 |
| Eritrea | 130095(109162 to 154211) | 7278.93(6251.2 to 8438.02) | 387578(334138 to 447989) | 9112.23(8000.32 to 10352.24) | 0.75 (0.74 to 0.76) | 0.73 (0.72 to 0.74) | <0.001 |
| Estonia | 205443(177138 to 237459) | 10924.62(9360.17 to 12706.27) | 264409(236755 to 294141) | 12648.19(11120.76 to 14327.21) | 0.48 (0.47 to 0.49) | 0.48 (0.46 to 0.49) | <0.001 |
| Eswatini | 41348(35765 to 47658) | 10592.05(9374.33 to 11949.47) | 99281(87730 to 111724) | 13492.55(12167.51 to 14900.08) | 0.86 (0.83 to 0.89) | 0.79 (0.77 to 0.81) | <0.001 |
| Ethiopia | 2105312(1801233 to 2452258) | 7625.12(6628.17 to 8752.4) | 5511910(4707646 to 6418629) | 8451.55(7362.95 to 9664.57) | 0.32 (0.3 to 0.34) | 0.33 (0.32 to 0.34) | <0.001 |
| Fiji | 72680(64021 to 81988) | 14467.35(12949.81 to 16068.24) | 187105(170326 to 204695) | 21652(19832.22 to 23555.97) | 1.24 (1.22 to 1.26) | 1.31 (1.29 to 1.33) | <0.001 |
| Finland | 556951(499445 to 619875) | 8589.6(7666.49 to 9608.22) | 1044028(956579 to 1135136) | 11354.38(10275.15 to 12494.75) | 0.86 (0.79 to 0.93) | 0.9 (0.88 to 0.92) | <0.001 |
| France | 4324679(3822498 to 4869734) | 5745.04(5044.86 to 6516.63) | 8209717(7440495 to 9031821) | 7460.66(6664.74 to 8318.15) | 0.91 (0.84 to 0.98) | 0.86 (0.79 to 0.93) | <0.001 |
| Gabon | 72399(63676 to 82137) | 11213.7(9963.05 to 12589.19) | 184278(164961 to 205666) | 14089.05(12767.84 to 15543.97) | 0.75 (0.74 to 0.76) | 0.74 (0.73 to 0.75) | <0.001 |
| Gambia | 45792(39109 to 53338) | 9028.02(7886.36 to 10291.07) | 160864(140899 to 182666) | 11526.42(10285.05 to 12861.85) | 0.82 (0.81 to 0.83) | 0.8 (0.79 to 0.8) | <0.001 |
| Georgia | 713208(626918 to 809911) | 11890.11(10433.84 to 13523.33) | 730458(662595 to 802505) | 14400.27(12931.5 to 15985.32) | 0.64 (0.62 to 0.66) | 0.62 (0.59 to 0.65) | <0.001 |
| Germany | 7770417(6944915 to 8671064) | 6780.22(6008.51 to 7636.58) | 14106959(12995866 to 15285102) | 9183.84(8326.12 to 10104.7) | 0.94 (0.83 to 1.05) | 0.99 (0.97 to 1.01) | <0.001 |
| Ghana | 693301(588448 to 813466) | 8005.5(6922.35 to 9220.02) | 2449046(2142370 to 2791796) | 10516.68(9334.77 to 11821.19) | 0.91 (0.88 to 0.94) | 0.88 (0.87 to 0.89) | <0.001 |
| Greece | 1101205(981919 to 1231739) | 8013.6(7099.06 to 9020.23) | 1798708(1641468 to 1967429) | 9909.04(8904.71 to 10986.92) | 0.66 (0.64 to 0.68) | 0.69 (0.67 to 0.7) | <0.001 |
| Greenland | 3259(2719 to 3895) | 7542.51(6448.34 to 8790.05) | 6534(5775 to 7341) | 9829.13(8670.42 to 11069.48) | 0.83 (0.75 to 0.9) | 0.86 (0.84 to 0.88) | <0.001 |
| Grenada | 8531(7678 to 9492) | 12189.14(10971.21 to 13560.21) | 19217(17382 to 21157) | 16495.49(14932.03 to 18148.13) | 0.97 (0.96 to 0.98) | 0.97 (0.96 to 0.99) | <0.001 |
| Guam | 12137(10582 to 13916) | 11529.87(10200.7 to 13012.85) | 29148(26358 to 32192) | 15342.29(13813.48 to 17020.86) | 0.89 (0.87 to 0.92) | 0.93 (0.91 to 0.95) | <0.001 |
| Guatemala | 465022(410628 to 526243) | 10585.89(9481.8 to 11797.82) | 1978957(1795837 to 2180033) | 15749.86(14392.16 to 17225.9) | 1.42 (1.36 to 1.48) | 1.31 (1.23 to 1.39) | <0.001 |
| Guinea | 337983(294005 to 388468) | 8889.88(7807.91 to 10117.38) | 834213(734410 to 947559) | 10886.45(9728.24 to 12166.44) | 0.63 (0.6 to 0.66) | 0.65 (0.63 to 0.67) | <0.001 |
| Guinea-Bissau | 52695(45769 to 60483) | 9871.96(8711.55 to 11159.81) | 139880(123513 to 157968) | 12307.81(11088.27 to 13643.14) | 0.72 (0.71 to 0.72) | 0.72 (0.7 to 0.73) | <0.001 |
| Guyana | 68266(60409 to 76494) | 13985.01(12550.86 to 15467.28) | 142268(129223 to 156503) | 19914.03(18160.92 to 21828.55) | 1.15 (1.13 to 1.18) | 1.14 (1.12 to 1.16) | <0.001 |
| Haiti | 481543(425361 to 542155) | 12103.27(10822.62 to 13469.86) | 1506603(1338642 to 1683075) | 16046.94(14441.31 to 17747.52) | 0.9 (0.89 to 0.91) | 0.91 (0.91 to 0.92) | <0.001 |
| Honduras | 291211(256230 to 330250) | 11428.91(10199.61 to 12776.28) | 1128015(1011497 to 1255652) | 14870.19(13468.05 to 16404.83) | 0.88 (0.86 to 0.91) | 0.86 (0.78 to 0.94) | <0.001 |
| Hungary | 1186402(1061872 to 1320147) | 8819.14(7839.4 to 9882.52) | 1659283(1510522 to 1816135) | 10187.57(9147.04 to 11303.91) | 0.46 (0.39 to 0.54) | 0.45 (0.39 to 0.51) | <0.001 |
| Iceland | 18041(15922 to 20327) | 6498.99(5726.01 to 7336.94) | 42146(38259 to 46374) | 8617.55(7749.96 to 9561.94) | 0.91 (0.87 to 0.96) | 0.91 (0.89 to 0.94) | <0.001 |
| India | 73209718(62499848 to 85465545) | 12274.17(10638.9 to 14112.01) | 188067927(164552200 to 214289528) | 14022.03(12343.88 to 15882.29) | 0.36 (0.3 to 0.41) | 0.45 (0.35 to 0.55) | <0.001 |
| Indonesia | 15435018(12872684 to 18380737) | 11841.19(10058.28 to 13857.47) | 37899779(32452217 to 44052318) | 13747.62(11881.45 to 15842.73) | 0.34 (0.28 to 0.41) | 0.5 (0.45 to 0.56) | <0.001 |
| Iran (Islamic Republic of) | 3753209(3242436 to 4346064) | 11301.38(9925.54 to 12874.54) | 12492223(11121662 to 14031850) | 14351.18(12854.11 to 16024.45) | 0.78 (0.74 to 0.81) | 0.76 (0.73 to 0.8) | <0.001 |
| Iraq | 1527708(1334051 to 1745617) | 14569.54(12940.45 to 16357.85) | 6686234(6020552 to 7415419) | 21211.01(19337.89 to 23241.13) | 1.26 (1.23 to 1.3) | 1.22 (1.21 to 1.23) | <0.001 |
| Ireland | 327319(287863 to 369770) | 8444.96(7411.25 to 9566.23) | 678293(618898 to 742390) | 9913.2(8947.88 to 10965.88) | 0.55 (0.52 to 0.57) | 0.52 (0.51 to 0.54) | <0.001 |
| Israel | 415110(371824 to 461948) | 8670.18(7762.26 to 9654.15) | 1134981(1028600 to 1245869) | 10126.73(9113.91 to 11180.91) | 0.51 (0.46 to 0.56) | 0.5 (0.48 to 0.52) | <0.001 |
| Italy | 6437616(5743135 to 7210551) | 8141.52(7201.81 to 9193.58) | 9912115(8949723 to 10974502) | 9008.83(7988.66 to 10146.31) | 0.31 (0.25 to 0.36) | 0.31 (0.23 to 0.39) | <0.001 |
| Jamaica | 188682(167947 to 211578) | 10143.99(9069.82 to 11320.96) | 406007(365808 to 447634) | 13090.85(11801.64 to 14426.81) | 0.78 (0.74 to 0.81) | 0.83 (0.76 to 0.89) | <0.001 |
| Japan | 17938829(15831932 to 20298678) | 11202.42(9835.38 to 12742.43) | 31882150(29024163 to 34904947) | 12561.64(11166.17 to 14076.38) | 0.28 (0.24 to 0.33) | 0.38 (0.32 to 0.43) | <0.001 |
| Jordan | 273694(237432 to 314348) | 14185.93(12654.74 to 15852.95) | 1883505(1689629 to 2093542) | 19274.89(17538.33 to 21128.21) | 1.04 (1.02 to 1.06) | 1.01 (0.97 to 1.05) | <0.001 |
| Kazakhstan | 1718147(1510270 to 1952043) | 12363.9(10953.5 to 13938.94) | 2817714(2541949 to 3118734) | 14932.73(13497.64 to 16494.86) | 0.65 (0.64 to 0.66) | 0.61 (0.6 to 0.63) | <0.001 |
| Kenya | 825948(683039 to 992360) | 6831.5(5771.31 to 8049.78) | 2479614(2071568 to 2948955) | 7526.37(6423.6 to 8782.37) | 0.3 (0.28 to 0.31) | 0.31 (0.3 to 0.32) | <0.001 |
| Kiribati | 6886(6085 to 7770) | 14117.14(12632.11 to 15732.13) | 18938(17153 to 20852) | 20224.93(18492.93 to 22059.53) | 1.19 (1.14 to 1.24) | 1.17 (1.15 to 1.18) | <0.001 |
| Kuwait | 159951(137781 to 185650) | 14354.97(12752.38 to 16142.1) | 936344(834765 to 1044384) | 20451.26(18494.54 to 22501.54) | 1.23 (1.21 to 1.25) | 1.16 (1.13 to 1.19) | <0.001 |
| Kyrgyzstan | 384229(337315 to 435621) | 11586.79(10249.29 to 13035.37) | 720120(637967 to 807652) | 12815.97(11444.43 to 14246.62) | 0.32 (0.3 to 0.33) | 0.32 (0.31 to 0.34) | <0.001 |
| Lao People's Democratic Republic | 316649(265791 to 373567) | 12200.79(10415.33 to 14175.46) | 849353(733356 to 979623) | 14594.6(12825.1 to 16560.61) | 0.57 (0.54 to 0.59) | 0.58 (0.57 to 0.59) | <0.001 |
| Latvia | 350026(300952 to 404029) | 10769.33(9187.35 to 12518.93) | 387996(346160 to 432875) | 12701.21(11106 to 14444.44) | 0.54 (0.53 to 0.55) | 0.53 (0.52 to 0.55) | <0.001 |
| Lebanon | 303561(266022 to 345722) | 12616.99(11136.19 to 14266.25) | 1051694(948175 to 1162155) | 17519.7(15806.04 to 19351.15) | 1.13 (1.1 to 1.16) | 1.08 (1.04 to 1.12) | <0.001 |
| Lesotho | 91048(78885 to 105026) | 9508.02(8335.2 to 10840.67) | 159140(140461 to 179914) | 12188.63(10928.1 to 13564.56) | 0.87 (0.84 to 0.9) | 0.81 (0.8 to 0.82) | <0.001 |
| Liberia | 136592(118224 to 157222) | 9482.7(8331.53 to 10765.59) | 398693(350749 to 452408) | 12084(10828.24 to 13478.29) | 0.83 (0.82 to 0.84) | 0.79 (0.78 to 0.8) | <0.001 |
| Libya | 294318(251220 to 344332) | 11799.36(10268.4 to 13532.84) | 1147621(1019336 to 1289174) | 17290.99(15525.37 to 19239.66) | 1.34 (1.31 to 1.38) | 1.25 (1.24 to 1.27) | <0.001 |
| Lithuania | 454981(389491 to 529095) | 10741.36(9151.63 to 12549.15) | 541938(481582 to 607506) | 12257.73(10676.18 to 14021.91) | 0.42 (0.41 to 0.43) | 0.42 (0.4 to 0.45) | <0.001 |
| Luxembourg | 39539(34844 to 44694) | 7847.07(6879.53 to 8915.05) | 89611(80406 to 99482) | 9647.39(8589.14 to 10791.19) | 0.68 (0.65 to 0.71) | 0.68 (0.64 to 0.71) | <0.001 |
| Madagascar | 475686(396401 to 567581) | 6897.31(5851.21 to 8098.47) | 1369025(1155457 to 1610049) | 7957.65(6859.61 to 9171.82) | 0.46 (0.44 to 0.48) | 0.46 (0.45 to 0.47) | <0.001 |
| Malawi | 383828(317326 to 460438) | 6996.38(5904.24 to 8234.97) | 884685(734311 to 1056842) | 7789.19(6617.11 to 9106.63) | 0.34 (0.33 to 0.35) | 0.35 (0.33 to 0.36) | <0.001 |
| Malaysia | 1626603(1384088 to 1905463) | 13401.66(11643.54 to 15387.82) | 4949274(4330888 to 5632866) | 15853.6(13992.74 to 17898.4) | 0.62 (0.58 to 0.67) | 0.54 (0.48 to 0.6) | <0.001 |
| Maldives | 14663(12208 to 17428) | 12101.34(10323.29 to 14075.02) | 67987(57791 to 79814) | 13820.53(12039.39 to 15832.91) | 0.41 (0.38 to 0.44) | 0.44 (0.43 to 0.44) | <0.001 |
| Mali | 526183(464379 to 594399) | 10653.68(9521.11 to 11896.32) | 1803990(1601014 to 2021689) | 14425.91(13016.8 to 15938.49) | 1.05 (1 to 1.09) | 0.98 (0.95 to 1.01) | <0.001 |
| Malta | 32861(29126 to 36969) | 7908.1(7001.73 to 8908.17) | 86368(78822 to 93875) | 11359.09(10235.3 to 12515.93) | 1.13 (1.01 to 1.26) | 1.18 (1.16 to 1.2) | <0.001 |
| Marshall Islands | 3883(3441 to 4356) | 16381.07(14769.67 to 18120.65) | 12725(11511 to 14061) | 26706.01(24380.72 to 29231.62) | 1.66(1.6 to 1.72) | 1.58 (1.54 to 1.62) | <0.001 |
| Mauritania | 109513(94379 to 126566) | 8919.01(7794.56 to 10166.11) | 270379(236520 to 308454) | 9884.81(8776.92 to 11113.61) | 0.29 (0.25 to 0.32) | 0.33 (0.31 to 0.34) | <0.001 |
| Mauritius | 126516(109206 to 146337) | 14259.6(12510.41 to 16224.65) | 327408(296745 to 359172) | 19102.25(17195.15 to 21118.12) | 1 (0.96 to 1.04) | 0.96 (0.94 to 0.98) | <0.001 |
| Mexico | 7634754(6826593 to 8538617) | 14332.3(12968.72 to 15843.33) | 21035894(19105990 to 23128510) | 15823.97(14397.12 to 17368.37) | 0.27 (0.24 to 0.31) | 0.32 (0.3 to 0.34) | <0.001 |
| Micronesia (Federated States of) | 8184(7249 to 9225) | 13166.98(11799.84 to 14680.97) | 17809(16200 to 19536) | 20028.7(18341.59 to 21831.72) | 1.49 (1.39 to 1.58) | 1.37 (1.29 to 1.46) | <0.001 |
| Monaco | 3980(3526 to 4468) | 7100.46(6195.87 to 8108.67) | 6528(5919 to 7174) | 9088.8(8094.32 to 10157.85) | 0.8 (0.77 to 0.83) | 0.8 (0.79 to 0.82) | <0.001 |
| Mongolia | 157315(137091 to 180773) | 11907.28(10524.8 to 13476.99) | 372081(330631 to 419331) | 13217.61(11833.46 to 14761.14) | 0.34 (0.32 to 0.35) | 0.34 (0.33 to 0.34) | <0.001 |
| Montenegro | 58749(52183 to 65918) | 9274.83(8253.66 to 10388.61) | 97943(88823 to 107768) | 11108.41(9984.31 to 12328.43) | 0.58 (0.56 to 0.6) | 0.58 (0.57 to 0.6) | <0.001 |
| Morocco | 2122754(1824356 to 2469052) | 12144.3(10590.47 to 13929.72) | 7226988(6486454 to 8012195) | 19416.07(17483.29 to 21470.6) | 1.55 (1.52 to 1.58) | 1.53 (1.52 to 1.53) | <0.001 |
| Mozambique | 564693(469245 to 674133) | 7112.4(6008.87 to 8358.64) | 1506017(1279687 to 1769563) | 8849.39(7671.17 to 10186.48) | 0.74 (0.72 to 0.75) | 0.71 (0.69 to 0.72) | <0.001 |
| Myanmar | 3657358(3102367 to 4285703) | 12772.09(11015.96 to 14731.39) | 8334720(7349823 to 9414120) | 15775.25(13998.55 to 17711.18) | 0.66 (0.65 to 0.68) | 0.69 (0.67 to 0.71) | <0.001 |
| Namibia | 80799(69500 to 93477) | 10113.62(8868.82 to 11478.83) | 195169(171054 to 222123) | 11359.25(10116.21 to 12732.35) | 0.39 (0.38 to 0.4) | 0.38 (0.37 to 0.39) | <0.001 |
| Nauru | 949(838 to 1071) | 14694.83(13165.88 to 16347.59) | 1727(1564 to 1902) | 21798.71(19975.55 to 23755.9) | 1.17 (1.1 to 1.25) | 1.27 (1.23 to 1.31) | <0.001 |
| Nepal | 1471761(1300242 to 1663199) | 12552.2(11238.73 to 13997.61) | 4188297(3787697 to 4632164) | 15868.47(14439.63 to 17452.57) | 0.66 (0.61 to 0.71) | 0.75 (0.7 to 0.79) | <0.001 |
| Netherlands | 1472758(1296481 to 1665583) | 7861.92(6886.97 to 8936.85) | 2606139(2349166 to 2884279) | 9380.27(8328.35 to 10535.19) | 0.48 (0.43 to 0.52) | 0.56 (0.54 to 0.58) | <0.001 |
| New Zealand | 315075(278104 to 357104) | 8305.01(7316.8 to 9434.92) | 703884(641258 to 770662) | 9553.07(8630.49 to 10555.39) | 0.54 (0.44 to 0.63) | 0.45 (0.41 to 0.5) | <0.001 |
| Nicaragua | 253196(224456 to 284941) | 12667.19(11410.34 to 14032.5) | 897359(817430 to 983809) | 16050.29(14720.07 to 17473.43) | 0.74 (0.72 to 0.75) | 0.77 (0.71 to 0.84) | <0.001 |
| Niger | 341116(294592 to 394200) | 8713.94(7672.93 to 9872.38) | 1256817(1101031 to 1430348) | 10715.35(9566.11 to 11979.41) | 0.67 (0.66 to 0.69) | 0.67 (0.67 to 0.68) | <0.001 |
| Nigeria | 5698147(5001437 to 6494541) | 10628.69(9456.12 to 11947.26) | 14986160(13130951 to 17056299) | 11842.3(10582.01 to 13235.6) | 0.41 (0.38 to 0.43) | 0.35 (0.32 to 0.39) | <0.001 |
| Niue | 317(285 to 351) | 14975.44(13432.57 to 16636.68) | 471(431 to 514) | 23715.82(21575.66 to 25994.85) | 1.5 (1.46 to 1.54) | 1.49 (1.48 to 1.5) | <0.001 |
| North Macedonia | 196309(175459 to 218967) | 10161.14(9112.35 to 11299.6) | 394888(359168 to 431290) | 12555.58(11360.12 to 13794.86) | 0.71 (0.67 to 0.75) | 0.69 (0.67 to 0.7) | <0.001 |
| Northern Mariana Islands | 4196(3640 to 4803) | 12501.26(11103.35 to 14009.63) | 9190(8306 to 10115) | 16741.73(15111.78 to 18448.69) | 0.89 (0.86 to 0.91) | 0.95 (0.93 to 0.96) | <0.001 |
| Norway | 469890(418554 to 525591) | 8118.67(7167.11 to 9162.59) | 761860(683980 to 844950) | 9426.54(8365.25 to 10567.46) | 0.44 (0.41 to 0.48) | 0.48 (0.43 to 0.53) | <0.001 |
| Oman | 131382(111545 to 154298) | 11647.66(10196.05 to 13277.86) | 574314(500321 to 657254) | 15709.97(14054.67 to 17520.87) | 0.87 (0.81 to 0.93) | 0.95 (0.93 to 0.97) | <0.001 |
| Pakistan | 7615885(6550178 to 8803952) | 10933.61(9529.95 to 12483.44) | 24308175(21432066 to 27471850) | 14566.02(13025.46 to 16245.87) | 1.02 (0.98 to 1.06) | 0.92 (0.88 to 0.96) | <0.001 |
| Palau | 1805(1606 to 2029) | 14844.48(13343.66 to 16534.43) | 5291(4869 to 5722) | 22514.22(20668.12 to 24451.33) | 1.37 (1.34 to 1.39) | 1.35 (1.33 to 1.37) | <0.001 |
| Palestine | 136557(117803 to 158237) | 12310.66(10828.73 to 13985.08) | 553722(489995 to 623610) | 16262.81(14648.3 to 17985.9) | 0.93 (0.87 to 0.98) | 0.9 (0.89 to 0.92) | <0.001 |
| Panama | 189585(166603 to 215448) | 10907.56(9695.41 to 12258.09) | 608573(549713 to 671782) | 13789.46(12454.1 to 15223.45) | 0.78 (0.76 to 0.8) | 0.77 (0.71 to 0.82) | <0.001 |
| Papua New Guinea | 292662(256015 to 333404) | 11664.72(10370.22 to 13093.25) | 1262420(1131365 to 1402063) | 17262.18(15677.13 to 18930.3) | 1.26 (1.24 to 1.28) | 1.27 (1.25 to 1.28) | <0.001 |
| Paraguay | 257288(224735 to 293259) | 9875.78(8741.78 to 11112.72) | 800795(718616 to 890723) | 12573.51(11355.99 to 13893) | 0.82 (0.8 to 0.85) | 0.8 (0.77 to 0.82) | <0.001 |
| Peru | 1046697(902703 to 1211378) | 7162.28(6254.46 to 8188.45) | 3053945(2689922 to 3460639) | 8631.33(7631.8 to 9742.54) | 0.63 (0.59 to 0.67) | 0.6 (0.59 to 0.62) | <0.001 |
| Philippines | 5109220(4316927 to 6029948) | 12622.03(10881.74 to 14614.64) | 12859004(11056377 to 14906165) | 13372.3(11639.19 to 15323.04) | 0.17 (0.13 to 0.21) | 0.19 (0.16 to 0.22) | <0.001 |
| Poland | 4054510(3618113 to 4543938) | 9569.47(8522.43 to 10742.55) | 6576874(5937394 to 7260926) | 10736.05(9576.62 to 11993.63) | 0.33 (0.3 to 0.37) | 0.36 (0.31 to 0.42) | <0.001 |
| Portugal | 1018327(911053 to 1135165) | 8084.14(7190.38 to 9064.86) | 2072456(1876804 to 2278931) | 11162(9986 to 12412.66) | 1.03 (1.01 to 1.06) | 1.04 (1.02 to 1.06) | <0.001 |
| Puerto Rico | 425781(382602 to 471411) | 11776.41(10581.77 to 13039.06) | 863329(791271 to 944788) | 15819.2(14307.55 to 17516.04) | 0.97 (0.9 to 1.05) | 0.95 (0.94 to 0.97) | <0.001 |
| Qatar | 39083(32921 to 46123) | 14182.62(12603.62 to 15958.93) | 476129(413238 to 543434) | 20581.07(18596.41 to 22592.17) | 1.12 (1.08 to 1.16) | 1.2 (1.17 to 1.22) | <0.001 |
| Republic of Korea | 3910133(3433355 to 4446600) | 10882.17(9693.11 to 12198.35) | 12463511(11577191 to 13379543) | 15260.55(14044.64 to 16531.05) | 1.06 (0.96 to 1.16) | 1.12 (1.06 to 1.19) | <0.001 |
| Republic of Moldova | 535771(463329 to 615618) | 12101.03(10495.05 to 13864.15) | 757685(676359 to 844473) | 14338.49(12676.31 to 16136.23) | 0.53 (0.48 to 0.58) | 0.55 (0.5 to 0.6) | <0.001 |
| Romania | 2099911(1869920 to 2354620) | 7931.91(7040.8 to 8915.83) | 2866430(2634178 to 3117588) | 9103.51(8246.33 to 10051.76) | 0.3 (0.24 to 0.35) | 0.44 (0.42 to 0.47) | <0.001 |
| Russian Federation | 17677796(15119884 to 20566275) | 10335.65(8824.42 to 12046.78) | 24221033(21275579 to 27465940) | 11823.08(10249.38 to 13569.43) | 0.44 (0.43 to 0.45) | 0.44 (0.43 to 0.44) | <0.001 |
| Rwanda | 284583(237682 to 339327) | 7112.41(6046.97 to 8327.27) | 672188(564597 to 794724) | 7577.03(6491.98 to 8798.21) | 0.16 (0.13 to 0.19) | 0.2 (0.17 to 0.22) | <0.001 |
| Saint Kitts and Nevis | 4320(3889 to 4793) | 12003.87(10799.63 to 13337.47) | 11048(9953 to 12269) | 15129.49(13634.8 to 16790.94) | 0.71 (0.69 to 0.73) | 0.75 (0.73 to 0.77) | <0.001 |
| Saint Lucia | 13039(11680 to 14551) | 13547.69(12205.37 to 15033.9) | 38804(35484 to 42123) | 16716.04(15242.64 to 18202.14) | 0.67 (0.66 to 0.69) | 0.68 (0.67 to 0.69) | <0.001 |
| Saint Vincent and the Grenadines | 9797(8803 to 10882) | 12598.55(11392.76 to 13906.26) | 23279(21169 to 25459) | 16881.4(15311.95 to 18522.41) | 0.93 (0.91 to 0.94) | 0.95 (0.93 to 0.96) | <0.001 |
| Samoa | 14827(13186 to 16663) | 14160.02(12734.21 to 15748.8) | 35583(32212 to 39213) | 21366.11(19448.25 to 23426.33) | 1.37 (1.32 to 1.43) | 1.33 (1.32 to 1.34) | <0.001 |
| San Marino | 2295(2023 to 2588) | 7235.98(6335.21 to 8218.51) | 5366(4865 to 5916) | 9299.48(8310.33 to 10394.33) | 0.81 (0.78 to 0.85) | 0.81 (0.8 to 0.83) | <0.001 |
| Sao Tome and Principe | 7283(6347 to 8329) | 9912.03(8711.3 to 11244.41) | 19335(17153 to 21706) | 12683.72(11394.55 to 14068.8) | 0.81 (0.8 to 0.83) | 0.8 (0.79 to 0.81) | <0.001 |
| Saudi Arabia | 1169257(1004523 to 1357361) | 12954.96(11443.07 to 14641.74) | 5924719(5235939 to 6665825) | 17784.9(16050.2 to 19655.19) | 0.99 (0.98 to 1.01) | 1.02 (0.99 to 1.04) | <0.001 |
| Senegal | 411587(356499 to 474098) | 9792.86(8609.44 to 11113.98) | 1255810(1112448 to 1415209) | 12467.86(11203.51 to 13860.35) | 0.86 (0.84 to 0.89) | 0.81 (0.78 to 0.85) | <0.001 |
| Serbia | 982382(868565 to 1104507) | 8878.1(7830.95 to 10010.63) | 1536678(1387298 to 1699802) | 10899.39(9714.03 to 12203.01) | 0.64 (0.62 to 0.66) | 0.65 (0.64 to 0.67) | <0.001 |
| Seychelles | 7786(6633 to 9100) | 12763.12(10948.14 to 14824.18) | 22280(19892 to 24858) | 18560.57(16588.79 to 20689.76) | 1.24 (1.22 to 1.26) | 1.22 (1.21 to 1.23) | <0.001 |
| Sierra Leone | 237078(204147 to 274432) | 9259.88(8085.66 to 10575.48) | 622740(545953 to 708396) | 11555.71(10317.28 to 12924.17) | 0.74 (0.73 to 0.75) | 0.72 (0.72 to 0.73) | <0.001 |
| Singapore | 380260(331147 to 435182) | 13826.4(12243.73 to 15572.09) | 1245957(1120247 to 1378675) | 15347.82(13726.29 to 17078.44) | 0.25 (0.22 to 0.28) | 0.33 (0.3 to 0.36) | <0.001 |
| Slovakia | 486843(432034 to 545505) | 8407.58(7446.29 to 9440.03) | 775945(703085 to 854370) | 9285.59(8319.62 to 10340.46) | 0.31 (0.29 to 0.33) | 0.32 (0.31 to 0.34) | <0.001 |
| Slovenia | 200601(178015 to 225586) | 8485.37(7501.99 to 9575.05) | 335137(302268 to 369918) | 9295.7(8243.73 to 10436.7) | 0.24 (0.19 to 0.28) | 0.29 (0.28 to 0.3) | <0.001 |
| Solomon Islands | 20879(18227 to 23951) | 11108.48(9832.98 to 12558.98) | 72484(64985 to 80665) | 15263.32(13840.91 to 16797.05) | 1.03 (1 to 1.07) | 1.03 (1.01 to 1.05) | <0.001 |
| Somalia | 282192(236195 to 335649) | 6978.07(5982.84 to 8122.71) | 876321(745256 to 1027672) | 8213.53(7156.44 to 9398.43) | 0.54 (0.53 to 0.55) | 0.53 (0.52 to 0.53) | <0.001 |
| South Africa | 2772610(2421343 to 3170964) | 11013.64(9757.06 to 12420.05) | 6816366(6082268 to 7623007) | 13143.71(11827.65 to 14585.68) | 0.58 (0.57 to 0.59) | 0.57 (0.56 to 0.59) | <0.001 |
| South Sudan | 236140(196946 to 282341) | 6882.41(5840.79 to 8091.09) | 449486(381772 to 526892) | 8083.95(6997.96 to 9307.71) | 0.53 (0.52 to 0.54) | 0.52 (0.51 to 0.53) | <0.001 |
| Spain | 4361838(3974555 to 4784761) | 8663.57(7846.31 to 9562.5) | 8431664(7737304 to 9147517) | 10748.66(9737.48 to 11811.34) | 0.65 (0.63 to 0.68) | 0.69 (0.65 to 0.73) | <0.001 |
| Sri Lanka | 1743703(1482654 to 2040915) | 13160.98(11392.3 to 15144.44) | 4599038(4125286 to 5108325) | 17663.55(15781.62 to 19707.04) | 1 (0.97 to 1.03) | 0.95 (0.94 to 0.97) | <0.001 |
| Sudan | 1328931(1130408 to 1559874) | 11221.25(9729.96 to 12920.71) | 4160552(3632763 to 4751909) | 15082.44(13433.69 to 16872.34) | 0.94 (0.92 to 0.96) | 0.95 (0.92 to 0.99) | <0.001 |
| Suriname | 32951(29253 to 36919) | 11202.44(10026.03 to 12449.46) | 106196(96967 to 115859) | 16600.24(15146.42 to 18122.34) | 1.37 (1.34 to 1.41) | 1.28 (1.25 to 1.3) | <0.001 |
| Sweden | 1101668(969105 to 1243122) | 8892.71(7720.56 to 10164.79) | 1724315(1542361 to 1917942) | 10481.15(9214.15 to 11846.68) | 0.56 (0.54 to 0.58) | 0.55 (0.51 to 0.59) | <0.001 |
| Switzerland | 877496(788898 to 974386) | 9310.86(8304.47 to 10422.26) | 1728553(1577481 to 1883101) | 11872.66(10701.49 to 13103.28) | 0.72 (0.69 to 0.76) | 0.79 (0.77 to 0.81) | <0.001 |
| Syrian Arab Republic | 850239(732589 to 986582) | 12072.92(10590.02 to 13750.6) | 2177994(1946904 to 2429788) | 15505.4(13878.67 to 17275.27) | 0.79 (0.78 to 0.8) | 0.81 (0.8 to 0.81) | <0.001 |
| Taiwan (Province of China) | 1738356(1544216 to 1959368) | 9746.78(8743.65 to 10880.88) | 4180149(3812844 to 4566213) | 11234.99(10147.83 to 12398.48) | 0.41 (0.38 to 0.44) | 0.46 (0.41 to 0.5) | <0.001 |
| Tajikistan | 357196(308694 to 413998) | 10849.17(9508.84 to 12393.57) | 936579(823725 to 1063398) | 12593.53(11234.87 to 14087.07) | 0.49 (0.47 to 0.5) | 0.48 (0.46 to 0.5) | <0.001 |
| Thailand | 5360050(4515421 to 6328593) | 11909.7(10211.97 to 13830.99) | 13749781(12235434 to 15387763) | 14208.22(12497.77 to 16082.14) | 0.55 (0.54 to 0.57) | 0.57 (0.57 to 0.58) | <0.001 |
| Timor-Leste | 49441(40444 to 59758) | 10894.68(9183.36 to 12811.14) | 141705(121597 to 164176) | 14344.79(12479.51 to 16398.89) | 0.91 (0.88 to 0.94) | 0.9 (0.89 to 0.91) | <0.001 |
| Togo | 157538(133963 to 184925) | 8668.13(7541.96 to 9941.71) | 532847(463482 to 609230) | 10020.7(8869.09 to 11259.34) | 0.44 (0.42 to 0.45) | 0.47 (0.46 to 0.48) | <0.001 |
| Tokelau | 200(179 to 223) | 15220.18(13647.54 to 16960.1) | 323(295 to 352) | 22785.11(20797.92 to 24907.4) | 1.28 (1.23 to 1.34) | 1.31 (1.29 to 1.32) | <0.001 |
| Tonga | 8879(7858 to 9969) | 13809.79(12329.15 to 15372.01) | 17085(15537 to 18745) | 19827.46(18086.62 to 21681.83) | 1.19 (1.18 to 1.2) | 1.17 (1.16 to 1.18) | <0.001 |
| Trinidad and Tobago | 126836(114527 to 140269) | 13579.03(12360.1 to 14903.06) | 328138(298674 to 357099) | 17921.42(16234.6 to 19602.38) | 0.85 (0.83 to 0.88) | 0.9 (0.87 to 0.92) | <0.001 |
| Tunisia | 673404(577657 to 782360) | 11274.76(9806.71 to 12936.02) | 2180312(1954528 to 2430928) | 16211.58(14517.74 to 18095.49) | 1.15 (1.12 to 1.19) | 1.18 (1.15 to 1.21) | <0.001 |
| Turkey | 4891087(4159166 to 5754727) | 11541.75(9959.79 to 13380.62) | 13505847(11963950 to 15197894) | 14294.11(12643.93 to 16107.9) | 0.72 (0.66 to 0.78) | 0.7 (0.67 to 0.72) | <0.001 |
| Turkmenistan | 275999(238380 to 318349) | 11711.69(10272.85 to 13301.63) | 613355(545502 to 688881) | 13426.54(12011.23 to 14983.25) | 0.45 (0.45 to 0.46) | 0.44 (0.43 to 0.46) | <0.001 |
| Tuvalu | 919(810 to 1035) | 12309.22(10931.54 to 13772.95) | 1969(1782 to 2164) | 17543.81(15927.5 to 19217.01) | 1.12 (1.1 to 1.14) | 1.14 (1.13 to 1.16) | <0.001 |
| Uganda | 661986(550889 to 790824) | 7316.27(6214.51 to 8574.43) | 1913553(1618197 to 2251624) | 8359.13(7241.66 to 9620.79) | 0.41 (0.39 to 0.42) | 0.43 (0.4 to 0.46) | <0.001 |
| Ukraine | 7001159(6009016 to 8109796) | 10913.85(9301.97 to 12721.29) | 8011746(7056449 to 9070859) | 12295.6(10681.74 to 14099.85) | 0.36 (0.34 to 0.37) | 0.38 (0.37 to 0.39) | <0.001 |
| United Arab Emirates | 152491(127298 to 181540) | 13363.64(11745.82 to 15207.92) | 1612539(1410649 to 1825414) | 16452.38(14728.05 to 18311.08) | 0.61 (0.59 to 0.63) | 0.67 (0.65 to 0.7) | <0.001 |
| United Kingdom | 6754212(6081423 to 7497409) | 8400.89(7506.37 to 9394.93) | 12248345(11259655 to 13315136) | 11992.38(10904.14 to 13172.1) | 0.97 (0.87 to 1.07) | 1.13 (1.02 to 1.24) | <0.001 |
| United Republic of Tanzania | 952366(789129 to 1141293) | 6450.41(5439.88 to 7603.25) | 2924272(2467422 to 3453399) | 8017.04(6896.21 to 9298.57) | 0.74 (0.72 to 0.77) | 0.69 (0.67 to 0.71) | <0.001 |
| United States of America | 30505185(27253213 to 34130767) | 10128.62(9019.07 to 11376.22) | 71668418(66792223 to 77125227) | 14292.98(13184.37 to 15542.77) | 1.25 (1.21 to 1.29) | 1.13 (1.08 to 1.18) | <0.001 |
| United States Virgin Islands | 11548(10278 to 12911) | 12174.66(10898.46 to 13532.2) | 25855(23783 to 28057) | 17652.28(16014.1 to 19391.36) | 1.27 (1.23 to 1.32) | 1.2 (1.18 to 1.23) | <0.001 |
| Uruguay | 285817(250675 to 324633) | 7939.77(6914.84 to 9080.57) | 509670(458905 to 563088) | 10733.35(9559.93 to 11990.24) | 1.07 (1.02 to 1.11) | 0.98 (0.92 to 1.03) | <0.001 |
| Uzbekistan | 1621762(1423340 to 1850199) | 11786.52(10464.14 to 13280.76) | 4533080(4068269 to 5037335) | 14806.93(13375.63 to 16343.38) | 0.82 (0.79 to 0.84) | 0.74 (0.73 to 0.76) | <0.001 |
| Vanuatu | 10686(9253 to 12319) | 12133.21(10693.5 to 13746.06) | 40314(36226 to 44691) | 17677.82(16041.13 to 19419.88) | 1.2 (1.18 to 1.22) | 1.22 (1.2 to 1.23) | <0.001 |
| Venezuela (Bolivarian Republic of) | 1356135(1188647 to 1543725) | 11132.53(9913.99 to 12479.09) | 3991252(3622709 to 4391297) | 13361.4(12109.5 to 14724.95) | 0.61 (0.57 to 0.65) | 0.6 (0.55 to 0.65) | <0.001 |
| Viet Nam | 5088771(4201310 to 6088904) | 10570.26(8856.39 to 12483.07) | 13003586(11085864 to 15131376) | 12373.58(10605.84 to 14334.65) | 0.58 (0.53 to 0.63) | 0.5 (0.46 to 0.54) | <0.001 |
| Yemen | 685109(578502 to 809729) | 10203.58(8802.56 to 11792.71) | 2605843(2253644 to 3015948) | 12911.57(11425.5 to 14612.66) | 0.67 (0.6 to 0.74) | 0.77 (0.68 to 0.85) | <0.001 |
| Zambia | 315478(267758 to 369428) | 7607.18(6576.12 to 8752.85) | 1086997(941832 to 1252962) | 9674.74(8541.68 to 10943.35) | 0.81 (0.79 to 0.83) | 0.78 (0.75 to 0.8) | <0.001 |
| Zimbabwe | 518264(445278 to 602987) | 9804.84(8606.52 to 11165.35) | 1090616(951808 to 1243082) | 11802.15(10514.13 to 13187.52) | 0.65 (0.64 to 0.67) | 0.61 (0.59 to 0.62) | <0.001 |

*ASR: age-standardized rate; EAPC: estimated annual percentage change; AAPC: average annual percent change; SDI: sociodemographic index; UI: uncertainty interval; CI: confidence interval.*

**Table S5. The case number and ASR of incidence of diabetes and kidney diseases in 1990 and 2021, and its temporal trends from 1990 to 2021 in 204 countries and territories.**

| **location** | **1990** | | **2021** | | **EAPC (95% CI)** | **AAPC (95% CI)** | **P value** |
| --- | --- | --- | --- | --- | --- | --- | --- |
|  | **Case number (95% UI)** | **ASR (95% UI)** | **Case number (95% UI)** | **ASR (95% UI)** |  |  |  |
| Afghanistan | 41177(31796 to 51584) | 541.43(414.84 to 681.86) | 186368(142766 to 233107) | 1038.3(791.95 to 1305.27) | 2.12 (2.05 to 2.19) | 2.12 (2.08 to 2.16) | <0.001 |
| Albania | 6616(5164 to 8230) | 264.39(205.95 to 329.18) | 15560(12124 to 19388) | 402.81(313.69 to 502.29) | 1.52 (1.41 to 1.63) | 1.37 (1.3 to 1.43) | <0.001 |
| Algeria | 71417(55214 to 89775) | 474.55(365.86 to 598.41) | 368556(290072 to 452859) | 898.6(702.65 to 1110.49) | 2.05 (2.01 to 2.08) | 2.08 (2.05 to 2.11) | <0.001 |
| American Samoa | 227(174 to 285) | 677.82(523.39 to 849.93) | 616(465 to 783) | 1189.31(891.63 to 1519.46) | 1.72 (1.61 to 1.83) | 1.83 (1.79 to 1.86) | <0.001 |
| Andorra | 198(146 to 258) | 342.3(252.66 to 446.74) | 612(469 to 775) | 453.81(345.58 to 577.23) | 0.97 (0.94 to 1) | 0.92 (0.9 to 0.94) | <0.001 |
| Angola | 16913(13185 to 20987) | 298.84(235.75 to 366.79) | 84688(65697 to 105592) | 443.47(347.57 to 547.57) | 1.31 (1.26 to 1.36) | 1.28 (1.26 to 1.29) | <0.001 |
| Antigua and Barbuda | 292(229 to 363) | 567.94(447.15 to 701.11) | 975(770 to 1199) | 877.89(689.83 to 1085.43) | 1.43 (1.38 to 1.49) | 1.41 (1.38 to 1.45) | <0.001 |
| Argentina | 125186(97720 to 156065) | 387.33(301.88 to 483.51) | 304250(240864 to 372131) | 570.49(453.01 to 696.44) | 1.28 (1.21 to 1.35) | 1.26 (1.22 to 1.3) | <0.001 |
| Armenia | 8494(6610 to 10550) | 268.36(207.74 to 334.65) | 17257(13361 to 21561) | 446.45(345.41 to 557.4) | 1.7 (1.61 to 1.78) | 1.66 (1.6 to 1.73) | <0.001 |
| Australia | 73501(58779 to 90807) | 375.43(299.92 to 463.97) | 203284(156908 to 252701) | 500.15(387.92 to 620.21) | 0.93 (0.88 to 0.98) | 0.93 (0.9 to 0.97) | <0.001 |
| Austria | 36084(26401 to 47207) | 312.33(230.23 to 406.71) | 69858(52620 to 90170) | 436.03(331.99 to 556.91) | 1.16 (1.09 to 1.22) | 1.09 (1.07 to 1.11) | <0.001 |
| Azerbaijan | 14183(10920 to 17663) | 230(176.34 to 287.17) | 53392(41964 to 65469) | 453.98(354.1 to 560.29) | 2.42 (2.35 to 2.49) | 2.22 (2.2 to 2.25) | <0.001 |
| Bahamas | 953(754 to 1168) | 495.02(392.6 to 604.82) | 3393(2685 to 4165) | 765.71(602.72 to 944.35) | 1.48 (1.42 to 1.53) | 1.42 (1.36 to 1.47) | <0.001 |
| Bahrain | 1909(1493 to 2377) | 704.54(550.72 to 874.88) | 18606(14868 to 22688) | 1232.04(970 to 1514.34) | 1.78 (1.69 to 1.88) | 1.82 (1.78 to 1.86) | <0.001 |
| Bangladesh | 172614(135606 to 214020) | 260.11(207.17 to 319.08) | 698641(545746 to 867110) | 445.24(348.45 to 551.91) | 1.74 (1.68 to 1.79) | 1.74 (1.7 to 1.78) | <0.001 |
| Barbados | 1417(1118 to 1732) | 552.33(438.76 to 671.56) | 3551(2797 to 4359) | 810.91(639.48 to 996.93) | 1.18 (1.15 to 1.22) | 1.25 (1.21 to 1.29) | <0.001 |
| Belarus | 22446(17344 to 27932) | 185.9(143.32 to 232.28) | 38850(30209 to 48366) | 291.46(226.92 to 362.3) | 1.41 (1.31 to 1.5) | 1.45 (1.42 to 1.49) | <0.001 |
| Belgium | 53892(39752 to 69588) | 368.75(273.07 to 474.45) | 92405(71040 to 117866) | 479.71(369.1 to 609.97) | 0.88 (0.86 to 0.9) | 0.85 (0.83 to 0.87) | <0.001 |
| Belize | 514(402 to 636) | 452.61(354.37 to 557.65) | 2776(2217 to 3412) | 749.19(596.73 to 921.18) | 1.66 (1.61 to 1.71) | 1.63 (1.61 to 1.66) | <0.001 |
| Benin | 8314(6491 to 10321) | 309.92(243.44 to 382.87) | 39157(30498 to 48949) | 493.55(388.18 to 612.73) | 1.44 (1.37 to 1.51) | 1.52 (1.46 to 1.58) | <0.001 |
| Bermuda | 250(198 to 309) | 390.12(307.94 to 482.49) | 690(528 to 862) | 631(487.16 to 786.17) | 1.64 (1.55 to 1.73) | 1.59 (1.55 to 1.63) | <0.001 |
| Bhutan | 1021(791 to 1281) | 272.88(212.01 to 341.31) | 3087(2405 to 3836) | 436.59(340.12 to 542.81) | 1.64 (1.59 to 1.69) | 1.53 (1.5 to 1.57) | <0.001 |
| Bolivia (Plurinational State of) | 14106(11147 to 17347) | 361.48(285.01 to 444.42) | 61608(48532 to 76014) | 607.65(477.83 to 750.69) | 1.79 (1.76 to 1.83) | 1.69 (1.66 to 1.73) | <0.001 |
| Bosnia and Herzegovina | 14676(11712 to 17760) | 316.96(251.46 to 386.07) | 32763(25959 to 40141) | 617.8(489.08 to 758.11) | 2.42 (2.25 to 2.59) | 2.19 (2.12 to 2.26) | <0.001 |
| Botswana | 2351(1863 to 2897) | 340.11(272.52 to 415.36) | 9471(7610 to 11547) | 526.1(423.55 to 641.94) | 1.43 (1.37 to 1.49) | 1.42 (1.39 to 1.44) | <0.001 |
| Brazil | 455711(357350 to 564610) | 423.81(331.61 to 525.44) | 1424553(1137225 to 1741527) | 567.22(451.91 to 694.74) | 0.98 (0.96 to 1) | 0.94 (0.87 to 1.02) | <0.001 |
| Brunei Darussalam | 930(735 to 1153) | 682.69(539.16 to 845.18) | 4638(3536 to 5862) | 1026.73(780 to 1302.05) | 1.18 (1.13 to 1.22) | 1.33 (1.31 to 1.34) | <0.001 |
| Bulgaria | 37106(28552 to 45882) | 319.3(245.7 to 396.15) | 59760(47232 to 73403) | 540.7(430.51 to 659.94) | 1.75 (1.67 to 1.84) | 1.72 (1.68 to 1.76) | <0.001 |
| Burkina Faso | 14708(11673 to 18076) | 268.5(214.44 to 328.49) | 52633(41797 to 64994) | 402.29(320.79 to 493.82) | 1.35 (1.27 to 1.42) | 1.32 (1.27 to 1.37) | <0.001 |
| Burundi | 7471(6018 to 9111) | 240.78(195 to 292.63) | 20763(16443 to 25500) | 292.73(233.75 to 356.49) | 0.57 (0.52 to 0.62) | 0.62 (0.6 to 0.65) | <0.001 |
| Cabo Verde | 703(546 to 875) | 275.13(214.11 to 341.34) | 2634(2062 to 3275) | 518.98(407.94 to 643.2) | 2.22 (2.16 to 2.29) | 2.07 (2.04 to 2.1) | <0.001 |
| Cambodia | 17367(13855 to 21216) | 279.55(223.61 to 340.17) | 71430(57449 to 86380) | 496.57(398.81 to 601) | 1.91 (1.83 to 1.99) | 1.88 (1.84 to 1.92) | <0.001 |
| Cameroon | 19975(15404 to 24928) | 336.61(260.66 to 419.08) | 93082(73452 to 115113) | 503.87(398.75 to 620.4) | 1.32 (1.28 to 1.36) | 1.32 (1.27 to 1.37) | <0.001 |
| Canada | 129526(100813 to 159573) | 407.07(317.79 to 501.16) | 376402(289835 to 472684) | 620.5(481.58 to 773.86) | 1.41 (1.36 to 1.46) | 1.37 (1.33 to 1.41) | <0.001 |
| Central African Republic | 5136(3992 to 6468) | 318.47(250.06 to 397.73) | 17264(13109 to 21871) | 467.79(358.83 to 587.57) | 1.26 (1.24 to 1.28) | 1.24 (1.21 to 1.26) | <0.001 |
| Chad | 9847(7718 to 12247) | 272.13(213.51 to 337.9) | 37147(29179 to 46248) | 405.31(321.21 to 501.45) | 1.27 (1.24 to 1.3) | 1.29 (1.26 to 1.31) | <0.001 |
| Chile | 40820(31259 to 51288) | 383.31(292.51 to 483.24) | 166021(128985 to 208591) | 678.84(527.21 to 853.79) | 2.01 (1.89 to 2.14) | 1.87 (1.83 to 1.91) | <0.001 |
| China | 3436313(2559240 to 4431724) | 340.2(253.5 to 438.29) | 7441095(5714152 to 9352808) | 415.44(317.87 to 523.73) | 0.61 (0.57 to 0.66) | 0.63 (0.58 to 0.69) | <0.001 |
| Colombia | 99771(77440 to 124308) | 448.02(347.38 to 557.98) | 331911(261757 to 408005) | 608.98(480.04 to 748.75) | 0.94 (0.9 to 0.97) | 1.02 (0.98 to 1.06) | <0.001 |
| Comoros | 713(565 to 876) | 276.81(221.12 to 338.23) | 2195(1727 to 2717) | 376.91(298.64 to 463.55) | 0.97 (0.94 to 1.01) | 0.99 (0.97 to 1.02) | <0.001 |
| Congo | 4207(3316 to 5194) | 300.76(239.61 to 368.12) | 17407(13731 to 21628) | 455.17(361.96 to 561.44) | 1.36 (1.31 to 1.42) | 1.34 (1.3 to 1.39) | <0.001 |
| Cook Islands | 107(82 to 136) | 701.66(537.49 to 886.5) | 233(177 to 290) | 1105.44(841.28 to 1380.79) | 1.32 (1.23 to 1.41) | 1.47 (1.44 to 1.5) | <0.001 |
| Costa Rica | 13069(10578 to 15650) | 652.05(532.32 to 774.05) | 43923(36288 to 52117) | 807.6(665.26 to 961.02) | 0.7 (0.67 to 0.73) | 0.69 (0.6 to 0.78) | <0.001 |
| Coted'Ivoire | 19671(15252 to 24608) | 319.02(249.13 to 395.11) | 79788(62356 to 99095) | 476.5(374.61 to 588.2) | 1.28 (1.26 to 1.3) | 1.31 (1.26 to 1.36) | <0.001 |
| Croatia | 21521(16959 to 26504) | 349.52(274.17 to 432.91) | 39684(31437 to 49189) | 550.09(437.75 to 678.26) | 1.58 (1.46 to 1.69) | 1.48 (1.42 to 1.55) | <0.001 |
| Cuba | 35834(27944 to 44159) | 339.22(264.65 to 417.88) | 95333(75206 to 117666) | 567.79(447.45 to 702.57) | 1.69 (1.66 to 1.72) | 1.68 (1.64 to 1.71) | <0.001 |
| Cyprus | 4834(3832 to 5973) | 574.57(453.73 to 711.32) | 12013(9291 to 15069) | 618.44(477.42 to 775.75) | 0.15 (0.06 to 0.25) | 0.24 (0.21 to 0.27) | <0.001 |
| Czechia | 40806(32039 to 50624) | 319.81(251.31 to 396.89) | 91905(71614 to 113567) | 527.91(414.31 to 648.19) | 1.72 (1.65 to 1.79) | 1.63 (1.58 to 1.68) | <0.001 |
| Democratic People's Republic of Korea | 54232(42192 to 67590) | 292.61(225.85 to 367.03) | 144410(112929 to 178491) | 457.01(355.94 to 567.04) | 1.45 (1.42 to 1.49) | 1.45 (1.44 to 1.46) | <0.001 |
| Democratic Republic of the Congo | 55838(44233 to 69033) | 264.03(211.14 to 323.6) | 209123(164395 to 258500) | 380.28(301.73 to 466.99) | 1.17 (1.1 to 1.25) | 1.19 (1.15 to 1.22) | <0.001 |
| Denmark | 23119(17062 to 30174) | 304.24(226.11 to 395) | 44920(34780 to 56348) | 445.81(346.87 to 557.34) | 1.33 (1.3 to 1.36) | 1.24 (1.22 to 1.26) | <0.001 |
| Djibouti | 450(358 to 553) | 227.25(183.45 to 276.65) | 2723(2177 to 3324) | 328.14(264.17 to 397.81) | 1.17 (1.11 to 1.24) | 1.18 (1.17 to 1.2) | <0.001 |
| Dominica | 348(271 to 432) | 583.73(457.1 to 722.84) | 703(553 to 868) | 866.83(679.14 to 1073.47) | 1.25 (1.2 to 1.29) | 1.28 (1.23 to 1.33) | <0.001 |
| Dominican Republic | 17232(13592 to 21246) | 356.29(281.72 to 438.11) | 73129(57490 to 90724) | 679.85(534.51 to 843.03) | 2.2 (2.14 to 2.26) | 2.11 (2.08 to 2.14) | <0.001 |
| Ecuador | 23070(17781 to 28858) | 363.7(280.06 to 455.03) | 126649(99071 to 156877) | 741.96(579.9 to 919.85) | 2.46 (2.35 to 2.58) | 2.33 (2.28 to 2.38) | <0.001 |
| Egypt | 132203(100853 to 167041) | 396.26(297.63 to 505.54) | 694538(541795 to 862856) | 862.31(666.16 to 1078.94) | 2.53 (2.46 to 2.59) | 2.53 (2.44 to 2.63) | <0.001 |
| El Salvador | 14317(10979 to 17973) | 402.43(307.95 to 505.46) | 45670(35953 to 56440) | 740.92(584.19 to 914.2) | 2.16 (2.06 to 2.27) | 1.99 (1.91 to 2.07) | <0.001 |
| Equatorial Guinea | 706(558 to 875) | 281.23(223.82 to 345.81) | 4297(3327 to 5417) | 508.94(401.94 to 629.86) | 2.16 (2.06 to 2.26) | 1.94 (1.9 to 1.98) | <0.001 |
| Eritrea | 4237(3339 to 5258) | 251.55(201.05 to 309.05) | 14486(11251 to 18204) | 352.12(277.63 to 436.81) | 1.06 (1 to 1.11) | 1.08 (1.07 to 1.1) | <0.001 |
| Estonia | 4270(3277 to 5351) | 228.33(175.33 to 286.55) | 8850(6693 to 11255) | 448.89(344.55 to 564.48) | 2.31 (2.26 to 2.36) | 2.2 (2.18 to 2.23) | <0.001 |
| Eswatini | 1597(1259 to 1980) | 424.45(338.5 to 520.84) | 4734(3816 to 5738) | 655.87(531.87 to 792.51) | 1.44 (1.38 to 1.49) | 1.42 (1.38 to 1.45) | <0.001 |
| Ethiopia | 76577(61098 to 94231) | 278.49(222.79 to 342.09) | 194077(152666 to 239721) | 315.08(250.13 to 385.56) | 0.26 (0.16 to 0.36) | 0.39 (0.37 to 0.42) | <0.001 |
| Fiji | 3588(2853 to 4397) | 720.58(577.33 to 877.1) | 10080(8114 to 12255) | 1108.78(889.06 to 1351.91) | 1.25 (1.17 to 1.33) | 1.39 (1.36 to 1.43) | <0.001 |
| Finland | 23075(17755 to 28964) | 353.82(273.7 to 443) | 47435(36659 to 59439) | 520.82(404.62 to 649.99) | 1.18 (1.14 to 1.23) | 1.25 (1.21 to 1.29) | <0.001 |
| France | 250849(189035 to 322147) | 311.8(236.51 to 397.5) | 474252(365649 to 600625) | 406.68(315.23 to 512.72) | 0.89 (0.84 to 0.94) | 0.87 (0.84 to 0.89) | <0.001 |
| Gabon | 2250(1773 to 2781) | 337.89(267.65 to 415.95) | 7475(5899 to 9273) | 554.23(439.2 to 683.86) | 1.64 (1.61 to 1.68) | 1.6 (1.56 to 1.64) | <0.001 |
| Gambia | 1430(1119 to 1786) | 281(221.14 to 348.58) | 6256(4915 to 7757) | 446.22(354.69 to 548.85) | 1.53 (1.49 to 1.58) | 1.51 (1.49 to 1.54) | <0.001 |
| Georgia | 14383(10968 to 17965) | 236.58(180.24 to 296.32) | 21500(16628 to 26696) | 460.43(357.7 to 571.43) | 2.34 (2.29 to 2.38) | 2.18 (2.13 to 2.22) | <0.001 |
| Germany | 427053(321706 to 546033) | 350.48(264.93 to 447.42) | 798486(630680 to 993321) | 503.76(399.08 to 623.22) | 1.15 (1.09 to 1.22) | 1.19 (1.16 to 1.21) | <0.001 |
| Ghana | 23262(18215 to 28901) | 271.81(214.57 to 335.98) | 102495(80723 to 126626) | 445.69(353.02 to 547.49) | 1.63 (1.55 to 1.71) | 1.6 (1.52 to 1.69) | <0.001 |
| Greece | 62911(45506 to 83004) | 430.07(311.93 to 566.23) | 94668(71068 to 123107) | 506.63(381.74 to 654.76) | 0.54 (0.5 to 0.58) | 0.52 (0.49 to 0.55) | <0.001 |
| Greenland | 93(71 to 119) | 272.73(204.55 to 350.96) | 305(242 to 372) | 440.77(343.66 to 546.41) | 1.64 (1.5 to 1.78) | 1.57 (1.54 to 1.59) | <0.001 |
| Grenada | 386(304 to 477) | 572.2(454.49 to 704.37) | 1111(878 to 1373) | 929.38(730.52 to 1152.89) | 1.54 (1.47 to 1.61) | 1.58 (1.55 to 1.61) | <0.001 |
| Guam | 448(350 to 559) | 427.98(333.67 to 534) | 1207(937 to 1500) | 643.93(500.78 to 799.97) | 1.27 (1.25 to 1.29) | 1.33 (1.31 to 1.35) | <0.001 |
| Guatemala | 20819(16107 to 26042) | 437.54(335.48 to 549.41) | 109047(85243 to 135086) | 846.6(660.62 to 1049.25) | 2.32 (2.16 to 2.48) | 2.16 (2.07 to 2.26) | <0.001 |
| Guinea | 10972(8583 to 13498) | 274.85(215.08 to 338.05) | 31808(25232 to 39081) | 404.99(323.37 to 494.12) | 1.15 (1.12 to 1.19) | 1.25 (1.21 to 1.28) | <0.001 |
| Guinea-Bissau | 1839(1429 to 2284) | 327.02(256.24 to 403.33) | 5633(4400 to 7030) | 473.17(374.18 to 581.53) | 1.2 (1.17 to 1.22) | 1.2 (1.15 to 1.26) | <0.001 |
| Guyana | 3412(2616 to 4273) | 650.7(500.48 to 810.15) | 7688(6004 to 9473) | 1025.31(798.67 to 1265.94) | 1.44 (1.38 to 1.49) | 1.47 (1.43 to 1.51) | <0.001 |
| Haiti | 22758(17720 to 28414) | 529.57(412.12 to 662.24) | 78449(60581 to 98147) | 767.39(592.73 to 959.38) | 1.18 (1.17 to 1.2) | 1.2 (1.18 to 1.22) | <0.001 |
| Honduras | 12446(9634 to 15694) | 454.99(351.73 to 571.93) | 57302(44754 to 71413) | 721.77(564.24 to 899.36) | 1.6 (1.55 to 1.65) | 1.51 (1.49 to 1.54) | <0.001 |
| Hungary | 42137(33402 to 51796) | 312.61(247.86 to 385.27) | 82231(64810 to 101689) | 519.13(411.82 to 636.99) | 1.68 (1.5 to 1.86) | 1.65 (1.58 to 1.72) | <0.001 |
| Iceland | 828(621 to 1061) | 294.21(221.56 to 376.02) | 2080(1604 to 2614) | 416.94(320.49 to 524.81) | 1.13 (1.07 to 1.19) | 1.14 (1.1 to 1.19) | <0.001 |
| India | 1921606(1483275 to 2413332) | 318.61(246.14 to 399.47) | 6082341(4700747 to 7627604) | 448.23(346.32 to 561.87) | 0.98 (0.93 to 1.03) | 1.1 (1.04 to 1.15) | <0.001 |
| Indonesia | 379802(299897 to 471766) | 295.88(233.25 to 367.25) | 1307946(1042145 to 1604804) | 472.08(375.05 to 580.08) | 1.49 (1.4 to 1.58) | 1.51 (1.47 to 1.55) | <0.001 |
| Iran (Islamic Republic of) | 152700(116073 to 194013) | 464.74(349.49 to 594.11) | 676414(534674 to 832249) | 774.78(609.21 to 956.49) | 1.69 (1.65 to 1.73) | 1.66 (1.6 to 1.72) | <0.001 |
| Iraq | 67214(51152 to 84660) | 644.08(489.04 to 811.52) | 380693(294838 to 476221) | 1146.37(884.34 to 1437.67) | 1.91 (1.86 to 1.96) | 1.88 (1.84 to 1.92) | <0.001 |
| Ireland | 16035(12447 to 20079) | 399.37(309.6 to 500.49) | 31698(25433 to 38340) | 455.33(364.13 to 553.22) | 0.56 (0.49 to 0.63) | 0.43 (0.42 to 0.44) | <0.001 |
| Israel | 22460(16730 to 28793) | 465.73(348.72 to 594.54) | 66510(50572 to 83632) | 590(450.8 to 737.85) | 0.83 (0.68 to 0.97) | 0.77 (0.72 to 0.82) | <0.001 |
| Italy | 326364(241797 to 422314) | 397.21(294.28 to 514.57) | 538237(410770 to 678583) | 463.16(350.9 to 589.72) | 0.49 (0.44 to 0.54) | 0.49 (0.46 to 0.52) | <0.001 |
| Jamaica | 8910(7075 to 10910) | 497.44(397.26 to 605.94) | 22486(18018 to 27359) | 733.86(588.03 to 892.76) | 1.26 (1.19 to 1.32) | 1.28 (1.17 to 1.38) | <0.001 |
| Japan | 791611(616662 to 984011) | 480.12(372.88 to 598.82) | 1522374(1177895 to 1904699) | 581.19(453.11 to 725.85) | 0.5 (0.43 to 0.57) | 0.63 (0.61 to 0.66) | <0.001 |
| Jordan | 12759(10111 to 15606) | 682.27(538.92 to 835.68) | 113837(90473 to 139155) | 1115(880.68 to 1369.92) | 1.65 (1.6 to 1.7) | 1.62 (1.57 to 1.67) | <0.001 |
| Kazakhstan | 37868(29448 to 47292) | 255.99(198.8 to 320.09) | 96585(75735 to 118306) | 488.93(381.87 to 601.94) | 2.26 (2.19 to 2.33) | 2.11 (2.08 to 2.14) | <0.001 |
| Kenya | 23357(18584 to 28623) | 206.78(165.79 to 251.72) | 77462(61727 to 94712) | 265.1(212.71 to 322.49) | 0.67 (0.57 to 0.77) | 0.8 (0.76 to 0.83) | <0.001 |
| Kiribati | 314(246 to 389) | 593.55(467.81 to 732.24) | 947(743 to 1175) | 917.83(720.58 to 1138.91) | 1.4 (1.31 to 1.48) | 1.42 (1.39 to 1.45) | <0.001 |
| Kuwait | 6920(5349 to 8712) | 674.78(521.88 to 847.2) | 53854(41776 to 67217) | 1117.79(860.67 to 1402.18) | 1.78 (1.73 to 1.82) | 1.66 (1.63 to 1.68) | <0.001 |
| Kyrgyzstan | 8040(6243 to 9928) | 216.12(168.26 to 266.15) | 22013(17303 to 27015) | 354.97(277.84 to 437.18) | 1.66 (1.61 to 1.7) | 1.62 (1.58 to 1.66) | <0.001 |
| Lao People's Democratic Republic | 9713(7680 to 11984) | 363.02(286.12 to 448.28) | 34112(27450 to 41139) | 597.68(481.24 to 720.81) | 1.62 (1.57 to 1.67) | 1.63 (1.61 to 1.65) | <0.001 |
| Latvia | 6460(4959 to 8100) | 201.13(154.25 to 252.82) | 11005(8411 to 13835) | 392.32(302.64 to 488.72) | 2.18 (2.15 to 2.21) | 2.17 (2.12 to 2.22) | <0.001 |
| Lebanon | 13238(10220 to 16603) | 545.91(419.71 to 687.08) | 56580(43653 to 70990) | 975.47(755.14 to 1220.6) | 1.92 (1.88 to 1.97) | 1.9 (1.87 to 1.94) | <0.001 |
| Lesotho | 2928(2328 to 3585) | 304.63(243.34 to 371.56) | 6900(5605 to 8305) | 531.7(433.09 to 638.96) | 1.88 (1.77 to 1.99) | 1.81 (1.77 to 1.85) | <0.001 |
| Liberia | 4361(3412 to 5413) | 292.51(230.17 to 361.7) | 15427(11967 to 19404) | 464.95(365.39 to 577.82) | 1.62 (1.58 to 1.66) | 1.52 (1.49 to 1.56) | <0.001 |
| Libya | 11228(8558 to 14205) | 471.39(357.27 to 597.58) | 63471(49114 to 79172) | 930.78(713.48 to 1167) | 2.19 (2.11 to 2.27) | 2.23 (2.17 to 2.28) | <0.001 |
| Lithuania | 8652(6660 to 10863) | 205.49(158.11 to 258.4) | 13921(10853 to 17268) | 340.56(266.61 to 420.73) | 1.63 (1.57 to 1.69) | 1.64 (1.57 to 1.71) | <0.001 |
| Luxembourg | 1963(1478 to 2534) | 370.55(279.44 to 478.18) | 4583(3498 to 5836) | 485.53(370.08 to 618.98) | 0.94 (0.88 to 0.99) | 0.89 (0.85 to 0.92) | <0.001 |
| Madagascar | 14129(11286 to 17177) | 209.6(169.13 to 253.19) | 44935(35671 to 55174) | 275.72(220.63 to 335.3) | 0.86 (0.81 to 0.91) | 0.89 (0.87 to 0.9) | <0.001 |
| Malawi | 11507(9297 to 13957) | 225.92(184.26 to 272.61) | 28027(22465 to 33945) | 279.95(225.24 to 337.81) | 0.65 (0.59 to 0.71) | 0.69 (0.68 to 0.7) | <0.001 |
| Malaysia | 50111(39380 to 61912) | 434.93(344.23 to 534.09) | 213175(170633 to 259664) | 683.1(546.17 to 832.75) | 1.46 (1.43 to 1.48) | 1.46 (1.43 to 1.5) | <0.001 |
| Maldives | 460(356 to 576) | 382.61(296.28 to 478.83) | 2549(2005 to 3123) | 614.21(483.46 to 750.59) | 1.58 (1.54 to 1.62) | 1.55 (1.53 to 1.58) | <0.001 |
| Mali | 18935(14598 to 23888) | 358.9(277.82 to 451.2) | 79078(60309 to 100420) | 573.43(444.65 to 720.62) | 1.56 (1.54 to 1.58) | 1.52 (1.49 to 1.55) | <0.001 |
| Malta | 1724(1319 to 2176) | 409.31(313 to 516.82) | 4630(3563 to 5821) | 604.57(465.9 to 759.77) | 1.23 (1.13 to 1.33) | 1.27 (1.24 to 1.3) | <0.001 |
| Marshall Islands | 184(139 to 234) | 666.97(506.59 to 848.17) | 612(460 to 778) | 1138.08(855.3 to 1449.48) | 1.74 (1.68 to 1.8) | 1.73 (1.7 to 1.77) | <0.001 |
| Mauritania | 3612(2819 to 4488) | 286.54(224.52 to 354.69) | 10828(8609 to 13270) | 393.45(313.12 to 482.76) | 1.01 (0.9 to 1.12) | 1.02 (0.96 to 1.08) | <0.001 |
| Mauritius | 5029(3944 to 6187) | 583.15(457.07 to 717.76) | 19095(15683 to 22729) | 1058.29(863.64 to 1266.84) | 2 (1.9 to 2.1) | 1.95 (1.91 to 1.98) | <0.001 |
| Mexico | 391963(303294 to 492832) | 706.3(547.21 to 886) | 1228035(990705 to 1490711) | 908.45(731.33 to 1104.34) | 0.78 (0.73 to 0.83) | 0.8 (0.73 to 0.88) | <0.001 |
| Micronesia (Federated States of) | 369(291 to 455) | 553.74(437.85 to 681.65) | 927(731 to 1145) | 967.94(759.79 to 1199.32) | 1.89 (1.77 to 2) | 1.82 (1.78 to 1.85) | <0.001 |
| Monaco | 195(143 to 258) | 306.64(226.2 to 402.27) | 328(247 to 420) | 427.22(323 to 544.68) | 1.13 (1.11 to 1.15) | 1.08 (1.06 to 1.1) | <0.001 |
| Mongolia | 3494(2602 to 4505) | 232(171.83 to 299.91) | 11561(9043 to 14364) | 378.83(293.88 to 474.3) | 1.61 (1.54 to 1.68) | 1.6 (1.56 to 1.63) | <0.001 |
| Montenegro | 2587(2023 to 3207) | 400.59(312.44 to 497.9) | 5362(4209 to 6619) | 600.05(471.81 to 740.09) | 1.39 (1.33 to 1.45) | 1.31 (1.26 to 1.36) | <0.001 |
| Morocco | 78945(60850 to 99015) | 465.12(358.64 to 582.88) | 390881(301982 to 488784) | 1030.42(793.65 to 1291.87) | 2.63 (2.58 to 2.68) | 2.6 (2.56 to 2.64) | <0.001 |
| Mozambique | 17131(13879 to 20725) | 228.02(186.42 to 274.52) | 54061(43044 to 66428) | 327.88(263.83 to 399.19) | 1.19 (1.12 to 1.27) | 1.17 (1.14 to 1.21) | <0.001 |
| Myanmar | 120749(94856 to 149232) | 419.94(330.42 to 518.42) | 379205(308228 to 454278) | 699.71(567.51 to 840.26) | 1.64 (1.59 to 1.69) | 1.67 (1.62 to 1.71) | <0.001 |
| Namibia | 2644(2094 to 3246) | 340.17(272.72 to 414.44) | 7565(5985 to 9225) | 452.96(359 to 551.79) | 0.9 (0.82 to 0.98) | 0.93 (0.88 to 0.97) | <0.001 |
| Nauru | 43(33 to 53) | 619.71(481.22 to 769.06) | 88(68 to 109) | 983.19(759.55 to 1228.16) | 1.39 (1.32 to 1.46) | 1.49 (1.43 to 1.56) | <0.001 |
| Nepal | 37496(28653 to 47445) | 291.03(223.54 to 366.38) | 145878(112099 to 182996) | 523.07(401.83 to 656.52) | 1.84 (1.77 to 1.91) | 1.89 (1.83 to 1.96) | <0.001 |
| Netherlands | 68230(52875 to 85477) | 350.33(271.89 to 438.17) | 134926(103920 to 169258) | 445.59(342.55 to 560.7) | 0.84 (0.81 to 0.87) | 0.78 (0.74 to 0.82) | <0.001 |
| New Zealand | 16460(12326 to 21440) | 428.92(322.11 to 556.56) | 40648(32294 to 49811) | 534.35(426.56 to 651.93) | 0.85 (0.78 to 0.92) | 0.71 (0.68 to 0.74) | <0.001 |
| Nicaragua | 11365(8819 to 14169) | 532.48(413.09 to 661.8) | 49641(39649 to 60591) | 857.2(683.06 to 1047.65) | 1.57 (1.52 to 1.62) | 1.56 (1.51 to 1.62) | <0.001 |
| Niger | 11433(8966 to 14198) | 274.54(216.26 to 339.78) | 51299(40303 to 64128) | 398.84(315.07 to 494.44) | 1.23 (1.21 to 1.26) | 1.22 (1.19 to 1.25) | <0.001 |
| Nigeria | 157013(123173 to 194118) | 281.83(220.84 to 348.34) | 500815(396590 to 617152) | 393.87(313.86 to 482.19) | 1.07 (0.99 to 1.14) | 1.08 (1.06 to 1.11) | <0.001 |
| Niue | 13(10 to 16) | 611.47(470.45 to 767.99) | 20(15 to 26) | 1060.52(798.97 to 1348.19) | 1.72 (1.66 to 1.79) | 1.79 (1.77 to 1.81) | <0.001 |
| North Macedonia | 7536(6009 to 9216) | 371.96(295.49 to 456.4) | 20982(16734 to 25643) | 652.38(518.45 to 800.3) | 1.93 (1.78 to 2.09) | 1.84 (1.8 to 1.88) | <0.001 |
| Northern Mariana Islands | 159(124 to 199) | 501.43(387.39 to 631.7) | 446(345 to 560) | 782.68(601.18 to 991.44) | 1.28 (1.18 to 1.37) | 1.46 (1.42 to 1.5) | <0.001 |
| Norway | 21761(16230 to 28261) | 355.71(265.78 to 461.28) | 36400(28261 to 45651) | 430.42(331 to 544.02) | 0.63 (0.58 to 0.67) | 0.62 (0.6 to 0.64) | <0.001 |
| Oman | 4906(3826 to 6104) | 477.61(373.25 to 592.01) | 28834(22732 to 35941) | 872.03(683.98 to 1088.71) | 1.86 (1.77 to 1.96) | 1.94 (1.86 to 2.02) | <0.001 |
| Pakistan | 232275(179671 to 293280) | 318.38(246.19 to 401.55) | 938751(727036 to 1185082) | 533.81(414.46 to 671.43) | 1.88 (1.78 to 1.98) | 1.69 (1.64 to 1.74) | <0.001 |
| Palau | 83(64 to 104) | 661.17(509.73 to 828.65) | 256(200 to 315) | 1101.81(850.86 to 1367.12) | 1.64 (1.57 to 1.7) | 1.65 (1.62 to 1.68) | <0.001 |
| Palestine | 6012(4684 to 7510) | 563.27(438.23 to 703.7) | 32274(25516 to 39433) | 955.32(750.51 to 1171.4) | 1.71 (1.65 to 1.77) | 1.71 (1.68 to 1.74) | <0.001 |
| Panama | 7789(6040 to 9739) | 449.09(348.2 to 560.79) | 31691(24920 to 39149) | 719.04(565.32 to 888.54) | 1.6 (1.55 to 1.65) | 1.53 (1.43 to 1.63) | <0.001 |
| Papua New Guinea | 11366(8894 to 14078) | 412.75(326.18 to 507.84) | 56996(44248 to 70344) | 680.44(529.7 to 838.37) | 1.61 (1.58 to 1.64) | 1.62 (1.58 to 1.66) | <0.001 |
| Paraguay | 10240(7941 to 12782) | 389.06(301.78 to 485.48) | 43098(34393 to 53219) | 671.93(535.36 to 831.05) | 1.84 (1.78 to 1.9) | 1.77 (1.72 to 1.83) | <0.001 |
| Peru | 37244(28838 to 46849) | 265.14(205.15 to 333.59) | 168149(132511 to 207901) | 488.74(384.89 to 604.9) | 2.18 (2.11 to 2.25) | 2 (1.98 to 2.03) | <0.001 |
| Philippines | 160079(127852 to 196314) | 421.84(337.55 to 515.09) | 523280(422649 to 631188) | 552.66(445.27 to 666.93) | 0.9 (0.85 to 0.95) | 0.88 (0.84 to 0.92) | <0.001 |
| Poland | 153824(119784 to 191833) | 359.44(279.74 to 449.14) | 297088(232258 to 369630) | 501.45(393.42 to 623.21) | 0.83 (0.76 to 0.91) | 1.06 (0.96 to 1.15) | <0.001 |
| Portugal | 51847(38714 to 66755) | 394.49(294.79 to 508.13) | 111653(84346 to 142682) | 590.48(446.36 to 754.72) | 1.34 (1.26 to 1.42) | 1.3 (1.26 to 1.34) | <0.001 |
| Puerto Rico | 20881(16540 to 25655) | 581.65(461.22 to 714.01) | 44790(34796 to 55998) | 897.8(703.65 to 1116.11) | 1.45 (1.29 to 1.6) | 1.41 (1.38 to 1.45) | <0.001 |
| Qatar | 1568(1222 to 1972) | 734.4(575.44 to 915.38) | 29189(22601 to 36529) | 1262.11(974.76 to 1577.46) | 1.6 (1.52 to 1.68) | 1.76 (1.72 to 1.8) | <0.001 |
| Republic of Korea | 155812(122181 to 192124) | 441.73(343.13 to 549.34) | 578495(463745 to 702672) | 721.44(572.08 to 882.44) | 1.68 (1.61 to 1.75) | 1.6 (1.57 to 1.64) | <0.001 |
| Republic of Moldova | 9672(7432 to 11945) | 212.23(162.56 to 263.14) | 18733(14665 to 23187) | 373.61(292.01 to 463.07) | 1.74 (1.69 to 1.78) | 1.83 (1.72 to 1.95) | <0.001 |
| Romania | 67713(52199 to 83826) | 249.82(192.3 to 310.31) | 124350(96995 to 153790) | 408.79(319.96 to 503.86) | 1.67 (1.56 to 1.79) | 1.61 (1.54 to 1.67) | <0.001 |
| Russian Federation | 436734(343440 to 536561) | 255.22(200.37 to 314.74) | 847477(667124 to 1039098) | 421.06(331.93 to 517.31) | 1.65 (1.61 to 1.69) | 1.63 (1.6 to 1.67) | <0.001 |
| Rwanda | 9061(7257 to 11023) | 239.68(194.63 to 288.82) | 22491(17993 to 27284) | 281.41(225.57 to 340.36) | 0.44 (0.36 to 0.53) | 0.51 (0.49 to 0.54) | <0.001 |
| Saint Kitts and Nevis | 198(154 to 246) | 573.57(449.32 to 710.44) | 654(517 to 807) | 857.29(673.11 to 1064.93) | 1.32 (1.27 to 1.38) | 1.31 (1.27 to 1.34) | <0.001 |
| Saint Lucia | 633(490 to 795) | 657.97(511.04 to 823.89) | 2082(1647 to 2551) | 898.97(708.41 to 1105.15) | 0.98 (0.92 to 1.03) | 1 (0.96 to 1.04) | <0.001 |
| Saint Vincent and the Grenadines | 477(375 to 587) | 616.71(487 to 755.9) | 1232(959 to 1525) | 899.53(699.34 to 1115.82) | 1.22 (1.18 to 1.26) | 1.23 (1.19 to 1.27) | <0.001 |
| Samoa | 664(513 to 830) | 587.52(454.83 to 732.49) | 1700(1302 to 2143) | 949.23(725.79 to 1197.42) | 1.53 (1.48 to 1.58) | 1.55 (1.51 to 1.59) | <0.001 |
| San Marino | 103(76 to 133) | 307.81(229.93 to 398.06) | 255(192 to 329) | 429.29(324.2 to 552.35) | 1.12 (1.1 to 1.15) | 1.08 (1.06 to 1.1) | <0.001 |
| Sao Tome and Principe | 247(187 to 313) | 312.53(238.68 to 396.04) | 781(609 to 972) | 512.26(400.08 to 636.8) | 1.64 (1.6 to 1.68) | 1.61 (1.55 to 1.67) | <0.001 |
| Saudi Arabia | 49725(38771 to 62154) | 584.14(453 to 731.26) | 347006(273081 to 430631) | 1041.68(812.06 to 1304.63) | 1.79 (1.74 to 1.85) | 1.89 (1.84 to 1.95) | <0.001 |
| Senegal | 14877(11517 to 18623) | 343.82(267.76 to 428.02) | 55710(43744 to 68232) | 541.93(428.92 to 658.75) | 1.55 (1.48 to 1.61) | 1.5 (1.46 to 1.53) | <0.001 |
| Serbia | 43525(34910 to 52629) | 368.45(293.45 to 448.97) | 83199(64939 to 102825) | 604.4(473.09 to 743.4) | 1.72 (1.63 to 1.8) | 1.62 (1.58 to 1.65) | <0.001 |
| Seychelles | 261(201 to 325) | 441.03(340.47 to 548.6) | 1149(914 to 1407) | 919.57(727.97 to 1131) | 2.4 (2.29 to 2.52) | 2.4 (2.36 to 2.43) | <0.001 |
| Sierra Leone | 7208(5641 to 8978) | 274.53(215.13 to 341.32) | 23028(18066 to 28706) | 420.11(332.28 to 518.34) | 1.41 (1.37 to 1.46) | 1.38 (1.35 to 1.42) | <0.001 |
| Singapore | 14665(11571 to 18050) | 547.95(432.29 to 674.03) | 54727(42654 to 68003) | 678.26(524.8 to 848.44) | 0.64 (0.62 to 0.67) | 0.69 (0.65 to 0.73) | <0.001 |
| Slovakia | 18086(13982 to 22449) | 311.49(241.16 to 386.89) | 38202(29633 to 47155) | 455.75(354.79 to 562.16) | 1.24 (1.17 to 1.3) | 1.24 (1.21 to 1.27) | <0.001 |
| Slovenia | 7571(5972 to 9329) | 317.65(250.38 to 391.73) | 16270(12647 to 20481) | 452.21(353.14 to 565.21) | 1.13 (1 to 1.27) | 1.15 (1.1 to 1.19) | <0.001 |
| Solomon Islands | 918(732 to 1123) | 431.81(346.58 to 526.69) | 3473(2809 to 4195) | 667.14(541.15 to 804.43) | 1.4 (1.35 to 1.46) | 1.41 (1.37 to 1.44) | <0.001 |
| Somalia | 9050(7194 to 11106) | 245.22(197.49 to 298.44) | 32961(26179 to 40394) | 312.64(250.02 to 379.79) | 0.75 (0.71 to 0.8) | 0.78 (0.77 to 0.79) | <0.001 |
| South Africa | 92958(72965 to 115337) | 380.12(299.96 to 469.21) | 305954(247285 to 369596) | 594.75(480.71 to 718.07) | 1.5 (1.46 to 1.54) | 1.45 (1.39 to 1.51) | <0.001 |
| South Sudan | 7556(6073 to 9213) | 229.56(185.41 to 278.99) | 16168(12966 to 19774) | 301.23(243.36 to 366.73) | 0.83 (0.78 to 0.88) | 0.88 (0.87 to 0.89) | <0.001 |
| Spain | 236468(180299 to 298221) | 459.72(351.19 to 579.12) | 447638(343909 to 559028) | 570.16(437.99 to 711.6) | 0.72 (0.62 to 0.82) | 0.68 (0.59 to 0.76) | <0.001 |
| Sri Lanka | 60946(48510 to 74455) | 489.28(391.02 to 595.51) | 239156(192002 to 289301) | 874.17(697.57 to 1063) | 1.92 (1.84 to 2) | 1.88 (1.84 to 1.93) | <0.001 |
| Sudan | 46186(35592 to 57788) | 401.64(307.68 to 504.29) | 195340(152692 to 240608) | 725.86(564.86 to 897.74) | 1.89 (1.76 to 2.01) | 1.93 (1.88 to 1.97) | <0.001 |
| Suriname | 1485(1151 to 1848) | 488.2(378.38 to 606.66) | 5614(4392 to 6890) | 868.1(676.62 to 1069.93) | 2 (1.93 to 2.07) | 1.89 (1.84 to 1.94) | <0.001 |
| Sweden | 46052(34303 to 59919) | 342.9(255.4 to 446.27) | 70316(52895 to 89905) | 419.91(314.54 to 539.98) | 0.77 (0.72 to 0.82) | 0.67 (0.63 to 0.72) | <0.001 |
| Switzerland | 41047(31054 to 52593) | 422.2(320.41 to 538.78) | 86229(66068 to 109212) | 584.91(448.42 to 738.93) | 1.03 (0.98 to 1.07) | 1.06 (1.04 to 1.08) | <0.001 |
| Syrian Arab Republic | 33985(25974 to 43114) | 491.2(373.9 to 624.18) | 124969(98822 to 154559) | 840(657.99 to 1045.88) | 1.71 (1.67 to 1.75) | 1.75 (1.73 to 1.77) | <0.001 |
| Taiwan (Province of China) | 84219(67949 to 101442) | 478.96(386.11 to 576.45) | 234266(195787 to 275135) | 623.63(517.7 to 736.48) | 0.8 (0.72 to 0.87) | 0.85 (0.83 to 0.88) | <0.001 |
| Tajikistan | 7025(5442 to 8782) | 183.2(142.43 to 227.74) | 28601(22597 to 35076) | 345.81(271.19 to 425.77) | 2.26 (2.19 to 2.33) | 2.08 (2.04 to 2.12) | <0.001 |
| Thailand | 162668(126435 to 203233) | 385.8(298.98 to 482.82) | 636025(507543 to 780436) | 620.7(492.98 to 763.92) | 1.48 (1.44 to 1.53) | 1.54 (1.49 to 1.6) | <0.001 |
| Timor-Leste | 1205(952 to 1492) | 286.49(226.04 to 354.75) | 5542(4441 to 6768) | 556.92(446.24 to 679.1) | 2.25 (2.18 to 2.33) | 2.17 (2.16 to 2.19) | <0.001 |
| Togo | 4795(3728 to 5974) | 261.75(204.98 to 323.43) | 19586(15614 to 23980) | 373.81(298.08 to 458.5) | 1.11 (1.05 to 1.18) | 1.16 (1.11 to 1.21) | <0.001 |
| Tokelau | 8(6 to 10) | 605.57(465 to 768.95) | 14(11 to 18) | 999.89(766.91 to 1254.19) | 1.56 (1.5 to 1.61) | 1.63 (1.58 to 1.68) | <0.001 |
| Tonga | 391(305 to 487) | 572.34(446.7 to 710.6) | 816(637 to 1016) | 904.01(704.18 to 1125.53) | 1.44 (1.43 to 1.46) | 1.49 (1.47 to 1.51) | <0.001 |
| Trinidad and Tobago | 6469(5253 to 7733) | 684.66(556.78 to 815.31) | 17476(13697 to 21486) | 979.78(767.64 to 1206.33) | 1.18 (1.14 to 1.23) | 1.17 (1.12 to 1.22) | <0.001 |
| Tunisia | 25793(19726 to 32301) | 446.77(340.36 to 561.26) | 121494(94468 to 152104) | 887.41(687.44 to 1114.37) | 2.13 (2.03 to 2.24) | 2.23 (2.16 to 2.3) | <0.001 |
| Turkey | 155372(124156 to 189480) | 399.23(318.22 to 487.93) | 721373(571483 to 892066) | 750.12(592.28 to 930.03) | 2.05 (1.99 to 2.12) | 2.05 (2.02 to 2.08) | <0.001 |
| Turkmenistan | 5974(4631 to 7449) | 217.05(167.87 to 270.87) | 18769(15028 to 22847) | 379.83(302.72 to 464.53) | 1.91 (1.85 to 1.97) | 1.82 (1.76 to 1.89) | <0.001 |
| Tuvalu | 37(29 to 45) | 470.97(372.19 to 578.86) | 91(72 to 112) | 782.72(614.23 to 966.16) | 1.6 (1.54 to 1.66) | 1.65 (1.63 to 1.67) | <0.001 |
| Uganda | 19384(15690 to 23450) | 222.45(181.26 to 268.13) | 63001(50523 to 76921) | 295.16(239.02 to 357.14) | 0.87 (0.79 to 0.95) | 0.91 (0.87 to 0.94) | <0.001 |
| Ukraine | 123218(94317 to 155804) | 193.64(147.93 to 245.73) | 192681(147585 to 243432) | 315.95(242.69 to 399.74) | 1.56 (1.54 to 1.57) | 1.56 (1.47 to 1.64) | <0.001 |
| United Arab Emirates | 5480(4287 to 6826) | 656.07(510.12 to 821.14) | 98491(78517 to 120865) | 973.73(755.89 to 1214.72) | 1.12 (1.05 to 1.18) | 1.28 (1.2 to 1.35) | <0.001 |
| United Kingdom | 300211(224672 to 385739) | 361.49(271.18 to 464.18) | 552874(428206 to 688959) | 547.99(422.08 to 686.25) | 1.13 (1.06 to 1.19) | 1.27 (1.19 to 1.36) | <0.001 |
| United Republic of Tanzania | 31751(25516 to 38671) | 224.41(181.8 to 272.44) | 101256(81710 to 122869) | 304.97(247.37 to 368.42) | 0.96 (0.9 to 1.02) | 0.99 (0.97 to 1.01) | <0.001 |
| United States of America | 1502850(1133263 to 1925634) | 504.2(383.7 to 641.67) | 3884381(3150200 to 4691457) | 786.04(638.22 to 948.44) | 1.45 (1.39 to 1.52) | 1.45 (1.41 to 1.49) | <0.001 |
| United States Virgin Islands | 569(443 to 707) | 566.59(439.98 to 705.09) | 1263(982 to 1568) | 955.71(745.25 to 1186.84) | 1.76 (1.67 to 1.85) | 1.71 (1.66 to 1.76) | <0.001 |
| Uruguay | 12438(9582 to 15639) | 333.8(257.6 to 418.71) | 25669(20113 to 31552) | 537.21(423.93 to 657.5) | 1.68 (1.63 to 1.72) | 1.55 (1.5 to 1.59) | <0.001 |
| Uzbekistan | 43097(33084 to 53905) | 278.06(213.27 to 347.39) | 170184(134663 to 207610) | 513.79(403.83 to 630.56) | 2.17 (2.11 to 2.23) | 2.03 (1.94 to 2.12) | <0.001 |
| Vanuatu | 394(310 to 487) | 414.48(327.58 to 510.92) | 1818(1424 to 2239) | 726.62(568.17 to 896.03) | 1.77 (1.74 to 1.8) | 1.82 (1.78 to 1.86) | <0.001 |
| Venezuela (Bolivarian Republic of) | 56442(44192 to 70341) | 460.9(360.62 to 574.4) | 217124(173109 to 265665) | 710.62(564.07 to 872.36) | 1.52 (1.44 to 1.59) | 1.41 (1.39 to 1.44) | <0.001 |
| Viet Nam | 140260(111886 to 172242) | 304.35(243.59 to 372.61) | 535325(437574 to 642531) | 510.68(415.65 to 615.53) | 1.81 (1.76 to 1.87) | 1.69 (1.66 to 1.73) | <0.001 |
| Yemen | 23941(18586 to 29962) | 363.39(279.23 to 456.92) | 116973(91926 to 144988) | 603.55(472.42 to 749.66) | 1.68 (1.54 to 1.81) | 1.63 (1.55 to 1.71) | <0.001 |
| Zambia | 11843(9410 to 14533) | 294.82(237.52 to 358.27) | 42764(33281 to 53204) | 395.63(312.24 to 486.99) | 0.92 (0.87 to 0.97) | 0.95 (0.93 to 0.97) | <0.001 |
| Zimbabwe | 16052(12549 to 19882) | 311.73(245.76 to 384.05) | 40921(32702 to 50048) | 449.08(359.91 to 546.85) | 1.14 (1.05 to 1.24) | 1.18 (1.15 to 1.22) | <0.001 |

*ASR: age-standardized rate; EAPC: estimated annual percentage change; AAPC: average annual percent change; SDI: sociodemographic index; UI: uncertainty interval; CI: confidence interval.*

**Table S6. The case number and ASR of DALYs of diabetes and kidney diseases in 1990 and 2021, and its temporal trends from 1990 to 2021 in 204 countries and territories.**

| **location** | **1990** | | **2021** | | **EAPC (95% CI)** | **AAPC (95% CI)** | **P value** |
| --- | --- | --- | --- | --- | --- | --- | --- |
|  | **Case number (95% UI)** | **ASR (95% UI)** | **Case number (95% UI)** | **ASR (95% UI)** |  |  |  |
| Afghanistan | 175477(129131 to 247550) | 2357.86(1758.31 to 3325.26) | 439515(319025 to 604350) | 3452.14(2529.57 to 4777.51) | 1.34 (1.3 to 1.38) | 1.24 (1.18 to 1.31) | <0.001 |
| Albania | 17123(14011 to 20981) | 723.5(595.92 to 882.89) | 28822(22323 to 36515) | 725.12(559.15 to 922.55) | 0.11 (-0.01 to 0.22) | -0.06 (-0.28 to 0.17) | 0.617 |
| Algeria | 155482(118169 to 219052) | 1167.07(887.32 to 1683.21) | 675389(531326 to 858129) | 1877.1(1483.46 to 2373.92) | 1.76 (1.65 to 1.88) | 1.55 (1.45 to 1.65) | <0.001 |
| American Samoa | 988(796 to 1210) | 3853.48(3131.71 to 4676.94) | 3113(2566 to 3792) | 6268.21(5165.26 to 7618.63) | 1.55 (1.3 to 1.79) | 1.61 (1.53 to 1.69) | <0.001 |
| Andorra | 392(299 to 510) | 707.08(538.74 to 921.91) | 1087(824 to 1416) | 735.23(556.9 to 961.31) | 0.25 (0.11 to 0.39) | 0.09 (-0.14 to 0.31) | 0.453 |
| Angola | 127842(95845 to 165465) | 2541.2(1944.14 to 3250.73) | 390480(297121 to 510833) | 2671.32(2056.47 to 3413.35) | 0.08 (0 to 0.15) | 0.15 (0.01 to 0.28) | 0.035 |
| Antigua and Barbuda | 1535(1371 to 1726) | 2889.01(2579.12 to 3254.76) | 3530(3033 to 4109) | 3304.36(2844.39 to 3835.77) | 0.39 (0.31 to 0.47) | 0.38 (-0.03 to 0.79) | 0.068 |
| Argentina | 459283(415987 to 506509) | 1429.74(1292.97 to 1576.84) | 743354(639339 to 861865) | 1353.89(1162.61 to 1572.88) | -0.23 (-0.39 to -0.08) | -0.08 (-0.47 to 0.31) | 0.679 |
| Armenia | 26073(22282 to 30782) | 904.82(772.6 to 1067.26) | 47488(39335 to 57497) | 1154.03(955.72 to 1399.4) | 0.46 (-0.09 to 1.02) | 0.68 (-0.46 to 1.83) | 0.242 |
| Australia | 114924(98792 to 133436) | 605.27(518.95 to 703.84) | 287807(231173 to 351126) | 672.69(536.7 to 829.89) | 0.38 (0.33 to 0.43) | 0.32 (0.05 to 0.58) | 0.021 |
| Austria | 68255(59630 to 77887) | 604.68(525.4 to 694.23) | 123315(100420 to 147103) | 691.85(557.61 to 840.55) | 0.99 (0.7 to 1.28) | 0.48 (0.03 to 0.94) | 0.038 |
| Azerbaijan | 50992(42423 to 61109) | 892.33(741.49 to 1069.78) | 145196(115862 to 180443) | 1323.07(1055.03 to 1641.24) | 1.04 (0.84 to 1.25) | 1.25 (1.02 to 1.49) | <0.001 |
| Bahamas | 4178(3663 to 4744) | 2425.28(2130.8 to 2745.05) | 11842(9661 to 14381) | 2802.63(2293.78 to 3392.64) | 0.41 (0.33 to 0.48) | 0.48 (0.35 to 0.61) | <0.001 |
| Bahrain | 6360(5268 to 7602) | 3508.84(2915.2 to 4160.77) | 39562(31842 to 48555) | 4401.09(3591.56 to 5284.13) | 0.41 (0.16 to 0.67) | 0.68 (0.36 to 1.01) | <0.001 |
| Bangladesh | 991527(600735 to 1281032) | 1443.89(1066.87 to 1784.19) | 2204450(1704109 to 2784749) | 1577.44(1224.79 to 1985.36) | 0.29 (0.12 to 0.46) | 0.33 (0.08 to 0.59) | 0.011 |
| Barbados | 7797(6966 to 8737) | 2776.21(2474.82 to 3120.99) | 13923(11361 to 16984) | 2868(2333.48 to 3513) | 0.03 (-0.07 to 0.13) | 0.19 (-0.15 to 0.52) | 0.274 |
| Belarus | 46153(37165 to 56948) | 371.52(298.79 to 459.72) | 73392(57610 to 92802) | 505.72(397.94 to 639.81) | 0.38 (0.14 to 0.62) | 0.98 (0.39 to 1.57) | 0.001 |
| Belgium | 96572(80900 to 113855) | 662.39(550.04 to 787.33) | 153004(118606 to 193071) | 728.76(554.04 to 941.17) | 0.35 (0.3 to 0.4) | 0.29 (0.04 to 0.53) | 0.023 |
| Belize | 2407(2151 to 2705) | 2268.25(2030.26 to 2541.71) | 10966(9475 to 12810) | 3320.47(2876.31 to 3861.83) | 1.36 (1 to 1.73) | 1.28 (0.97 to 1.58) | <0.001 |
| Benin | 50413(38989 to 62573) | 1901.11(1523.19 to 2313.12) | 155030(119967 to 198906) | 2350.82(1861.16 to 2959.5) | 0.61 (0.53 to 0.69) | 0.7 (0.58 to 0.82) | <0.001 |
| Bermuda | 949(838 to 1083) | 1513.7(1337.28 to 1724.07) | 1629(1334 to 2007) | 1325.49(1079.37 to 1645.72) | -0.41 (-0.57 to -0.25) | -0.5 (-0.82 to -0.18) | 0.002 |
| Bhutan | 4045(2939 to 5309) | 1333.75(1000.77 to 1730.93) | 10484(8015 to 13535) | 1651.16(1264.28 to 2125.79) | 0.66 (0.62 to 0.7) | 0.68 (0.64 to 0.72) | <0.001 |
| Bolivia (Plurinational State of) | 85822(68960 to 107543) | 2307.04(1872.45 to 2863.99) | 258601(202553 to 333592) | 2766.64(2179.17 to 3552.38) | 0.58 (0.55 to 0.61) | 0.59 (0.56 to 0.63) | <0.001 |
| Bosnia and Herzegovina | 41311(34535 to 49613) | 989.86(826.96 to 1187.49) | 92931(74165 to 115107) | 1542.94(1225.99 to 1921.54) | 1.66 (1.51 to 1.82) | 1.43 (1.21 to 1.65) | <0.001 |
| Botswana | 12752(9384 to 17049) | 2120.03(1576.13 to 2799.77) | 37069(28908 to 47797) | 2429.33(1916.57 to 3099.19) | 0.59 (0.38 to 0.8) | 0.43 (-0.26 to 1.13) | 0.218 |
| Brazil | 1651295(1487100 to 1837778) | 1675.54(1507.91 to 1862.3) | 4004581(3477318 to 4605136) | 1594.76(1384.7 to 1833.2) | -0.19 (-0.24 to -0.14) | -0.1 (-0.34 to 0.14) | 0.405 |
| Brunei Darussalam | 3750(3098 to 4495) | 3163.82(2616.98 to 3781.87) | 11068(8995 to 13575) | 2931.75(2395.45 to 3569.7) | -0.16 (-0.23 to -0.08) | -0.28 (-0.52 to -0.03) | 0.03 |
| Bulgaria | 112955(96551 to 131663) | 966.61(827.82 to 1126.17) | 173778(144670 to 209940) | 1362.09(1127.89 to 1654.47) | 1.12 (0.95 to 1.29) | 1.13 (0.72 to 1.55) | <0.001 |
| Burkina Faso | 107645(83599 to 135860) | 2003.63(1593.63 to 2493.48) | 264429(202978 to 341560) | 2245.47(1763.01 to 2836.56) | 0.41 (0.33 to 0.49) | 0.36 (0.24 to 0.49) | <0.001 |
| Burundi | 70313(52474 to 90875) | 2469.1(1869.17 to 3161.89) | 124763(92562 to 167092) | 2103.34(1581.16 to 2774.34) | -0.92 (-1.07 to -0.77) | -0.55 (-0.77 to -0.34) | <0.001 |
| Cabo Verde | 2420(1946 to 2972) | 976.85(791.1 to 1193.77) | 9309(7329 to 11624) | 2001.42(1580.61 to 2486.16) | 2.14 (1.96 to 2.32) | 2.41 (2.05 to 2.78) | <0.001 |
| Cambodia | 98426(74962 to 124928) | 1690.04(1330.07 to 2113.17) | 258386(198533 to 334149) | 1944.72(1505.9 to 2493.47) | 0.38 (0.26 to 0.5) | 0.46 (0.39 to 0.53) | <0.001 |
| Cameroon | 134198(101194 to 172326) | 2427.49(1878.62 to 3065.5) | 449688(328803 to 604160) | 2877.74(2147.89 to 3808.76) | 0.42 (0.27 to 0.57) | 0.54 (0.45 to 0.63) | <0.001 |
| Canada | 199236(171016 to 231468) | 626.17(536.39 to 728.85) | 587701(460910 to 736977) | 896.18(700.52 to 1131.13) | 0.83 (0.68 to 0.99) | 1.16 (1 to 1.33) | <0.001 |
| Central African Republic | 45050(35487 to 56529) | 3192.03(2541.71 to 3953.09) | 96847(72246 to 127655) | 3439.91(2612.74 to 4406.75) | 0.23 (0.17 to 0.28) | 0.25 (0.14 to 0.35) | <0.001 |
| Chad | 52001(38985 to 68270) | 1483.35(1150.71 to 1915.67) | 160779(119251 to 213243) | 2080.26(1598.29 to 2716.89) | 1.03 (0.83 to 1.23) | 1.11 (0.91 to 1.3) | <0.001 |
| Chile | 103382(91294 to 116900) | 994.71(878.77 to 1123.01) | 287803(234750 to 350688) | 1139.99(928.76 to 1391.74) | 0.62 (0.43 to 0.81) | 0.4 (-0.13 to 0.93) | 0.138 |
| China | 8845569(7489639 to 10400349) | 958.89(814.86 to 1122.24) | 17991340(14568736 to 22002766) | 908.68(732.02 to 1115.62) | -0.27 (-0.38 to -0.17) | -0.17 (-0.26 to -0.09) | <0.001 |
| Colombia | 290436(252362 to 335936) | 1436.19(1250.14 to 1655.46) | 700543(568556 to 862157) | 1272.63(1032.85 to 1566.58) | -0.77 (-0.87 to -0.67) | -0.37 (-0.79 to 0.05) | 0.082 |
| Comoros | 5302(3706 to 6996) | 2234.36(1647.94 to 2891.13) | 12360(9310 to 15927) | 2361.83(1783.29 to 3033.17) | -0.01 (-0.15 to 0.13) | 0.13 (-0.34 to 0.61) | 0.59 |
| Congo | 40896(31746 to 51576) | 3319.13(2601.69 to 4129.68) | 104410(78915 to 135722) | 3327.91(2543.88 to 4239.18) | -0.2 (-0.32 to -0.08) | 0.02 (-0.2 to 0.23) | 0.883 |
| Cook Islands | 613(494 to 750) | 4692.04(3794.5 to 5717.09) | 1154(936 to 1419) | 4673.7(3770.77 to 5773.84) | -0.15 (-0.21 to -0.09) | -0.02 (-0.09 to 0.06) | 0.673 |
| Costa Rica | 22812(19269 to 27070) | 1197.86(1015.64 to 1414.41) | 101141(84438 to 121644) | 1845.13(1540.99 to 2219.38) | 1.23 (1.09 to 1.38) | 1.51 (0.96 to 2.07) | <0.001 |
| Coted'Ivoire | 109055(83344 to 139381) | 1979.2(1566.88 to 2474.79) | 334999(253721 to 431686) | 2410.89(1869.55 to 3058.02) | 0.53 (0.39 to 0.67) | 0.64 (0.49 to 0.8) | <0.001 |
| Croatia | 51328(43300 to 60811) | 875.48(740.81 to 1034.78) | 89626(73982 to 108758) | 1056.43(861.66 to 1299.02) | 0.34 (0.18 to 0.49) | 0.58 (0.23 to 0.94) | 0.001 |
| Cuba | 118024(101969 to 137195) | 1138.56(984.09 to 1323.04) | 226300(185072 to 278441) | 1245.15(1013.76 to 1538.2) | 0.27 (0.09 to 0.46) | 0.34 (0.06 to 0.61) | 0.015 |
| Cyprus | 14779(12081 to 17727) | 2177.55(1784.52 to 2611.06) | 23864(19161 to 29413) | 1252.7(1000.91 to 1547.54) | -2.06 (-2.18 to -1.93) | -1.74 (-1.97 to -1.5) | <0.001 |
| Czechia | 118462(101251 to 138295) | 890.6(759.78 to 1042.35) | 209989(170612 to 257151) | 1033.19(831 to 1280.57) | 1.07 (0.8 to 1.34) | 0.54 (0.31 to 0.78) | <0.001 |
| Democratic People's Republic of Korea | 181043(136873 to 232285) | 1046.13(794.83 to 1338.1) | 408864(321697 to 522289) | 1253.03(985.54 to 1602.99) | 0.61 (0.55 to 0.67) | 0.59 (0.57 to 0.61) | <0.001 |
| Democratic Republic of the Congo | 477337(362236 to 613278) | 2522.82(1950.45 to 3198.46) | 1168436(874036 to 1550418) | 2670.21(2013.46 to 3461.54) | 0.12 (0.04 to 0.21) | 0.19 (0.12 to 0.26) | <0.001 |
| Denmark | 39854(34573 to 45978) | 535.06(461.69 to 621.06) | 78546(65258 to 92940) | 709.98(579.23 to 856.59) | 0.9 (0.71 to 1.09) | 0.99 (0.58 to 1.4) | <0.001 |
| Djibouti | 3022(2214 to 4059) | 1702.51(1272.24 to 2253.53) | 16094(11695 to 21839) | 2256.25(1672.02 to 3002.79) | 0.88 (0.75 to 1.01) | 0.89 (0.82 to 0.95) | <0.001 |
| Dominica | 1763(1515 to 2055) | 2968.06(2549.49 to 3460.75) | 3179(2587 to 3857) | 3880.53(3155.99 to 4710.1) | 0.8 (0.75 to 0.85) | 0.85 (0.75 to 0.96) | <0.001 |
| Dominican Republic | 64951(53764 to 78357) | 1461.01(1213.28 to 1761.24) | 234310(184452 to 293688) | 2257.49(1778.3 to 2828.93) | 1.81 (1.69 to 1.92) | 1.46 (1.2 to 1.72) | <0.001 |
| Ecuador | 85486(76219 to 95931) | 1422.47(1268.5 to 1592.06) | 364907(292301 to 455823) | 2211.96(1776.09 to 2755.54) | 1.3 (0.75 to 1.85) | 1.24 (0.96 to 1.53) | <0.001 |
| Egypt | 598809(477284 to 787004) | 1991.24(1597.34 to 2669.51) | 2142868(1738346 to 2625659) | 3332.59(2721.88 to 4060.77) | 2.1 (1.95 to 2.26) | 1.68 (1.29 to 2.08) | <0.001 |
| El Salvador | 55747(47187 to 67251) | 1630.93(1385.31 to 1962.89) | 217623(173676 to 268586) | 3531.95(2820.38 to 4358.62) | 2.54 (2.28 to 2.8) | 2.4 (1.61 to 3.2) | <0.001 |
| Equatorial Guinea | 6503(4885 to 8494) | 2845.44(2168.43 to 3661.24) | 21597(14889 to 30637) | 3318.27(2325.81 to 4530.32) | 0.6 (0.3 to 0.9) | 0.57 (0.39 to 0.74) | <0.001 |
| Eritrea | 35491(26072 to 46828) | 2332.72(1750.12 to 3028.8) | 85027(61699 to 114525) | 2541.96(1885.68 to 3337.59) | 0.27 (0.21 to 0.33) | 0.26 (0.11 to 0.4) | 0.001 |
| Estonia | 12108(10261 to 14227) | 643.48(547.07 to 754.61) | 26000(21831 to 31152) | 1093.16(912.81 to 1320.53) | 1.17 (0.83 to 1.5) | 1.69 (1.27 to 2.1) | <0.001 |
| Eswatini | 9728(7531 to 12275) | 3056.26(2384.78 to 3835.21) | 29073(20910 to 39309) | 4730.33(3473.4 to 6318.31) | 1.76 (1.13 to 2.4) | 1.44 (1.25 to 1.63) | <0.001 |
| Ethiopia | 912742(750022 to 1082487) | 3650.97(3061.09 to 4275.69) | 1092920(909328 to 1298721) | 2092.48(1756.34 to 2462.95) | -2.27 (-2.46 to -2.08) | -1.78 (-1.95 to -1.62) | <0.001 |
| Fiji | 25190(20400 to 30901) | 6163.65(5002.27 to 7534.63) | 70206(55060 to 88968) | 8700.55(6858.31 to 10955.84) | 0.95 (0.75 to 1.14) | 1.08 (0.89 to 1.28) | <0.001 |
| Finland | 36274(29017 to 44847) | 541.81(430.49 to 674.03) | 74290(56279 to 95482) | 728.71(538.86 to 958.27) | 0.98 (0.85 to 1.1) | 0.98 (0.9 to 1.06) | <0.001 |
| France | 373246(321249 to 429029) | 464.47(397.37 to 537.37) | 698568(565186 to 840313) | 528.46(419.53 to 653.29) | 0.5 (0.3 to 0.7) | 0.51 (0.33 to 0.68) | <0.001 |
| Gabon | 18685(14562 to 23847) | 3079.38(2406.23 to 3905.45) | 44828(31824 to 59262) | 3940.34(2798.68 to 5125.02) | 0.76 (0.62 to 0.89) | 0.82 (0.64 to 1.01) | <0.001 |
| Gambia | 8082(6058 to 10565) | 1724.43(1330.02 to 2221.86) | 29558(22441 to 38478) | 2485.32(1911.3 to 3191.73) | 1.05 (0.93 to 1.18) | 1.16 (0.71 to 1.62) | <0.001 |
| Georgia | 47429(39865 to 56642) | 771.82(646.82 to 923.34) | 74780(62270 to 91158) | 1386.65(1152.92 to 1695.17) | 2.32 (2.01 to 2.63) | 1.9 (1.22 to 2.59) | <0.001 |
| Germany | 818534(716464 to 928921) | 669.38(582.43 to 764.11) | 1443724(1172962 to 1727331) | 787.54(629.71 to 961.77) | 0.56 (0.4 to 0.71) | 0.64 (0.31 to 0.96) | <0.001 |
| Ghana | 127876(96502 to 169859) | 1612.73(1246.87 to 2119.13) | 516857(394985 to 668687) | 2648.12(2050.2 to 3376.2) | 1.98 (1.74 to 2.22) | 1.61 (1.54 to 1.68) | <0.001 |
| Greece | 114207(96482 to 134299) | 800.6(672.17 to 946.16) | 215301(176928 to 257485) | 970.72(779.12 to 1194.53) | 0.42 (0.09 to 0.74) | 0.64 (0.18 to 1.11) | 0.006 |
| Greenland | 273(220 to 337) | 756.27(613.8 to 924.54) | 564(452 to 704) | 840.8(671.02 to 1053.57) | 0.45 (0.29 to 0.6) | 0.42 (0.32 to 0.52) | <0.001 |
| Grenada | 2517(2221 to 2822) | 3567.8(3149.3 to 4003.78) | 4947(4225 to 5757) | 4315.29(3686.87 to 5007.93) | 0.71 (0.59 to 0.82) | 0.72 (0.24 to 1.2) | 0.003 |
| Guam | 1608(1342 to 1913) | 1922.81(1607.32 to 2276.9) | 4125(3430 to 4969) | 2054.17(1705.75 to 2478.64) | 0.58 (0.37 to 0.78) | 0.17 (-0.02 to 0.37) | 0.085 |
| Guatemala | 77124(68734 to 86747) | 1711.63(1530.3 to 1918.4) | 449900(384168 to 523309) | 3790.45(3237.73 to 4401.69) | 2.58 (2.32 to 2.84) | 2.64 (2.08 to 3.2) | <0.001 |
| Guinea | 70860(52391 to 91151) | 1765.88(1354.06 to 2248.33) | 148876(113855 to 194505) | 2181.91(1690.13 to 2821.45) | 0.73 (0.63 to 0.82) | 0.68 (0.6 to 0.77) | <0.001 |
| Guinea-Bissau | 14457(10741 to 18586) | 2724.56(2097.57 to 3438.51) | 28059(21450 to 36708) | 2971.61(2320.39 to 3774.46) | 0.28 (0.2 to 0.35) | 0.31 (0.27 to 0.35) | <0.001 |
| Guyana | 16031(13858 to 18607) | 3665.99(3186.18 to 4233.92) | 34877(28058 to 43123) | 5065.62(4090.61 to 6235.58) | 1.16 (0.91 to 1.41) | 1.09 (0.77 to 1.42) | <0.001 |
| Haiti | 141183(107823 to 179993) | 3681.2(2860.06 to 4659.7) | 320562(233781 to 440231) | 3834.1(2813.52 to 5218.53) | 0.25 (0.2 to 0.31) | 0.17 (0.06 to 0.28) | 0.002 |
| Honduras | 29795(24183 to 36619) | 1221.64(996.24 to 1499.24) | 145340(112962 to 185203) | 2117.23(1660.61 to 2676.69) | 1.92 (1.8 to 2.04) | 1.81 (1.51 to 2.12) | <0.001 |
| Hungary | 118443(99619 to 139313) | 849.22(714.87 to 1000.15) | 182364(148563 to 223668) | 1001.46(808.98 to 1239.27) | 0.74 (0.59 to 0.9) | 0.57 (0.03 to 1.11) | 0.037 |
| Iceland | 1080(883 to 1316) | 383.63(312.07 to 469.59) | 2973(2282 to 3807) | 558.73(422.33 to 725.96) | 1.26 (1.2 to 1.32) | 1.23 (1.13 to 1.34) | <0.001 |
| India | 6575165(5694216 to 7549286) | 1252.4(1087.45 to 1431.33) | 20159744(17353500 to 23377422) | 1625.05(1404.07 to 1875.71) | 0.83 (0.7 to 0.96) | 0.85 (0.64 to 1.07) | <0.001 |
| Indonesia | 1704469(1386518 to 2023835) | 1399.67(1158.71 to 1659.42) | 4736116(3919404 to 5633240) | 1839.17(1525.82 to 2183.55) | 0.83 (0.81 to 0.86) | 0.9 (0.82 to 0.99) | <0.001 |
| Iran (Islamic Republic of) | 289580(242938 to 354148) | 997.73(841.71 to 1218.84) | 1163938(975889 to 1393478) | 1472.18(1241.26 to 1749.52) | 1.56 (1.46 to 1.67) | 1.28 (1.14 to 1.42) | <0.001 |
| Iraq | 227165(179191 to 284888) | 2448.81(1954.17 to 3054.84) | 817975(627597 to 1042958) | 3132.47(2430.45 to 3946.67) | 0.65 (0.55 to 0.75) | 0.81 (0.65 to 0.97) | <0.001 |
| Ireland | 25370(21377 to 30081) | 640.7(536.69 to 762.54) | 46853(36375 to 59114) | 633.99(487.06 to 807.68) | 0.07 (0.01 to 0.14) | 0.01 (-0.28 to 0.3) | 0.944 |
| Israel | 54198(47382 to 61976) | 1141.99(994.52 to 1307.73) | 135354(110882 to 161923) | 1110.56(906.93 to 1339.84) | -0.14 (-0.57 to 0.29) | -0.13 (-0.48 to 0.23) | 0.473 |
| Italy | 710535(621796 to 809961) | 836.26(725.4 to 961.17) | 1002562(812362 to 1209626) | 742.41(591.26 to 921.38) | -0.32 (-0.37 to -0.27) | -0.41 (-0.61 to -0.22) | <0.001 |
| Jamaica | 46460(41806 to 51609) | 2564.6(2307.87 to 2848.3) | 91967(73883 to 114065) | 2964.52(2382.79 to 3677.72) | 0.14 (-0.14 to 0.42) | 0.59 (-0.45 to 1.63) | 0.268 |
| Japan | 1162118(980242 to 1373435) | 710.64(598.85 to 839.17) | 2318120(1817729 to 2903071) | 752.29(577.62 to 965.43) | 0.1 (0.04 to 0.16) | 0.19 (0.07 to 0.31) | 0.002 |
| Jordan | 36894(30030 to 45198) | 2432.97(1986.88 to 2969.62) | 197985(156012 to 247589) | 2457.61(1954.22 to 3047.11) | -0.22 (-0.45 to 0.02) | 0.08 (-0.4 to 0.57) | 0.733 |
| Kazakhstan | 110734(92554 to 132962) | 801.87(667.33 to 965.11) | 222291(178690 to 276664) | 1198.17(966.39 to 1484.79) | 0.77 (0.53 to 1.01) | 1.37 (1 to 1.75) | <0.001 |
| Kenya | 134187(110248 to 171226) | 1334.94(1110.57 to 1699.19) | 478942(396097 to 581976) | 1858.98(1540.4 to 2240.98) | 1.27 (1.16 to 1.39) | 1.07 (0.89 to 1.24) | <0.001 |
| Kiribati | 2147(1709 to 2649) | 5087.4(4047.04 to 6276.76) | 5608(4288 to 7367) | 6854.88(5265.88 to 8891.23) | 0.87 (0.7 to 1.03) | 0.98 (0.93 to 1.02) | <0.001 |
| Kuwait | 13613(11378 to 16168) | 1752.92(1480.2 to 2047.97) | 72839(53838 to 95464) | 2018.48(1532.38 to 2585.45) | 0.46 (0.27 to 0.65) | 0.33 (-0.72 to 1.39) | 0.54 |
| Kyrgyzstan | 23390(19454 to 27912) | 686.38(568.56 to 820.76) | 56194(45780 to 68974) | 1011.46(822.49 to 1240.57) | 0.52 (0.18 to 0.86) | 1.12 (0.74 to 1.51) | <0.001 |
| Lao People's Democratic Republic | 65807(48016 to 87254) | 2604.22(1968.61 to 3397.67) | 133327(101532 to 172596) | 2582.86(1989.61 to 3299.67) | -0.16 (-0.21 to -0.11) | -0.03 (-0.08 to 0.02) | 0.193 |
| Latvia | 17658(14804 to 21127) | 533.15(448.32 to 636.6) | 33189(27626 to 40180) | 976.53(811.04 to 1187.35) | 1.62 (1.31 to 1.93) | 1.96 (1.34 to 2.58) | <0.001 |
| Lebanon | 44166(35145 to 55966) | 2001.13(1599.44 to 2522.1) | 131483(104690 to 163804) | 2171.74(1725.68 to 2709.63) | 0.49 (0.38 to 0.61) | 0.3 (0.1 to 0.51) | 0.003 |
| Lesotho | 15129(11637 to 19420) | 1714.76(1322.73 to 2197.53) | 44230(32616 to 58124) | 3866.63(2875.95 to 5045.17) | 3.46 (3 to 3.93) | 2.72 (2.41 to 3.04) | <0.001 |
| Liberia | 33133(23591 to 44483) | 2185.49(1662.19 to 2844.93) | 72430(54397 to 94976) | 2647.99(2022.28 to 3418.94) | 0.78 (0.6 to 0.96) | 0.61 (0.38 to 0.84) | <0.001 |
| Libya | 28154(21751 to 36102) | 1298.24(1008.62 to 1661.12) | 140568(107589 to 179090) | 2461.27(1889.63 to 3115.58) | 2.59 (2.43 to 2.76) | 2.1 (1.9 to 2.31) | <0.001 |
| Lithuania | 19132(15698 to 23185) | 447.16(367.61 to 541.24) | 38637(31882 to 47340) | 792.32(652.44 to 975.18) | 1.33 (1.05 to 1.62) | 1.83 (1.34 to 2.31) | <0.001 |
| Luxembourg | 3395(2911 to 3967) | 659.52(562 to 773.83) | 7335(5843 to 9140) | 717.22(565.2 to 904.43) | 0.35 (0.27 to 0.43) | 0.28 (-0.02 to 0.57) | 0.067 |
| Madagascar | 104948(82745 to 131110) | 1679.56(1337.27 to 2095.68) | 242126(181407 to 313744) | 1735.67(1311.76 to 2232.42) | 0.13 (0.06 to 0.19) | 0.13 (-0.18 to 0.44) | 0.408 |
| Malawi | 110328(83969 to 139501) | 2176.95(1703.9 to 2724.9) | 220364(170742 to 280152) | 2402.3(1898.48 to 3001.09) | 0.1 (-0.13 to 0.32) | 0.33 (0.22 to 0.45) | <0.001 |
| Malaysia | 176323(147567 to 209273) | 1714.32(1439.17 to 2026.7) | 554008(458118 to 661267) | 1895.23(1565.9 to 2261.31) | 0.17 (0.06 to 0.28) | 0.36 (0.17 to 0.56) | <0.001 |
| Maldives | 2680(2145 to 3338) | 2458.52(1992.45 to 3095.45) | 5998(4798 to 7433) | 1580.4(1271.34 to 1942.53) | -1.68 (-1.9 to -1.47) | -1.48 (-1.66 to -1.31) | <0.001 |
| Mali | 107905(82413 to 136462) | 2185.4(1739.71 to 2717.87) | 287889(223919 to 368858) | 2632.56(2082.71 to 3323.86) | 0.7 (0.65 to 0.75) | 0.63 (0.51 to 0.75) | <0.001 |
| Malta | 3962(3451 to 4549) | 957.16(831.22 to 1099.31) | 9112(7367 to 11170) | 1044.85(830.19 to 1302.75) | 0.3 (0.12 to 0.49) | 0.23 (-0.28 to 0.74) | 0.381 |
| Marshall Islands | 840(652 to 1085) | 4411.7(3441.71 to 5661.92) | 3056(2163 to 4452) | 7386.07(5232.24 to 10723.36) | 1.64 (1.42 to 1.86) | 1.68 (1.64 to 1.72) | <0.001 |
| Mauritania | 22449(17144 to 28574) | 1944.1(1503.99 to 2456.65) | 47536(35301 to 63132) | 2000.31(1496.39 to 2638.96) | -0.15 (-0.26 to -0.04) | 0.09 (-0.03 to 0.2) | 0.145 |
| Mauritius | 24897(22564 to 27517) | 3125.33(2828.71 to 3454.26) | 103352(93632 to 114151) | 5710.69(5163.36 to 6317.23) | 2.69 (2.18 to 3.19) | 1.76 (1.07 to 2.45) | <0.001 |
| Mexico | 1560743(1422128 to 1723422) | 3281.91(3001.88 to 3605.11) | 5114222(4500676 to 5923349) | 3914.28(3448.73 to 4525.87) | 0.63 (0.51 to 0.76) | 0.48 (0.24 to 0.73) | <0.001 |
| Micronesia (Federated States of) | 1988(1511 to 2618) | 3677.29(2815.71 to 4816) | 4580(3531 to 5957) | 5581.03(4323.65 to 7228.69) | 1.35 (1.09 to 1.61) | 1.38 (1.28 to 1.47) | <0.001 |
| Monaco | 245(193 to 307) | 395.22(307.19 to 501.93) | 513(399 to 649) | 609.31(465.36 to 784.29) | 1.46 (1.4 to 1.52) | 1.41 (1.33 to 1.5) | <0.001 |
| Mongolia | 12336(10033 to 15215) | 939.64(771.27 to 1133.93) | 29545(24248 to 35933) | 1088.07(894.4 to 1317.41) | 0.31 (0.23 to 0.4) | 0.47 (0.04 to 0.9) | 0.033 |
| Montenegro | 6401(5269 to 7738) | 1024.35(843.35 to 1237.98) | 12531(10131 to 15517) | 1349.84(1086.39 to 1674.39) | 0.92 (0.83 to 1.01) | 0.95 (0.78 to 1.12) | <0.001 |
| Morocco | 199184(149862 to 276881) | 1245.98(943.92 to 1759.66) | 864512(671311 to 1095978) | 2456.54(1913.98 to 3105) | 2.44 (2.35 to 2.53) | 2.21 (2.12 to 2.29) | <0.001 |
| Mozambique | 135722(102080 to 177007) | 1764.57(1370.82 to 2256.29) | 346911(252414 to 470202) | 2389.83(1783.39 to 3132.66) | 1.46 (1.3 to 1.62) | 1 (0.91 to 1.09) | <0.001 |
| Myanmar | 773095(570617 to 1001490) | 2833.67(2135.98 to 3639.16) | 1417739(1121050 to 1775515) | 2810.2(2231.11 to 3508.28) | -0.29 (-0.37 to -0.2) | -0.03 (-0.1 to 0.05) | 0.518 |
| Namibia | 14582(11569 to 18487) | 2128.61(1696.98 to 2678.02) | 37083(27910 to 48386) | 2601.77(1981.17 to 3354.76) | 0.44 (0.15 to 0.73) | 0.72 (0.47 to 0.97) | <0.001 |
| Nauru | 261(197 to 346) | 4800.63(3653.28 to 6338.11) | 454(347 to 587) | 6703.83(5146.46 to 8690.27) | 0.92 (0.76 to 1.08) | 1.08 (1.02 to 1.14) | <0.001 |
| Nepal | 145261(112597 to 182250) | 1317.25(1037.49 to 1641.03) | 462374(359735 to 585625) | 1890.6(1476.96 to 2387.12) | 1.2 (0.97 to 1.44) | 1.17 (1.09 to 1.25) | <0.001 |
| Netherlands | 142901(122060 to 165249) | 738.84(628.96 to 857.78) | 220661(176231 to 270863) | 687.18(539.81 to 857.87) | -0.33 (-0.47 to -0.19) | -0.25 (-0.39 to -0.11) | 0.001 |
| New Zealand | 26192(22475 to 30614) | 694.48(594.69 to 812.99) | 59754(49253 to 71769) | 766.74(629.21 to 926.64) | 0.2 (0.1 to 0.29) | 0.36 (0.16 to 0.56) | <0.001 |
| Nicaragua | 38293(32169 to 45941) | 1937.42(1643.86 to 2296.96) | 163095(132952 to 195745) | 3068.73(2511.34 to 3675.7) | 1.73 (1.5 to 1.96) | 1.44 (0.89 to 2) | <0.001 |
| Niger | 68425(49347 to 90278) | 1582.55(1213.63 to 2041.89) | 180924(135034 to 243834) | 1712.98(1310.24 to 2260.01) | 0.23 (0.16 to 0.3) | 0.28 (0.17 to 0.4) | <0.001 |
| Nigeria | 969932(765987 to 1179061) | 1851.59(1516.34 to 2218.32) | 2129326(1655382 to 2728546) | 1923.73(1558.7 to 2382.77) | 0.02 (-0.03 to 0.07) | 0.12 (0.04 to 0.2) | 0.002 |
| Niue | 75(59 to 94) | 3456.84(2719.37 to 4359.53) | 121(93 to 154) | 5818.68(4457.94 to 7414.45) | 1.49 (1.36 to 1.63) | 1.69 (1.56 to 1.82) | <0.001 |
| North Macedonia | 23324(19192 to 27895) | 1236.2(1017.84 to 1476.68) | 52112(41853 to 64316) | 1623.76(1304 to 2003.85) | 0.91 (0.7 to 1.13) | 0.9 (0.78 to 1.01) | <0.001 |
| Northern Mariana Islands | 678(522 to 863) | 2943.5(2322.26 to 3679.59) | 1987(1653 to 2395) | 3653.12(3036.96 to 4408.56) | 0.73 (0.62 to 0.84) | 0.63 (0.52 to 0.73) | <0.001 |
| Norway | 33149(27306 to 40198) | 542.58(442.86 to 664.24) | 54105(43033 to 67566) | 602.12(471.87 to 764.55) | 0.2 (0.07 to 0.33) | 0.32 (-0.07 to 0.72) | 0.107 |
| Oman | 14858(11224 to 19603) | 1842.97(1392.16 to 2429.6) | 58510(46593 to 73229) | 2511.76(2010.94 to 3130.13) | 1.3 (1.15 to 1.45) | 0.99 (0.76 to 1.22) | <0.001 |
| Pakistan | 948311(761257 to 1151408) | 1493.23(1215.58 to 1803.99) | 3386752(2704224 to 4147192) | 2396.25(1931.9 to 2918.55) | 1.39 (1.17 to 1.6) | 1.54 (1.4 to 1.68) | <0.001 |
| Palau | 374(290 to 474) | 3531.42(2751.95 to 4455.03) | 1182(933 to 1485) | 5133.97(4054.15 to 6434.48) | 1.28 (1.15 to 1.41) | 1.23 (1.18 to 1.28) | <0.001 |
| Palestine | 22619(17476 to 29026) | 2392.34(1861.96 to 3057.45) | 71085(58546 to 85889) | 2688.72(2227.45 to 3226.49) | 0.4 (0.27 to 0.53) | 0.34 (0.19 to 0.48) | <0.001 |
| Panama | 20364(17507 to 23857) | 1256.05(1082.08 to 1467.02) | 92450(74693 to 111929) | 2089.61(1688.97 to 2529.48) | 1.64 (1.46 to 1.82) | 1.71 (1.55 to 1.86) | <0.001 |
| Papua New Guinea | 66769(46691 to 90611) | 3095.05(2222.21 to 4168.47) | 223898(173268 to 284929) | 3599.93(2800.47 to 4565.75) | 0.48 (0.45 to 0.5) | 0.48 (0.45 to 0.51) | <0.001 |
| Paraguay | 33310(27676 to 39858) | 1355.69(1129.31 to 1615.59) | 148913(117929 to 186690) | 2487.65(1971.42 to 3116.14) | 2.14 (1.93 to 2.35) | 2.06 (1.88 to 2.23) | <0.001 |
| Peru | 169892(141665 to 201961) | 1219.01(1021.33 to 1446.55) | 490646(381725 to 618647) | 1436.34(1118.65 to 1809.3) | 0.37 (0.19 to 0.54) | 0.59 (-0.23 to 1.41) | 0.158 |
| Philippines | 700074(613472 to 793410) | 1921.73(1696.96 to 2167.68) | 2313206(1981265 to 2669637) | 2580.12(2216.28 to 2973.65) | 1.2 (1.13 to 1.27) | 0.97 (0.76 to 1.18) | <0.001 |
| Poland | 461794(406665 to 528291) | 1077.51(948.25 to 1233.73) | 655859(535490 to 798560) | 981.14(796.62 to 1202.53) | -0.28 (-0.47 to -0.09) | -0.34 (-0.58 to -0.11) | 0.004 |
| Portugal | 134575(117408 to 154355) | 1023.3(888.2 to 1177.47) | 235680(188453 to 289628) | 1037.28(812.56 to 1307.79) | -0.12 (-0.3 to 0.07) | -0.05 (-0.62 to 0.51) | 0.852 |
| Puerto Rico | 92400(82327 to 104156) | 2579(2296.5 to 2907.08) | 173626(144924 to 209331) | 2812.85(2338.84 to 3416.82) | 0.22 (0.14 to 0.29) | 0.17 (0.02 to 0.31) | 0.023 |
| Qatar | 3655(2812 to 4725) | 2997.59(2319.74 to 3873.13) | 38757(29405 to 50327) | 3316.26(2592.8 to 4171.94) | 0.18 (-0.33 to 0.7) | 0.34 (-0.39 to 1.07) | 0.361 |
| Republic of Korea | 408328(344793 to 480164) | 1260.8(1063.88 to 1480.81) | 1048525(792737 to 1363050) | 1196.42(898.85 to 1563.49) | -0.35 (-0.46 to -0.24) | -0.18 (-0.38 to 0.03) | 0.092 |
| Republic of Moldova | 26698(21723 to 32561) | 602.13(488.29 to 736.04) | 49971(39737 to 62665) | 903.95(718.42 to 1134.69) | 0.86 (0.62 to 1.09) | 1.25 (0.68 to 1.83) | <0.001 |
| Romania | 201401(173405 to 234753) | 752.57(648.58 to 876.3) | 258488(211946 to 315391) | 763.28(622.42 to 938.89) | 0.25 (0.04 to 0.45) | 0.03 (-0.24 to 0.29) | 0.852 |
| Russian Federation | 975741(847079 to 1132291) | 567.31(492.38 to 658.27) | 2028702(1753120 to 2393382) | 893.29(769.39 to 1057.31) | 0.86 (0.45 to 1.28) | 1.56 (1.05 to 2.07) | <0.001 |
| Rwanda | 100123(76183 to 127503) | 2811.9(2152.47 to 3570.62) | 143384(102723 to 195144) | 2016.19(1449.33 to 2721.15) | -1.99 (-2.34 to -1.65) | -1.1 (-1.3 to -0.9) | <0.001 |
| Saint Kitts and Nevis | 1250(1126 to 1395) | 3430.54(3084.98 to 3834.27) | 2366(1927 to 2856) | 3377.97(2768.93 to 4051.93) | 0.3 (0.15 to 0.45) | -0.02 (-0.32 to 0.28) | 0.893 |
| Saint Lucia | 3449(3076 to 3876) | 3858.28(3445.74 to 4327.41) | 7977(6577 to 9653) | 3390.01(2796.18 to 4096.73) | -0.71 (-0.85 to -0.58) | -0.54 (-1.06 to -0.02) | 0.041 |
| Saint Vincent and the Grenadines | 2662(2372 to 2997) | 3615.69(3222.48 to 4067.64) | 5322(4547 to 6279) | 3818.2(3263.43 to 4499.42) | 0 (-0.14 to 0.14) | 0.13 (-0.54 to 0.8) | 0.703 |
| Samoa | 3022(2374 to 3846) | 3264.91(2587.54 to 4123.39) | 7141(5672 to 8874) | 4623.81(3688.73 to 5721.03) | 1.08 (1.02 to 1.15) | 1.14 (1.08 to 1.19) | <0.001 |
| San Marino | 158(126 to 196) | 471.6(372.59 to 589.16) | 360(267 to 476) | 550.03(402.73 to 736.36) | 0.77 (0.67 to 0.88) | 0.45 (0.34 to 0.56) | <0.001 |
| Sao Tome and Principe | 1495(1131 to 1906) | 1898.23(1484.08 to 2362.43) | 3270(2433 to 4273) | 2549.14(1912.2 to 3234.54) | 0.91 (0.81 to 1.01) | 0.94 (0.77 to 1.12) | <0.001 |
| Saudi Arabia | 141690(107937 to 183726) | 1986.34(1528.12 to 2563.79) | 802967(632124 to 1011323) | 3212.55(2559.97 to 3969.25) | 1.43 (1.32 to 1.54) | 1.57 (1.42 to 1.71) | <0.001 |
| Senegal | 87485(68163 to 110845) | 2100.66(1673.4 to 2621.71) | 230734(177231 to 301202) | 2636.39(2040.41 to 3392.28) | 0.78 (0.71 to 0.85) | 0.78 (0.71 to 0.86) | <0.001 |
| Serbia | 135561(111044 to 163948) | 1275.62(1046.43 to 1543.02) | 220878(179895 to 270520) | 1399.4(1134.47 to 1726.43) | 0.19 (0.1 to 0.28) | 0.3 (0.1 to 0.51) | 0.003 |
| Seychelles | 933(783 to 1100) | 1603.32(1347.18 to 1889.93) | 3236(2651 to 3917) | 2749.9(2255.55 to 3325.46) | 1.92 (1.77 to 2.08) | 1.75 (1.49 to 2.02) | <0.001 |
| Sierra Leone | 42042(30386 to 54988) | 1604.45(1217.05 to 2057.08) | 90839(69121 to 121427) | 1941.61(1502.4 to 2547.84) | 0.72 (0.63 to 0.81) | 0.62 (0.52 to 0.72) | <0.001 |
| Singapore | 28261(23661 to 33939) | 1188.63(1003.51 to 1414.53) | 76674(56433 to 102574) | 912.34(672.66 to 1219.69) | -0.83 (-0.93 to -0.74) | -0.89 (-1.28 to -0.51) | <0.001 |
| Slovakia | 52052(43253 to 62270) | 885.09(734.66 to 1060.38) | 74540(59782 to 91893) | 832.45(665.88 to 1031.42) | -0.19 (-0.24 to -0.13) | -0.19 (-0.36 to -0.01) | 0.039 |
| Slovenia | 18211(15262 to 21865) | 756.9(633.46 to 908.97) | 29535(23485 to 37032) | 718.18(565.99 to 910.7) | -0.74 (-0.92 to -0.56) | -0.2 (-0.58 to 0.18) | 0.305 |
| Solomon Islands | 5236(3162 to 7573) | 3211.14(2042.81 to 4547.12) | 18484(14024 to 24002) | 4393.87(3365.54 to 5659.82) | 1.04 (0.98 to 1.09) | 0.99 (0.77 to 1.22) | <0.001 |
| Somalia | 86561(61135 to 119575) | 2620.12(1912.32 to 3511.04) | 233592(168466 to 323707) | 2804.59(2077.12 to 3752.46) | 0.27 (0.19 to 0.35) | 0.24 (0.17 to 0.3) | <0.001 |
| South Africa | 434836(382966 to 492434) | 1885.33(1666.47 to 2126.76) | 1450626(1297620 to 1615347) | 3058.88(2738.87 to 3399.41) | 1.79 (1.46 to 2.13) | 1.53 (1.11 to 1.94) | <0.001 |
| South Sudan | 68632(48567 to 92676) | 2241.32(1635.37 to 3002.51) | 139595(99414 to 188782) | 2910.72(2101.38 to 3880.73) | 0.76 (0.56 to 0.95) | 0.86 (0.73 to 0.99) | <0.001 |
| Spain | 498369(418045 to 589930) | 951.3(794.12 to 1130.45) | 801197(616258 to 1020153) | 877.05(660.15 to 1144.04) | -0.28 (-0.31 to -0.25) | -0.27 (-0.34 to -0.2) | <0.001 |
| Sri Lanka | 224244(187310 to 265930) | 1986.66(1665.37 to 2349.5) | 690396(520547 to 883232) | 2584.41(1953.72 to 3302.69) | 1.14 (0.97 to 1.3) | 0.87 (0.46 to 1.27) | <0.001 |
| Sudan | 136292(97201 to 196604) | 1178.45(875.02 to 1729.34) | 406156(308563 to 526023) | 1755.06(1356.34 to 2254.89) | 1.42 (1.3 to 1.54) | 1.29 (1.24 to 1.34) | <0.001 |
| Suriname | 6523(5404 to 7621) | 2334.46(1951.91 to 2719.21) | 21359(17006 to 26622) | 3326(2645.38 to 4144.22) | 1.32 (1.19 to 1.46) | 1.19 (0.82 to 1.56) | <0.001 |
| Sweden | 73773(61900 to 87831) | 546.37(453.39 to 658.36) | 129849(104697 to 160602) | 666.45(528.06 to 843.05) | 0.85 (0.78 to 0.93) | 0.62 (0.4 to 0.84) | <0.001 |
| Switzerland | 72212(60249 to 85992) | 721.96(597.17 to 867.67) | 134753(103078 to 171400) | 807.27(605.6 to 1050.57) | 0.31 (0.23 to 0.38) | 0.35 (0.09 to 0.6) | 0.008 |
| Syrian Arab Republic | 111879(86337 to 142234) | 1707.43(1339.46 to 2166.35) | 276839(213506 to 352387) | 2110.89(1619.31 to 2695.47) | 0.41 (0.21 to 0.61) | 0.69 (0.52 to 0.85) | <0.001 |
| Taiwan (Province of China) | 275243(245472 to 309118) | 1704.61(1521.23 to 1907.51) | 614986(512417 to 726994) | 1509.82(1257.09 to 1790.96) | -0.77 (-0.98 to -0.57) | -0.36 (-0.7 to -0.01) | 0.045 |
| Tajikistan | 23031(18846 to 28159) | 712.75(584.18 to 867.23) | 66904(52792 to 84583) | 952.32(754.53 to 1196.86) | 0.62 (0.45 to 0.79) | 0.95 (0.8 to 1.1) | <0.001 |
| Thailand | 617962(501620 to 758856) | 1560.07(1270.96 to 1903.13) | 1941852(1549801 to 2413693) | 1877.85(1498.4 to 2336.81) | 0.11 (-0.09 to 0.31) | 0.63 (0.42 to 0.84) | <0.001 |
| Timor-Leste | 5903(4087 to 7915) | 1459.95(1095.54 to 1907.35) | 17541(13405 to 22995) | 1909.2(1469.66 to 2487.89) | 1 (0.78 to 1.22) | 0.88 (0.77 to 1) | <0.001 |
| Togo | 27121(21006 to 34677) | 1573.7(1250.42 to 1985.1) | 89302(66715 to 117405) | 1984.28(1508.91 to 2572.11) | 0.7 (0.59 to 0.8) | 0.76 (0.63 to 0.9) | <0.001 |
| Tokelau | 44(33 to 58) | 3268.16(2494.95 to 4333.27) | 66(52 to 83) | 4531.09(3580.8 to 5735.3) | 0.9 (0.8 to 1) | 1.07 (0.99 to 1.16) | <0.001 |
| Tonga | 1881(1508 to 2299) | 3195.05(2569.88 to 3896.51) | 3542(2800 to 4478) | 4289.8(3396.81 to 5414.18) | 0.9 (0.78 to 1.02) | 0.96 (0.8 to 1.12) | <0.001 |
| Trinidad and Tobago | 39961(36086 to 44187) | 4617.52(4173.44 to 5098.09) | 87077(69919 to 107617) | 4591.41(3686.65 to 5676.71) | -0.21 (-0.33 to -0.09) | 0.04 (-0.2 to 0.28) | 0.759 |
| Tunisia | 56308(44008 to 73948) | 1052.86(826.93 to 1383.04) | 237058(182142 to 309567) | 1797.02(1382.08 to 2348.27) | 1.74 (1.66 to 1.83) | 1.73 (1.66 to 1.79) | <0.001 |
| Turkey | 643940(514718 to 803286) | 1749.96(1408.91 to 2177.82) | 1566713(1252774 to 1929678) | 1697.16(1358.13 to 2086.93) | 0.16 (-0.15 to 0.46) | -0.11 (-0.5 to 0.28) | 0.57 |
| Turkmenistan | 22504(19652 to 25810) | 926.41(808.37 to 1063.51) | 77553(63248 to 95456) | 1681.26(1373.54 to 2063.05) | 1.61 (1.37 to 1.86) | 2.05 (1.23 to 2.88) | <0.001 |
| Tuvalu | 248(190 to 330) | 3456.44(2657.6 to 4579.28) | 481(382 to 611) | 4448.01(3538.69 to 5654.32) | 0.83 (0.76 to 0.9) | 0.82 (0.77 to 0.88) | <0.001 |
| Uganda | 143316(104721 to 194952) | 1837.62(1353.4 to 2496.26) | 400820(294734 to 535111) | 2191.83(1635.89 to 2896.18) | 0.16 (-0.04 to 0.36) | 0.54 (0.38 to 0.7) | <0.001 |
| Ukraine | 253921(202311 to 314851) | 381.22(303.31 to 473.59) | 398682(302264 to 507116) | 583.22(442.92 to 740.56) | 0.95 (0.78 to 1.12) | 1.38 (0.97 to 1.8) | <0.001 |
| United Arab Emirates | 10692(8194 to 14005) | 1900.95(1475.54 to 2470.44) | 111431(83798 to 143703) | 2285.68(1739.67 to 2852.36) | 1.74 (1.32 to 2.17) | 0.58 (-0.63 to 1.8) | 0.351 |
| United Kingdom | 469244(396181 to 556363) | 571.1(478.13 to 682.8) | 862009(668719 to 1097484) | 792.84(603.66 to 1026.12) | 1.07 (0.9 to 1.23) | 1.04 (0.91 to 1.18) | <0.001 |
| United Republic of Tanzania | 259257(203301 to 328172) | 1901.28(1510.75 to 2391.21) | 608157(462995 to 794035) | 1993.51(1544.42 to 2577.45) | 0.08 (0.04 to 0.12) | 0.16 (0.08 to 0.25) | <0.001 |
| United States of America | 2732124(2390352 to 3139210) | 899.53(785.62 to 1035.9) | 8054223(6761016 to 9587578) | 1504.08(1261.44 to 1795.68) | 1.61 (1.48 to 1.74) | 1.69 (1.56 to 1.83) | <0.001 |
| United States Virgin Islands | 1965(1607 to 2387) | 2210.34(1809.63 to 2674.48) | 3938(3053 to 5042) | 2533.07(1953.36 to 3259.28) | 0.57 (0.47 to 0.67) | 0.45 (0.25 to 0.65) | <0.001 |
| Uruguay | 34129(30583 to 38147) | 894.56(799.1 to 1002.72) | 60323(51018 to 70909) | 1142.14(960.56 to 1354.14) | 0.81 (0.71 to 0.91) | 0.77 (0.47 to 1.07) | <0.001 |
| Uzbekistan | 106881(88504 to 129516) | 790.75(656.28 to 956.83) | 515616(431170 to 622300) | 1735.45(1454.46 to 2089.78) | 2.19 (1.81 to 2.57) | 2.47 (1.97 to 2.96) | <0.001 |
| Vanuatu | 2038(1481 to 2920) | 2809.8(2078.3 to 3981.47) | 8156(6435 to 10357) | 4081.94(3242.11 to 5148.84) | 1.13 (1.08 to 1.18) | 1.18 (0.97 to 1.39) | <0.001 |
| Venezuela (Bolivarian Republic of) | 182983(162101 to 207183) | 1681.42(1491.38 to 1896.9) | 804407(639217 to 995109) | 2670.62(2126.23 to 3297.73) | 1.28 (1.14 to 1.41) | 1.42 (1.04 to 1.79) | <0.001 |
| Viet Nam | 662006(506653 to 849747) | 1534.71(1194.46 to 1957.97) | 1778729(1386405 to 2231066) | 1815.2(1418.47 to 2265.7) | 0.69 (0.59 to 0.79) | 0.54 (0.51 to 0.57) | <0.001 |
| Yemen | 64370(45495 to 97779) | 1030.27(751.28 to 1572.26) | 214724(159426 to 298874) | 1306.92(983.34 to 1812.41) | 0.73 (0.62 to 0.84) | 0.78 (0.64 to 0.93) | <0.001 |
| Zambia | 97415(76533 to 120990) | 2563.41(2048.94 to 3169.53) | 252095(182846 to 346420) | 2789.74(2087.65 to 3667.11) | -0.09 (-0.25 to 0.06) | 0.3 (0.18 to 0.41) | <0.001 |
| Zimbabwe | 71762(56595 to 90902) | 1648.43(1309.01 to 2066.21) | 228511(175383 to 295352) | 2970.44(2304.59 to 3779.93) | 2.39 (1.89 to 2.9) | 2.03 (1.66 to 2.4) | <0.001 |

*DALYs: disability-adjusted life years; ASR: age-standardized rate; EAPC: estimated annual percentage change; AAPC: average annual percent change; SDI: sociodemographic index; UI: uncertainty interval; CI: confidence interval.*

**Table S7. The case number of deaths and ASR of mortality rate of diabetes and kidney diseases in 1990 and 2021, and its temporal trends from 1990 to 2021 in 204 countries and territories.**

| **location** | **1990** | | **2021** | | **EAPC (95% CI)** | **AAPC (95% CI)** | **P value** |
| --- | --- | --- | --- | --- | --- | --- | --- |
|  | **Case number (95% UI)** | **ASR (95% UI)** | **Case number (95% UI)** | **ASR (95% UI)** |  |  |  |
| Afghanistan | 4752(3331 to 7070) | 73.19(51.69 to 110.8) | 8569(5639 to 13204) | 94.55(62.86 to 147.03) | 0.94 (0.9 to 0.99) | 0.83 (0.74 to 0.92) | <0.001 |
| Albania | 348(286 to 422) | 17.99(14.86 to 21.71) | 647(483 to 835) | 15.77(11.7 to 20.36) | -0.2 (-0.46 to 0.05) | -0.5 (-0.88 to -0.12) | 0.01 |
| Algeria | 3553(2481 to 5744) | 39.83(27.85 to 66) | 15276(11693 to 19569) | 56.21(43.05 to 71.77) | 1.63 (1.42 to 1.84) | 1.18 (0.9 to 1.46) | <0.001 |
| American Samoa | 25(20 to 32) | 126.9(100.61 to 158) | 82(65 to 101) | 186.87(150.15 to 230.01) | 1.26 (0.97 to 1.55) | 1.37 (1.19 to 1.55) | <0.001 |
| Andorra | 12(9 to 17) | 25.34(17.78 to 35.38) | 32(21 to 44) | 18.18(12.33 to 25.09) | -0.75 (-0.98 to -0.52) | -1.14 (-1.64 to -0.64) | <0.001 |
| Angola | 3121(2283 to 4165) | 87.1(64.74 to 113.97) | 8979(6445 to 12035) | 88.21(64.07 to 115.58) | -0.1 (-0.2 to 0) | 0.07 (-0.09 to 0.23) | 0.387 |
| Antigua and Barbuda | 55(49 to 60) | 98.16(88.06 to 108.17) | 104(92 to 114) | 106.08(93.77 to 116.57) | 0.18 (0.05 to 0.32) | 0.2 (-0.49 to 0.89) | 0.573 |
| Argentina | 16585(15170 to 17951) | 54(49.12 to 58.54) | 24733(21810 to 27143) | 42.97(37.93 to 47.14) | -0.73 (-0.98 to -0.49) | -0.77 (-1.32 to -0.22) | 0.006 |
| Armenia | 529(488 to 571) | 19.84(18.18 to 21.55) | 1083(937 to 1244) | 25.48(22.02 to 29.32) | 0.41 (-0.47 to 1.3) | 0.18 (-1.38 to 1.77) | 0.819 |
| Australia | 3801(3366 to 4157) | 20.35(17.89 to 22.32) | 9408(7852 to 10505) | 18.15(15.3 to 20.23) | -0.21 (-0.36 to -0.07) | -0.4 (-0.91 to 0.13) | 0.137 |
| Austria | 2619(2320 to 2892) | 21.3(18.81 to 23.54) | 5425(4412 to 6142) | 23.64(19.51 to 26.67) | 1.44 (0.96 to 1.92) | 0.43 (-0.29 to 1.15) | 0.243 |
| Azerbaijan | 984(801 to 1190) | 18.97(15.36 to 23.03) | 2810(2116 to 3529) | 28.65(21.4 to 36.06) | 1.2 (0.87 to 1.53) | 1.31 (0.91 to 1.72) | <0.001 |
| Bahamas | 111(99 to 124) | 73.32(65.22 to 81.52) | 285(229 to 353) | 74.79(60.39 to 91.86) | -0.06 (-0.19 to 0.07) | 0.14 (-0.15 to 0.42) | 0.35 |
| Bahrain | 178(145 to 216) | 148.85(120.72 to 179.72) | 929(741 to 1128) | 179.2(144.55 to 214.33) | 0.32 (-0.07 to 0.72) | 0.57 (-0.03 to 1.17) | 0.062 |
| Bangladesh | 21591(14702 to 27767) | 44.42(33.78 to 55.79) | 55293(40679 to 72847) | 48.33(35.89 to 63.11) | 0.08 (-0.32 to 0.49) | 0.43 (-0.21 to 1.07) | 0.187 |
| Barbados | 295(267 to 322) | 97.53(88.35 to 106.54) | 465(363 to 569) | 90.5(70.67 to 110.9) | -0.29 (-0.47 to -0.1) | -0.14 (-0.51 to 0.23) | 0.465 |
| Belarus | 677(603 to 757) | 5.34(4.75 to 5.97) | 1077(873 to 1300) | 7.04(5.7 to 8.51) | -0.5 (-1.14 to 0.15) | 0.8 (-1.31 to 2.96) | 0.458 |
| Belgium | 3263(2852 to 3606) | 20.7(18.01 to 22.9) | 4287(3435 to 4881) | 14.3(11.7 to 16.18) | -0.93 (-1.07 to -0.78) | -1.05 (-1.31 to -0.8) | <0.001 |
| Belize | 70(63 to 77) | 73.36(66.03 to 80.57) | 290(250 to 332) | 100.75(86.77 to 114.95) | 1.18 (0.68 to 1.68) | 1.06 (0.61 to 1.51) | <0.001 |
| Benin | 1323(1029 to 1653) | 65.74(52.08 to 81.3) | 3611(2706 to 4748) | 76.17(58.25 to 98.24) | 0.44 (0.31 to 0.56) | 0.5 (0.34 to 0.65) | <0.001 |
| Bermuda | 31(28 to 34) | 52.57(47.23 to 57.94) | 48(39 to 59) | 32.98(27 to 40.62) | -1.46 (-1.63 to -1.28) | -1.6 (-2.02 to -1.18) | <0.001 |
| Bhutan | 92(64 to 127) | 41.2(29.17 to 56.69) | 300(209 to 408) | 53.05(37.09 to 71.77) | 0.85 (0.8 to 0.9) | 0.83 (0.71 to 0.96) | <0.001 |
| Bolivia (Plurinational State of) | 2525(1999 to 3199) | 82.88(65.94 to 104.66) | 8338(6299 to 11031) | 100.97(76.88 to 132.65) | 0.7 (0.65 to 0.76) | 0.67 (0.59 to 0.76) | <0.001 |
| Bosnia and Herzegovina | 963(805 to 1146) | 25.97(21.58 to 31.01) | 2693(2083 to 3393) | 42.01(32.4 to 52.99) | 1.91 (1.69 to 2.13) | 1.72 (1.37 to 2.07) | <0.001 |
| Botswana | 390(275 to 534) | 80.98(57.93 to 109.72) | 1083(807 to 1444) | 88.76(67.27 to 116.95) | 0.54 (0.28 to 0.8) | 0.28 (-0.68 to 1.25) | 0.574 |
| Brazil | 41668(39115 to 43523) | 50.25(46.48 to 52.74) | 110459(99509 to 117291) | 45.24(40.6 to 48.11) | -0.3 (-0.4 to -0.2) | -0.26 (-0.63 to 0.1) | 0.161 |
| Brunei Darussalam | 111(89 to 137) | 122.41(97.89 to 150.85) | 233(190 to 283) | 87.81(71.06 to 106.63) | -0.64 (-0.82 to -0.46) | -1.18 (-1.53 to -0.82) | <0.001 |
| Bulgaria | 2816(2544 to 3106) | 25.38(22.94 to 27.98) | 4855(4124 to 5652) | 34.12(28.88 to 39.91) | 1 (0.73 to 1.26) | 1.03 (0.27 to 1.8) | 0.008 |
| Burkina Faso | 2914(2243 to 3714) | 74.33(57.97 to 93.69) | 6635(4945 to 8722) | 78.02(59.28 to 100.65) | 0.19 (0.12 to 0.26) | 0.17 (0.04 to 0.31) | 0.014 |
| Burundi | 2076(1533 to 2707) | 92.92(69.34 to 120.34) | 3397(2448 to 4662) | 80.3(58.73 to 108.92) | -0.9 (-1.05 to -0.74) | -0.48 (-0.64 to -0.32) | <0.001 |
| Cabo Verde | 65(50 to 81) | 26.74(20.66 to 33.27) | 274(202 to 352) | 64.77(47.95 to 83.02) | 2.48 (2.17 to 2.78) | 2.98 (2.33 to 3.64) | <0.001 |
| Cambodia | 2371(1787 to 3056) | 50.89(39.1 to 65) | 6392(4575 to 8771) | 56.38(40.81 to 76.44) | 0.29 (0.15 to 0.43) | 0.35 (0.26 to 0.44) | <0.001 |
| Cameroon | 3669(2733 to 4772) | 91.11(68.9 to 117.35) | 11457(7966 to 15964) | 102.34(73.18 to 139.87) | 0.22 (0.07 to 0.37) | 0.37 (0.26 to 0.49) | <0.001 |
| Canada | 6627(5864 to 7270) | 20.71(18.26 to 22.75) | 14216(12055 to 15809) | 17.9(15.34 to 19.85) | -0.77 (-1.12 to -0.43) | -0.46 (-0.86 to -0.06) | 0.025 |
| Central African Republic | 1154(892 to 1460) | 109.82(85.5 to 137.68) | 2185(1553 to 2973) | 110.56(80 to 146.31) | 0.03 (-0.03 to 0.09) | 0.05 (-0.11 to 0.2) | 0.558 |
| Chad | 1381(1014 to 1882) | 50.12(37.37 to 67.98) | 3700(2637 to 5138) | 68.07(50.04 to 92.74) | 0.91 (0.69 to 1.14) | 1.03 (0.78 to 1.27) | <0.001 |
| Chile | 3045(2786 to 3309) | 32.35(29.45 to 35.21) | 8150(7055 to 8982) | 31.05(26.91 to 34.22) | 0.17 (-0.16 to 0.5) | -0.26 (-0.84 to 0.31) | 0.368 |
| China | 183666(159033 to 213048) | 25.01(21.68 to 29.01) | 388442(317638 to 465906) | 20.12(16.41 to 24.11) | -0.72 (-0.86 to -0.58) | -0.72 (-0.88 to -0.56) | <0.001 |
| Colombia | 6787(6195 to 7354) | 40.4(36.68 to 43.82) | 15966(12965 to 18953) | 28.7(23.41 to 34.03) | -1.49 (-1.66 to -1.33) | -1.26 (-1.5 to -1.02) | <0.001 |
| Comoros | 149(104 to 198) | 83.49(60.79 to 109.11) | 370(264 to 495) | 86.28(62.08 to 114.91) | -0.06 (-0.16 to 0.04) | 0.07 (-0.08 to 0.22) | 0.36 |
| Congo | 1163(888 to 1475) | 122.86(93.56 to 154.64) | 2728(1971 to 3628) | 119.19(86.84 to 154.85) | -0.3 (-0.41 to -0.18) | -0.09 (-0.32 to 0.14) | 0.448 |
| Cook Islands | 19(15 to 24) | 165.87(131.65 to 207.16) | 34(27 to 43) | 134.98(105.25 to 169.45) | -0.83 (-0.94 to -0.73) | -0.64 (-0.75 to -0.54) | <0.001 |
| Costa Rica | 516(464 to 565) | 30.04(26.92 to 32.92) | 2646(2238 to 3013) | 47.88(40.65 to 54.44) | 1.2 (0.9 to 1.5) | 1.86 (1.29 to 2.42) | <0.001 |
| Coted'Ivoire | 2555(1920 to 3294) | 71.64(55.39 to 90.11) | 7990(5786 to 10753) | 82.62(61.68 to 108.24) | 0.35 (0.18 to 0.52) | 0.47 (0.3 to 0.64) | <0.001 |
| Croatia | 1290(1167 to 1421) | 23.58(21.24 to 26) | 2900(2481 to 3285) | 29.23(24.93 to 33.3) | 0.15 (-0.24 to 0.55) | 0.66 (-0.04 to 1.36) | 0.066 |
| Cuba | 3075(2791 to 3371) | 30.65(27.77 to 33.6) | 5261(4475 to 6069) | 26.28(22.36 to 30.33) | -0.35 (-0.73 to 0.04) | -0.4 (-1.02 to 0.22) | 0.209 |
| Cyprus | 646(522 to 783) | 120.47(98.06 to 145.27) | 864(693 to 1042) | 50.67(40.46 to 61.17) | -3.08 (-3.3 to -2.86) | -2.7 (-3.18 to -2.21) | <0.001 |
| Czechia | 3204(2916 to 3504) | 23.3(21.15 to 25.49) | 5979(5060 to 6858) | 25.72(21.75 to 29.54) | 1.47 (0.93 to 2.02) | 0.47 (-0.46 to 1.42) | 0.324 |
| Democratic People's Republic of Korea | 4165(2900 to 5716) | 28.68(20.12 to 39.38) | 9023(6561 to 12295) | 28.82(20.94 to 39.39) | 0.15 (0.02 to 0.29) | 0.03 (-0.03 to 0.08) | 0.325 |
| Democratic Republic of the Congo | 12599(9381 to 16500) | 93.14(70.4 to 120.36) | 29596(20904 to 40429) | 93.58(66.32 to 125.98) | -0.05 (-0.16 to 0.05) | 0 (-0.12 to 0.13) | 0.952 |
| Denmark | 1306(1163 to 1437) | 15.65(13.92 to 17.24) | 3101(2630 to 3475) | 22.67(19.34 to 25.35) | 1.27 (0.94 to 1.6) | 1.27 (0.56 to 1.99) | <0.001 |
| Djibouti | 77(55 to 106) | 66.12(48.43 to 89.06) | 448(312 to 627) | 88.87(63.75 to 120.57) | 0.94 (0.82 to 1.05) | 0.94 (0.91 to 0.98) | <0.001 |
| Dominica | 62(53 to 73) | 105.07(88.78 to 123.32) | 99(79 to 122) | 124.86(100.26 to 153.36) | 0.44 (0.38 to 0.5) | 0.55 (0.43 to 0.67) | <0.001 |
| Dominican Republic | 1447(1171 to 1777) | 40.54(32.86 to 49.73) | 5256(3875 to 6905) | 52.94(39.04 to 69.56) | 1.45 (1.25 to 1.65) | 1.03 (0.6 to 1.46) | <0.001 |
| Ecuador | 2379(2177 to 2576) | 46.78(42.74 to 50.63) | 11010(8439 to 14316) | 70.89(54.78 to 91.39) | 1.35 (0.64 to 2.07) | 1.17 (0.77 to 1.56) | <0.001 |
| Egypt | 17481(13679 to 24068) | 81.92(63.73 to 116.44) | 61159(47847 to 76642) | 127.83(101.15 to 159.1) | 2.06 (1.85 to 2.28) | 1.47 (1.12 to 1.82) | <0.001 |
| El Salvador | 1457(1213 to 1840) | 47.56(39.6 to 60.18) | 7109(5396 to 9020) | 112.27(85.38 to 142.46) | 2.79 (2.45 to 3.13) | 2.81 (2.03 to 3.6) | <0.001 |
| Equatorial Guinea | 178(131 to 236) | 100.28(74.44 to 130.67) | 539(343 to 785) | 119.84(77.43 to 167.97) | 0.74 (0.43 to 1.06) | 0.7 (0.51 to 0.88) | <0.001 |
| Eritrea | 865(620 to 1159) | 83.3(60.77 to 110.21) | 2167(1512 to 2997) | 93.67(67.37 to 126) | 0.38 (0.3 to 0.46) | 0.37 (0.23 to 0.51) | <0.001 |
| Estonia | 225(204 to 246) | 11.64(10.58 to 12.73) | 836(698 to 968) | 28.12(23.62 to 32.6) | 2.07 (1.44 to 2.7) | 2.86 (2.04 to 3.69) | <0.001 |
| Eswatini | 301(226 to 387) | 119.25(89.8 to 152.79) | 866(602 to 1203) | 173.3(123.07 to 236.66) | 1.62 (0.98 to 2.27) | 1.24 (1.04 to 1.44) | <0.001 |
| Ethiopia | 25179(20882 to 29739) | 136.49(113.79 to 160.6) | 31521(25822 to 37960) | 80.34(65.9 to 96.08) | -2.18 (-2.36 to -1.99) | -1.7 (-1.84 to -1.57) | <0.001 |
| Fiji | 728(578 to 908) | 220.78(175.23 to 274.9) | 2132(1606 to 2748) | 314.07(239.35 to 400.06) | 0.92 (0.67 to 1.17) | 1.06 (0.88 to 1.25) | <0.001 |
| Finland | 772(674 to 859) | 10.78(9.38 to 12.02) | 1428(1155 to 1632) | 9.22(7.6 to 10.49) | -0.36 (-0.64 to -0.09) | -0.48 (-0.79 to -0.18) | 0.002 |
| France | 15171(13339 to 16597) | 17.17(15.09 to 18.8) | 28116(22999 to 31665) | 14.51(12.07 to 16.31) | -0.31 (-0.67 to 0.04) | -0.51 (-0.98 to -0.02) | 0.039 |
| Gabon | 607(460 to 787) | 116.1(87.62 to 150.22) | 1302(852 to 1759) | 146.75(95.41 to 194.64) | 0.7 (0.55 to 0.85) | 0.8 (0.6 to 1.01) | <0.001 |
| Gambia | 201(147 to 270) | 61.46(46.17 to 80.97) | 787(576 to 1054) | 87.42(64.86 to 115.74) | 1.03 (0.92 to 1.14) | 1.12 (0.72 to 1.52) | <0.001 |
| Georgia | 921(804 to 1049) | 14.89(12.99 to 16.98) | 1785(1525 to 2074) | 30.09(25.73 to 35.01) | 3.15 (2.62 to 3.68) | 2.34 (1.09 to 3.61) | <0.001 |
| Germany | 32248(28498 to 35534) | 24.18(21.3 to 26.67) | 61047(49507 to 69920) | 24.76(20.35 to 28.22) | 0.36 (0.22 to 0.5) | 0.15 (-0.38 to 0.68) | 0.577 |
| Ghana | 3229(2355 to 4407) | 57.08(42.56 to 76.98) | 13901(10278 to 18273) | 95.52(71.83 to 123.82) | 2.08 (1.83 to 2.33) | 1.69 (1.58 to 1.81) | <0.001 |
| Greece | 4168(3715 to 4577) | 29.3(25.89 to 32.21) | 8817(7428 to 9854) | 27.65(23.63 to 30.8) | -0.64 (-1.41 to 0.14) | -0.18 (-1.26 to 0.91) | 0.747 |
| Greenland | 8(6 to 10) | 28.43(22.43 to 35.48) | 13(10 to 17) | 23.9(17.82 to 31.42) | -0.34 (-0.47 to -0.21) | -0.43 (-0.55 to -0.31) | <0.001 |
| Grenada | 93(82 to 104) | 121.04(106.76 to 135.49) | 147(125 to 168) | 141.47(120.22 to 161.13) | 0.69 (0.43 to 0.94) | 0.68 (-0.16 to 1.52) | 0.115 |
| Guam | 41(34 to 49) | 66.03(53.93 to 80.18) | 91(72 to 110) | 42.86(34.47 to 51.78) | -0.74 (-1.08 to -0.4) | -1.52 (-2.04 to -1) | <0.001 |
| Guatemala | 1729(1585 to 1873) | 53.84(49.39 to 58.25) | 12279(10288 to 14398) | 114.08(95.63 to 133.42) | 2.32 (1.98 to 2.67) | 2.55 (1.78 to 3.32) | <0.001 |
| Guinea | 1966(1441 to 2597) | 61.82(46.05 to 81.41) | 3970(2900 to 5379) | 75.79(56.25 to 101.1) | 0.74 (0.62 to 0.87) | 0.67 (0.56 to 0.77) | <0.001 |
| Guinea-Bissau | 360(265 to 468) | 93.18(70.6 to 119.4) | 652(481 to 859) | 101.03(76.33 to 129.57) | 0.28 (0.2 to 0.36) | 0.27 (0.2 to 0.34) | <0.001 |
| Guyana | 408(356 to 462) | 110.32(96.78 to 124.65) | 866(658 to 1100) | 142.32(108.9 to 179.47) | 0.97 (0.65 to 1.3) | 0.83 (0.37 to 1.29) | <0.001 |
| Haiti | 3639(2707 to 4792) | 117.33(88.19 to 154.37) | 7403(4918 to 11067) | 111.54(74.92 to 164.32) | -0.03 (-0.08 to 0.03) | -0.12 (-0.23 to -0.01) | 0.028 |
| Honduras | 567(445 to 720) | 27.73(21.72 to 35.35) | 3192(2355 to 4251) | 54.18(40.45 to 71.43) | 2.38 (2.16 to 2.6) | 2.26 (1.69 to 2.83) | <0.001 |
| Hungary | 2805(2551 to 3061) | 19.71(17.88 to 21.5) | 4979(4283 to 5615) | 23.69(20.39 to 26.75) | 1.08 (0.82 to 1.35) | 0.85 (-0.17 to 1.88) | 0.103 |
| Iceland | 29(25 to 32) | 9.44(8.25 to 10.52) | 68(55 to 78) | 10.05(8.16 to 11.43) | 0.42 (0.32 to 0.52) | 0.25 (-0.01 to 0.5) | 0.058 |
| India | 151331(131182 to 170975) | 36.23(31.08 to 41.04) | 507140(440549 to 570481) | 46.99(40.79 to 52.83) | 0.92 (0.72 to 1.11) | 0.89 (0.36 to 1.42) | 0.001 |
| Indonesia | 37927(30635 to 46488) | 38.01(30.84 to 47.45) | 113404(90443 to 139547) | 52.3(41.82 to 64.46) | 1.08 (1 to 1.17) | 1.05 (0.95 to 1.15) | <0.001 |
| Iran (Islamic Republic of) | 6601(5473 to 8434) | 31.87(26.21 to 41.16) | 28145(24425 to 31341) | 41.31(35.6 to 46.12) | 1.16 (1.02 to 1.3) | 0.81 (0.66 to 0.96) | <0.001 |
| Iraq | 6087(4588 to 7982) | 77.29(58.54 to 101.17) | 17995(12751 to 23427) | 91.98(66.57 to 117.48) | 0.22 (0.02 to 0.41) | 0.63 (0.33 to 0.93) | <0.001 |
| Ireland | 773(692 to 848) | 19.54(17.4 to 21.46) | 1132(927 to 1287) | 13.23(10.86 to 15.03) | -0.85 (-1 to -0.7) | -1.29 (-2.09 to -0.48) | 0.002 |
| Israel | 1947(1748 to 2116) | 43.13(38.3 to 46.99) | 5230(4318 to 5880) | 37.51(31.27 to 42.09) | -0.48 (-1.11 to 0.15) | -0.52 (-1.12 to 0.08) | 0.091 |
| Italy | 25775(23206 to 27232) | 28.6(25.62 to 30.29) | 38277(31024 to 42732) | 19.68(16.34 to 21.85) | -1.05 (-1.15 to -0.95) | -1.22 (-1.5 to -0.94) | <0.001 |
| Jamaica | 1677(1517 to 1830) | 91.11(82.46 to 99.41) | 3111(2376 to 3944) | 96.85(74.1 to 122.89) | -0.13 (-0.46 to 0.2) | 0.38 (-0.95 to 1.72) | 0.577 |
| Japan | 29699(26946 to 31178) | 18.98(17 to 20.04) | 63074(49265 to 71507) | 11.76(9.7 to 13.07) | -1.59 (-1.76 to -1.43) | -1.53 (-1.84 to -1.22) | <0.001 |
| Jordan | 970(760 to 1227) | 86.11(67.51 to 108.76) | 4105(3153 to 5222) | 70.93(54.67 to 89.61) | -1 (-1.35 to -0.65) | -0.75 (-1.37 to -0.12) | 0.019 |
| Kazakhstan | 1702(1527 to 1890) | 12.94(11.59 to 14.38) | 3510(2973 to 4065) | 21.39(18.12 to 24.74) | 0.75 (0.36 to 1.13) | 1.65 (1.11 to 2.19) | <0.001 |
| Kenya | 3971(3217 to 5197) | 52.85(42.91 to 69.21) | 14539(11731 to 17937) | 74.92(60.14 to 91.93) | 1.34 (1.25 to 1.43) | 1.14 (0.94 to 1.35) | <0.001 |
| Kiribati | 60(46 to 75) | 172.12(131.5 to 218.72) | 148(108 to 200) | 228.02(167.25 to 302.68) | 0.8 (0.62 to 0.98) | 0.93 (0.88 to 0.99) | <0.001 |
| Kuwait | 268(234 to 300) | 52.68(45.06 to 58.95) | 939(722 to 1147) | 41.33(31.62 to 50.5) | -1 (-1.38 to -0.61) | -1.2 (-3.54 to 1.21) | 0.326 |
| Kyrgyzstan | 347(304 to 395) | 10.71(9.38 to 12.19) | 892(733 to 1079) | 18.1(14.87 to 21.86) | 0.57 (0.03 to 1.1) | 1.77 (1.18 to 2.36) | <0.001 |
| Lao People's Democratic Republic | 1694(1219 to 2278) | 82.11(60.4 to 109.18) | 3325(2432 to 4461) | 77.58(57.55 to 102.86) | -0.32 (-0.37 to -0.26) | -0.19 (-0.26 to -0.11) | <0.001 |
| Latvia | 319(288 to 352) | 9.36(8.44 to 10.32) | 884(746 to 1023) | 21.65(18.27 to 25.07) | 2.38 (1.8 to 2.97) | 2.79 (1.88 to 3.72) | <0.001 |
| Lebanon | 1318(1011 to 1707) | 70.12(54.03 to 90.22) | 4001(3127 to 5040) | 61.46(47.96 to 77.59) | -0.17 (-0.31 to -0.02) | -0.41 (-0.59 to -0.23) | <0.001 |
| Lesotho | 510(378 to 673) | 65.07(48.18 to 86.14) | 1403(1007 to 1886) | 143.7(103.99 to 191.35) | 3.52 (3 to 4.03) | 2.72 (2.31 to 3.12) | <0.001 |
| Liberia | 865(631 to 1153) | 78.24(59.56 to 101.62) | 1753(1251 to 2384) | 91.96(67.03 to 122.21) | 0.69 (0.52 to 0.86) | 0.5 (0.33 to 0.68) | <0.001 |
| Libya | 745(540 to 1036) | 41.71(30.21 to 57.97) | 3294(2270 to 4422) | 71.71(49.99 to 95.38) | 2.54 (2.29 to 2.79) | 1.78 (1.44 to 2.12) | <0.001 |
| Lithuania | 296(268 to 325) | 6.82(6.18 to 7.49) | 901(769 to 1046) | 15.66(13.37 to 18.24) | 1.62 (0.99 to 2.26) | 2.66 (1.56 to 3.77) | <0.001 |
| Luxembourg | 115(104 to 126) | 22.02(19.82 to 24.09) | 231(189 to 268) | 18.77(15.51 to 21.64) | -0.28 (-0.4 to -0.16) | -0.43 (-1.06 to 0.2) | 0.182 |
| Madagascar | 2950(2289 to 3775) | 62.18(48.45 to 79.85) | 6016(4256 to 8084) | 62.64(44.86 to 83.38) | 0.04 (-0.03 to 0.1) | 0.04 (-0.29 to 0.38) | 0.79 |
| Malawi | 3001(2288 to 3821) | 83.79(64.6 to 106.35) | 6320(4854 to 8055) | 93.63(72.85 to 117.86) | 0.16 (-0.07 to 0.38) | 0.39 (0.25 to 0.52) | <0.001 |
| Malaysia | 4456(3638 to 5367) | 49.62(40.46 to 59.74) | 12790(10385 to 15294) | 48.85(39.36 to 58.57) | -0.27 (-0.47 to -0.08) | 0.05 (-0.36 to 0.46) | 0.81 |
| Maldives | 69(54 to 89) | 83.64(65.73 to 111.76) | 142(108 to 180) | 47.19(36.21 to 59.15) | -2.14 (-2.33 to -1.96) | -1.92 (-2.1 to -1.74) | <0.001 |
| Mali | 2566(1951 to 3302) | 72.03(56.16 to 91.43) | 6185(4615 to 8132) | 79.83(60.51 to 103.43) | 0.45 (0.38 to 0.51) | 0.37 (0.21 to 0.53) | <0.001 |
| Malta | 149(132 to 164) | 37.55(33.13 to 41.55) | 291(236 to 335) | 26.18(21.42 to 30.09) | -1.11 (-1.32 to -0.9) | -1.35 (-2.42 to -0.27) | 0.014 |
| Marshall Islands | 21(16 to 29) | 140.86(103.09 to 190.46) | 70(44 to 112) | 221.34(139.82 to 353.08) | 1.42 (1.16 to 1.67) | 1.48 (1.35 to 1.61) | <0.001 |
| Mauritania | 671(499 to 872) | 73.4(54.99 to 94.94) | 1478(1037 to 2053) | 77.81(55.04 to 106.95) | -0.09 (-0.21 to 0.04) | 0.2 (0.11 to 0.3) | <0.001 |
| Mauritius | 687(634 to 744) | 97.57(89.62 to 105.84) | 3341(3011 to 3588) | 186.61(167.75 to 201.01) | 3.03 (2.36 to 3.71) | 1.82 (0.93 to 2.71) | <0.001 |
| Mexico | 42902(41323 to 44307) | 109.43(104.82 to 113.23) | 152123(134180 to 171558) | 123.73(109.22 to 139.28) | 0.6 (0.45 to 0.75) | 0.27 (-0.05 to 0.6) | 0.1 |
| Micronesia (Federated States of) | 56(41 to 76) | 121.41(90.33 to 164.24) | 116(85 to 159) | 175.38(129.18 to 237.5) | 1.15 (0.85 to 1.46) | 1.21 (1.14 to 1.27) | <0.001 |
| Monaco | 8(6 to 10) | 9.77(7.12 to 12.64) | 16(12 to 21) | 13.3(9.88 to 17.19) | 1.17 (0.98 to 1.36) | 1.02 (0.9 to 1.13) | <0.001 |
| Mongolia | 251(201 to 316) | 22.33(17.88 to 27.71) | 513(408 to 633) | 22.95(18.08 to 28.32) | -0.15 (-0.27 to -0.03) | 0.05 (-0.34 to 0.45) | 0.803 |
| Montenegro | 158(129 to 190) | 26.42(21.49 to 31.93) | 344(267 to 422) | 38.33(29.36 to 47.02) | 1.3 (1.12 to 1.48) | 1.38 (0.98 to 1.77) | <0.001 |
| Morocco | 4741(3277 to 7706) | 35.36(24.29 to 59.86) | 19483(14165 to 25490) | 63.89(46.8 to 83.41) | 2.24 (2.1 to 2.39) | 1.92 (1.79 to 2.05) | <0.001 |
| Mozambique | 3589(2702 to 4711) | 65.78(50.53 to 85.17) | 8790(6245 to 11974) | 86.29(62.12 to 114.69) | 1.35 (1.2 to 1.51) | 0.91 (0.71 to 1.11) | <0.001 |
| Myanmar | 19846(14332 to 26452) | 87.01(64.01 to 115.27) | 36754(27582 to 48219) | 82.25(61.98 to 107.55) | -0.45 (-0.54 to -0.36) | -0.18 (-0.26 to -0.1) | <0.001 |
| Namibia | 459(354 to 592) | 81.34(62.78 to 104.7) | 1171(845 to 1569) | 98.96(72.56 to 130.82) | 0.42 (0.11 to 0.73) | 0.66 (0.44 to 0.88) | <0.001 |
| Nauru | 7(5 to 9) | 159.81(117.23 to 215.14) | 11(8 to 16) | 210.45(153.54 to 289.52) | 0.75 (0.6 to 0.89) | 0.89 (0.82 to 0.97) | <0.001 |
| Nepal | 3019(2227 to 3965) | 35.5(26.52 to 46.42) | 10601(7728 to 14242) | 51.79(38.12 to 69.19) | 1.39 (1.1 to 1.68) | 1.23 (1.11 to 1.35) | <0.001 |
| Netherlands | 5176(4452 to 5762) | 25.55(21.93 to 28.46) | 7646(6343 to 8586) | 19.09(15.92 to 21.42) | -0.93 (-1.18 to -0.68) | -0.96 (-1.22 to -0.7) | <0.001 |
| New Zealand | 707(636 to 772) | 18.45(16.51 to 20.17) | 1615(1386 to 1779) | 18.12(15.65 to 19.93) | -0.42 (-0.7 to -0.13) | -0.02 (-0.48 to 0.43) | 0.921 |
| Nicaragua | 859(721 to 1051) | 54.08(45.34 to 66.04) | 4104(3228 to 5062) | 85.63(67.69 to 105.4) | 1.84 (1.52 to 2.17) | 1.49 (0.79 to 2.2) | <0.001 |
| Niger | 1444(1037 to 1950) | 52(38.6 to 69.31) | 3963(2800 to 5693) | 54.91(40 to 76.65) | 0.17 (0.11 to 0.24) | 0.19 (0.07 to 0.31) | 0.003 |
| Nigeria | 26895(21402 to 32840) | 66.62(54.5 to 80.06) | 54337(41542 to 69697) | 69.25(55.15 to 85.42) | 0.04 (-0.01 to 0.08) | 0.14 (0.02 to 0.25) | 0.02 |
| Niue | 3(2 to 3) | 113.36(85.14 to 147.47) | 4(3 to 5) | 178.56(128.64 to 236.23) | 1.34 (1.18 to 1.5) | 1.49 (1.31 to 1.66) | <0.001 |
| North Macedonia | 578(474 to 693) | 33.24(27.26 to 39.85) | 1343(1048 to 1679) | 46.85(36.63 to 58.39) | 1.13 (0.74 to 1.54) | 1.17 (0.83 to 1.52) | <0.001 |
| Northern Mariana Islands | 16(12 to 21) | 103.73(80.4 to 133.01) | 53(43 to 66) | 119.79(96.29 to 147.54) | 0.51 (0.33 to 0.69) | 0.36 (0.1 to 0.62) | 0.007 |
| Norway | 806(722 to 855) | 11.03(9.92 to 11.69) | 1438(1211 to 1571) | 12.06(10.33 to 13.1) | 0.26 (-0.11 to 0.63) | 0.14 (-0.44 to 0.72) | 0.636 |
| Oman | 387(276 to 545) | 63.65(45.49 to 88.71) | 1333(1006 to 1726) | 88.87(66.96 to 114.37) | 1.66 (1.39 to 1.92) | 1.12 (0.39 to 1.86) | 0.003 |
| Pakistan | 24801(19166 to 31221) | 47.35(36.59 to 59.62) | 78503(60530 to 99275) | 72.94(56.89 to 91.35) | 1.16 (0.9 to 1.41) | 1.39 (1.26 to 1.52) | <0.001 |
| Palau | 10(8 to 13) | 114.33(86.09 to 148.79) | 30(22 to 39) | 156.98(118.93 to 203.49) | 1.17 (1.01 to 1.34) | 1.02 (0.9 to 1.13) | <0.001 |
| Palestine | 759(568 to 999) | 99.77(75.28 to 130.91) | 1907(1541 to 2320) | 97.97(79.25 to 118.98) | -0.04 (-0.27 to 0.19) | -0.12 (-0.34 to 0.09) | 0.27 |
| Panama | 502(450 to 552) | 34.46(30.8 to 37.92) | 2642(2032 to 3206) | 58.87(45.37 to 71.43) | 1.72 (1.44 to 2.01) | 1.9 (1.64 to 2.15) | <0.001 |
| Papua New Guinea | 1702(1122 to 2392) | 101.78(69.1 to 141.71) | 4834(3498 to 6465) | 103.6(75.46 to 138.32) | 0.05 (0.02 to 0.09) | 0.07 (-0.05 to 0.18) | 0.253 |
| Paraguay | 906(735 to 1093) | 42.1(34.09 to 50.77) | 4557(3378 to 5949) | 81.95(60.8 to 106.68) | 2.46 (2.21 to 2.71) | 2.2 (1.76 to 2.63) | <0.001 |
| Peru | 4891(3963 to 5932) | 41.33(33.52 to 50.07) | 16313(11813 to 21309) | 48.77(35.37 to 63.64) | 0.3 (0.08 to 0.52) | 0.64 (-0.52 to 1.81) | 0.283 |
| Philippines | 16621(14916 to 18573) | 60.9(54 to 68.16) | 63607(53533 to 73733) | 81.54(68.84 to 94.31) | 1.28 (1.2 to 1.37) | 0.97 (0.7 to 1.24) | <0.001 |
| Poland | 11558(11071 to 11956) | 26.99(25.75 to 27.96) | 15232(13354 to 16794) | 20.32(17.86 to 22.45) | -1 (-1.23 to -0.77) | -0.87 (-1.19 to -0.55) | <0.001 |
| Portugal | 4661(4206 to 5095) | 35.49(31.83 to 38.82) | 8709(7181 to 9777) | 27.46(22.94 to 30.76) | -1.09 (-1.44 to -0.74) | -0.84 (-1.32 to -0.36) | 0.001 |
| Puerto Rico | 3040(2794 to 3278) | 87.84(80.5 to 94.82) | 5793(4673 to 6901) | 74.04(60.17 to 88.22) | -0.59 (-0.69 to -0.49) | -0.58 (-0.69 to -0.48) | <0.001 |
| Qatar | 85(62 to 116) | 126.48(93.42 to 170.23) | 580(428 to 767) | 117.01(88.72 to 149.57) | -0.49 (-1.25 to 0.27) | -0.21 (-1.41 to 1.01) | 0.735 |
| Republic of Korea | 10226(8541 to 12075) | 40.36(33.3 to 48.02) | 20539(16114 to 24776) | 22.41(17.53 to 27.08) | -2.16 (-2.46 to -1.87) | -1.91 (-2.22 to -1.6) | <0.001 |
| Republic of Moldova | 402(361 to 445) | 9.32(8.36 to 10.3) | 764(660 to 877) | 13.17(11.38 to 15.11) | 0.19 (-0.48 to 0.87) | 1.04 (0.04 to 2.06) | 0.042 |
| Romania | 4686(4291 to 5093) | 18.26(16.69 to 19.84) | 6108(5250 to 7007) | 15.79(13.53 to 18.21) | -0.29 (-0.72 to 0.14) | -0.44 (-0.76 to -0.13) | 0.006 |
| Russian Federation | 17327(16794 to 17808) | 9.92(9.59 to 10.21) | 53855(48534 to 58859) | 22.47(20.26 to 24.55) | 1.72 (0.67 to 2.78) | 2.9 (1.57 to 4.24) | <0.001 |
| Rwanda | 2817(2106 to 3630) | 106.59(79.99 to 136.87) | 4308(2919 to 6026) | 81.24(55.62 to 112.09) | -1.76 (-2.09 to -1.42) | -0.87 (-1.01 to -0.73) | <0.001 |
| Saint Kitts and Nevis | 44(40 to 49) | 118.71(107.32 to 130.45) | 65(52 to 78) | 110.14(88.75 to 130.82) | 0.33 (0.15 to 0.52) | -0.22 (-0.58 to 0.14) | 0.233 |
| Saint Lucia | 111(101 to 121) | 137.16(125.16 to 149.52) | 229(187 to 273) | 98.66(80.61 to 117.73) | -1.69 (-1.95 to -1.44) | -1.19 (-1.93 to -0.44) | 0.002 |
| Saint Vincent and the Grenadines | 88(79 to 97) | 127.85(114.3 to 141.16) | 151(131 to 173) | 113.02(98.34 to 129.06) | -0.58 (-0.8 to -0.36) | -0.48 (-1.41 to 0.46) | 0.316 |
| Samoa | 84(64 to 110) | 107.18(82.1 to 139.25) | 183(141 to 236) | 136.22(104.96 to 174.17) | 0.7 (0.6 to 0.79) | 0.79 (0.71 to 0.86) | <0.001 |
| San Marino | 5(4 to 6) | 13.71(10.62 to 17.29) | 8(5 to 11) | 7.81(4.98 to 11.1) | -0.8 (-1.16 to -0.43) | -2.15 (-2.6 to -1.69) | <0.001 |
| Sao Tome and Principe | 42(32 to 53) | 67.03(51.85 to 84.35) | 88(60 to 115) | 90.89(62.49 to 116.48) | 1.06 (0.98 to 1.15) | 0.97 (0.75 to 1.2) | <0.001 |
| Saudi Arabia | 3655(2632 to 4954) | 68.75(50.17 to 93.2) | 17278(12777 to 22512) | 108.87(81.7 to 137.83) | 1.3 (1.15 to 1.45) | 1.5 (1.28 to 1.73) | <0.001 |
| Senegal | 2237(1711 to 2859) | 72.49(56.18 to 91.67) | 6154(4497 to 8374) | 89.95(66.34 to 120.48) | 0.66 (0.6 to 0.72) | 0.67 (0.6 to 0.73) | <0.001 |
| Serbia | 3659(3002 to 4438) | 39.71(32.49 to 48.29) | 6598(5268 to 8030) | 38.19(30.43 to 46.57) | -0.21 (-0.33 to -0.09) | -0.04 (-0.42 to 0.34) | 0.828 |
| Seychelles | 26(21 to 32) | 46.18(37.65 to 55.52) | 76(61 to 93) | 71.82(57.33 to 87.34) | 1.66 (1.43 to 1.89) | 1.34 (0.82 to 1.85) | <0.001 |
| Sierra Leone | 1102(797 to 1459) | 54.95(40.94 to 71.66) | 2163(1567 to 2981) | 62.21(46.14 to 83.79) | 0.5 (0.37 to 0.63) | 0.4 (0.32 to 0.48) | <0.001 |
| Singapore | 625(568 to 682) | 31.69(28.59 to 34.71) | 1001(840 to 1139) | 12.21(10.23 to 13.9) | -2.84 (-3.08 to -2.6) | -3.1 (-3.98 to -2.21) | <0.001 |
| Slovakia | 1368(1136 to 1631) | 23.1(19.15 to 27.56) | 1706(1346 to 2125) | 17.97(14.16 to 22.37) | -0.76 (-0.82 to -0.7) | -0.85 (-1.24 to -0.45) | <0.001 |
| Slovenia | 441(394 to 488) | 18.08(16.1 to 20.14) | 748(608 to 895) | 14.64(11.94 to 17.58) | -1.74 (-2.12 to -1.36) | -0.62 (-1.53 to 0.31) | 0.192 |
| Solomon Islands | 138(79 to 203) | 107.08(67 to 152.82) | 457(331 to 620) | 137.37(101.63 to 182.55) | 0.83 (0.78 to 0.88) | 0.8 (0.59 to 1.01) | <0.001 |
| Somalia | 2195(1532 to 3029) | 98.66(70.98 to 132.55) | 5763(4036 to 8024) | 103.16(73.91 to 138.66) | 0.25 (0.16 to 0.34) | 0.16 (0.04 to 0.27) | 0.007 |
| South Africa | 12392(10869 to 14093) | 62.95(55.12 to 71.65) | 45954(41461 to 50474) | 111.05(99.79 to 122.05) | 2.09 (1.65 to 2.53) | 1.8 (1.3 to 2.32) | <0.001 |
| South Sudan | 2122(1502 to 2908) | 88.03(63.44 to 119.18) | 3892(2681 to 5359) | 112.52(78.51 to 152.3) | 0.72 (0.56 to 0.87) | 0.8 (0.7 to 0.9) | <0.001 |
| Spain | 16813(14798 to 18494) | 31.36(27.43 to 34.53) | 24004(19215 to 27340) | 17.59(14.33 to 19.96) | -1.74 (-1.81 to -1.66) | -1.76 (-1.93 to -1.6) | <0.001 |
| Sri Lanka | 6120(5009 to 7417) | 66.63(54.66 to 80.54) | 17362(11477 to 23706) | 68.9(45.94 to 93.62) | 0.63 (0.4 to 0.85) | 0.11 (-0.48 to 0.71) | 0.712 |
| Sudan | 3195(2169 to 5229) | 35.43(24.46 to 60.45) | 8916(6322 to 12277) | 50.53(36.59 to 69.14) | 1.3 (1.12 to 1.48) | 1.16 (1.06 to 1.26) | <0.001 |
| Suriname | 170(141 to 201) | 68.3(56.75 to 80.53) | 518(382 to 676) | 84.12(61.94 to 109.95) | 0.97 (0.78 to 1.16) | 0.78 (0.25 to 1.31) | 0.004 |
| Sweden | 2192(1922 to 2403) | 13.57(11.9 to 14.88) | 4512(3690 to 5161) | 16.55(13.69 to 18.91) | 0.94 (0.8 to 1.08) | 0.61 (-0.04 to 1.26) | 0.066 |
| Switzerland | 2414(2107 to 2669) | 21.46(18.73 to 23.72) | 3811(2988 to 4395) | 15.59(12.46 to 17.94) | -0.85 (-0.94 to -0.76) | -0.87 (-1.51 to -0.23) | 0.008 |
| Syrian Arab Republic | 2838(2107 to 3785) | 57.81(43.44 to 77.15) | 7040(4991 to 9551) | 67.83(47.4 to 91.37) | 0.16 (-0.06 to 0.38) | 0.53 (0.25 to 0.81) | <0.001 |
| Taiwan (Province of China) | 8246(7551 to 8933) | 60.9(55.13 to 66.16) | 19472(16557 to 21896) | 44.62(38.18 to 50.05) | -1.53 (-1.82 to -1.25) | -0.93 (-1.46 to -0.4) | 0.001 |
| Tajikistan | 430(345 to 530) | 14.71(11.85 to 18.07) | 1001(750 to 1335) | 16.78(12.63 to 22.09) | 0.06 (-0.27 to 0.39) | 0.4 (-0.02 to 0.83) | 0.065 |
| Thailand | 16073(12525 to 20550) | 48.66(37.8 to 62.13) | 56347(41915 to 73604) | 52.7(39.24 to 68.84) | -0.31 (-0.53 to -0.09) | 0.26 (0.01 to 0.5) | 0.041 |
| Timor-Leste | 129(89 to 177) | 45.4(33.22 to 60.99) | 423(303 to 588) | 52.66(37.94 to 72.77) | 0.67 (0.46 to 0.88) | 0.45 (0.26 to 0.65) | <0.001 |
| Togo | 660(501 to 858) | 56.07(43.56 to 72.15) | 2272(1623 to 3124) | 70.46(51.73 to 94.53) | 0.66 (0.54 to 0.79) | 0.75 (0.61 to 0.89) | <0.001 |
| Tokelau | 1(1 to 2) | 100.96(73.09 to 139.98) | 2(1 to 2) | 121.91(90.31 to 163.16) | 0.53 (0.41 to 0.64) | 0.62 (0.56 to 0.68) | <0.001 |
| Tonga | 54(42 to 69) | 104.75(80.56 to 133.01) | 103(78 to 135) | 132.23(99.5 to 172.41) | 0.74 (0.58 to 0.91) | 0.78 (0.55 to 1.02) | <0.001 |
| Trinidad and Tobago | 1235(1134 to 1336) | 157.1(143.86 to 169.93) | 2626(1981 to 3315) | 138.14(104.3 to 174.14) | -0.53 (-0.66 to -0.39) | -0.25 (-0.62 to 0.13) | 0.204 |
| Tunisia | 1395(1039 to 1988) | 32.94(24.46 to 46.85) | 5846(4045 to 8261) | 48.91(33.94 to 69.54) | 1.35 (1.27 to 1.43) | 1.23 (1 to 1.45) | <0.001 |
| Turkey | 20340(15919 to 26101) | 67.17(52.69 to 86.12) | 47788(36614 to 60100) | 56.11(42.96 to 70.4) | -0.16 (-0.59 to 0.27) | -0.52 (-1.17 to 0.14) | 0.125 |
| Turkmenistan | 430(393 to 472) | 20.04(18.29 to 21.93) | 1667(1285 to 2146) | 40.04(31.04 to 51.19) | 1.68 (1.34 to 2.03) | 2.49 (1.8 to 3.18) | <0.001 |
| Tuvalu | 7(5 to 10) | 115.33(85.1 to 157.32) | 14(10 to 18) | 141.32(107.54 to 187.72) | 0.69 (0.6 to 0.79) | 0.67 (0.6 to 0.74) | <0.001 |
| Uganda | 4272(3020 to 5987) | 72.95(51.91 to 101.47) | 11276(8029 to 15536) | 85.86(62.2 to 116.66) | 0.17 (-0.03 to 0.36) | 0.52 (0.37 to 0.67) | <0.001 |
| Ukraine | 2979(2651 to 3324) | 4.27(3.81 to 4.76) | 4090(3011 to 5290) | 5.75(4.21 to 7.46) | 0.09 (-0.32 to 0.49) | 1.23 (-0.01 to 2.48) | 0.052 |
| United Arab Emirates | 225(161 to 314) | 65.36(47.47 to 88.84) | 1499(1043 to 1981) | 80.63(57.08 to 103.56) | 2.76 (2.05 to 3.48) | 0.66 (-1.66 to 3.04) | 0.578 |
| United Kingdom | 12917(11949 to 13423) | 13.86(12.79 to 14.42) | 16240(13959 to 17536) | 10.73(9.35 to 11.55) | -0.47 (-0.8 to -0.15) | -0.86 (-1.38 to -0.34) | 0.001 |
| United Republic of Tanzania | 7410(5756 to 9455) | 74.15(58.18 to 93.81) | 17635(13161 to 23395) | 76.08(57.62 to 99.75) | -0.03 (-0.08 to 0.02) | 0.09 (-0.01 to 0.19) | 0.076 |
| United States of America | 77387(70364 to 81221) | 23.76(21.67 to 24.91) | 210501(184160 to 226279) | 34.67(30.67 to 37.12) | 1.05 (0.84 to 1.26) | 1.28 (1.1 to 1.46) | <0.001 |
| United States Virgin Islands | 49(38 to 60) | 65.64(51.82 to 81.29) | 85(62 to 112) | 50.07(36.35 to 65.95) | -0.73 (-0.92 to -0.54) | -0.92 (-1.3 to -0.54) | <0.001 |
| Uruguay | 1270(1156 to 1381) | 32.45(29.43 to 35.28) | 2141(1866 to 2363) | 34.08(29.98 to 37.52) | 0.16 (0.03 to 0.28) | 0.19 (-0.28 to 0.66) | 0.428 |
| Uzbekistan | 1863(1585 to 2267) | 15.42(13.04 to 18.97) | 10322(8548 to 12205) | 39.52(32.8 to 46.56) | 2.5 (1.97 to 3.03) | 2.93 (2.04 to 3.83) | <0.001 |
| Vanuatu | 53(37 to 80) | 93.98(67.08 to 138.64) | 197(149 to 262) | 124.19(94.68 to 163.77) | 0.85 (0.8 to 0.91) | 0.89 (0.7 to 1.08) | <0.001 |
| Venezuela (Bolivarian Republic of) | 4746(4320 to 5152) | 50.78(45.91 to 55.24) | 24366(18399 to 31371) | 84.29(63.91 to 108.09) | 1.41 (1.23 to 1.6) | 1.57 (1.02 to 2.12) | <0.001 |
| Viet Nam | 20680(15394 to 27333) | 54.23(40.76 to 71.46) | 55063(40950 to 71037) | 63.14(47.18 to 81.02) | 0.63 (0.56 to 0.71) | 0.51 (0.45 to 0.57) | <0.001 |
| Yemen | 1450(947 to 2475) | 31.55(21.15 to 55.19) | 4364(2889 to 6972) | 35.59(23.97 to 56.41) | 0.4 (0.29 to 0.51) | 0.39 (0.27 to 0.51) | <0.001 |
| Zambia | 2579(2005 to 3255) | 95.38(74.9 to 120.14) | 6336(4475 to 8795) | 99.36(72.9 to 132.24) | -0.24 (-0.41 to -0.07) | 0.16 (0 to 0.33) | 0.044 |
| Zimbabwe | 2184(1671 to 2820) | 63.4(48.59 to 81.45) | 6469(4815 to 8572) | 107.68(81 to 140.16) | 2.27 (1.73 to 2.81) | 1.82 (1.18 to 2.46) | <0.001 |

*ASR: age-standardized rate; EAPC: estimated annual percentage change; AAPC: average annual percent change; SDI: sociodemographic index; UI: uncertainty interval; CI: confidence interval.*

**Table S8. Changes in prevalence of diabetes and kidney diseases according to population-level determinants including aging, population growth and epidemiological change from 1990 to 2021.**

| **location** | **Overll difference** | **Aging** | **Population** | **Epidemiological change** | **percent change of Aging** | **percent change of Population** | **percent change of Epidemiological change** |
| --- | --- | --- | --- | --- | --- | --- | --- |
| Global | 503251997 | 157630743 | 216596071.9 | 129025182.1 | 31.32242771 | 43.03928711 | 25.63828518 |
| **SDI** |  |  |  |  |  |  |  |
| High SDI | 93207967.8 | 33246756.6 | 24705710.37 | 35255500.82 | 35.6694362 | 26.5060069 | 37.82455691 |
| High-middle SDI | 83233528.05 | 38687345.46 | 21428999.85 | 23117182.74 | 46.48048253 | 25.74563442 | 27.77388305 |
| Middle SDI | 169444261.8 | 73734539.33 | 60562680.83 | 35147041.64 | 43.51551274 | 35.74194852 | 20.74253874 |
| Low-middle SDI | 113822986 | 34463011.15 | 55800505.08 | 23559469.76 | 30.27772541 | 49.02393361 | 20.69834098 |
| Low SDI | 43178595.34 | 4314477.724 | 32128285.83 | 6735831.786 | 9.992167855 | 74.40789951 | 15.59993263 |
| **Region** |  |  |  |  |  |  |  |
| Andean Latin America | 3593859.013 | 1174820.973 | 1569584.148 | 849453.892 | 32.68967895 | 43.67406017 | 23.63626088 |
| Australasia | 1953378.574 | 611712.9121 | 972774.2172 | 368891.4442 | 31.3156354 | 49.79957446 | 18.88479014 |
| Caribbean | 3339822.448 | 1228798.715 | 982641.6003 | 1128382.132 | 36.79233655 | 29.42197124 | 33.7856922 |
| Central Asia | 5507104.922 | 1673692.595 | 2520948.858 | 1312463.469 | 30.39151458 | 45.77629977 | 23.83218565 |
| Central Europe | 5025674.715 | 4281336.709 | -1077497.134 | 1821835.14 | 85.18929203 | -21.43985028 | 36.25055825 |
| Central Latin America | 19631411.94 | 9805588.516 | 6927663.123 | 2898160.3 | 49.94846293 | 35.28866464 | 14.76287243 |
| Central Sub-Saharan Africa | 5699015.909 | 371972.1175 | 4383267.717 | 943776.0736 | 6.526953487 | 76.91271244 | 16.56033408 |
| East Asia | 100868029.5 | 53451730.66 | 21476582.07 | 25939716.77 | 52.99174667 | 21.29176328 | 25.71649005 |
| Eastern Europe | 5800112.418 | 4925574.344 | -2276207.55 | 3150745.624 | 84.92204958 | -39.2441971 | 54.32214752 |
| Eastern Sub-Saharan Africa | 11136894.18 | 1484055.142 | 8106023.392 | 1546815.643 | 13.3255746 | 72.78531396 | 13.88911144 |
| High-income Asia Pacific | 17904166.43 | 11503080.46 | 1174478.869 | 5226607.1 | 64.24806487 | 6.559807594 | 29.19212754 |
| High-income North America | 37680541.76 | 12019290.66 | 10564489.65 | 15096761.45 | 31.89787115 | 28.0369898 | 40.06513905 |
| North Africa and Middle East | 52725764.76 | 14479505.55 | 23503530.01 | 14742729.21 | 27.4619166 | 44.57693523 | 27.96114816 |
| Oceania | 1095611.988 | 151304.509 | 628254.4585 | 316053.0202 | 13.81004504 | 57.34278792 | 28.84716704 |
| South Asia | 118161972.9 | 40655198.85 | 60268631.94 | 17238142.11 | 34.40633044 | 51.00509958 | 14.58856998 |
| Southeast Asia | 47888456.99 | 19336798.01 | 19112481.2 | 9439177.773 | 40.3788287 | 39.9104135 | 19.7107578 |
| Southern Latin America | 4056777.811 | 1251452.221 | 1405265.75 | 1400059.84 | 30.84842895 | 34.63994864 | 34.51162241 |
| Southern Sub-Saharan Africa | 3756092.839 | 1330099.914 | 1610427.834 | 815565.0909 | 35.41179547 | 42.87508065 | 21.71312387 |
| Tropical Latin America | 14556932.63 | 7577619.375 | 5512481.184 | 1466832.069 | 52.05505562 | 37.86842548 | 10.07651891 |
| Western Europe | 25167323.69 | 9520423.655 | 5630693.618 | 10016206.42 | 37.82851038 | 22.37303293 | 39.79845669 |
| Western Sub-Saharan Africa | 17703051.58 | -129944.9089 | 14766901.46 | 3066095.022 | -0.734025478 | 83.41444072 | 17.31958476 |

*SDI: sociodemographic index.*

**Table S9. Changes in incidence of diabetes and kidney diseases according to population-level determinants including aging, population growth and epidemiological change from 1990 to 2021.**

| **location** | **Overll difference** | **Aging** | **Population** | **Epidemiological change** | **percent change of Aging** | **percent change of Population** | **percent change of Epidemiological change** |
| --- | --- | --- | --- | --- | --- | --- | --- |
| Global | 23322969.74 | 6680199.511 | 8609484.584 | 8033285.651 | 28.64214799 | 36.91418665 | 34.44366536 |
| **SDI** |  |  |  |  |  |  |  |
| High SDI | 4984837.536 | 1737472.802 | 1249409.771 | 1997954.963 | 34.85515404 | 25.06420243 | 40.08064353 |
| High-middle SDI | 3665754.524 | 1553561.789 | 813645.9511 | 1298546.784 | 42.3804098 | 22.1958657 | 35.4237245 |
| Middle SDI | 7876405.467 | 2995296.743 | 2342777.995 | 2538330.73 | 38.02872713 | 29.74425332 | 32.22701955 |
| Low-middle SDI | 5030495.3 | 1141304.061 | 1938964.173 | 1950227.065 | 22.68770753 | 38.54420008 | 38.76809239 |
| Low SDI | 1744672.655 | 105723.77 | 1074454.188 | 564494.6968 | 6.059805526 | 61.58485864 | 32.35533584 |
| **Region** |  |  |  |  |  |  |  |
| Andean Latin America | 237758.5964 | 63607.12568 | 80918.11931 | 93233.35141 | 26.75281846 | 34.03373024 | 39.2134513 |
| Australasia | 126640.4315 | 38880.39266 | 55038.43153 | 32721.60727 | 30.70140571 | 43.46039483 | 25.83819945 |
| Caribbean | 194034.7618 | 57617.62017 | 48190.28009 | 88226.8615 | 29.69448343 | 24.83590036 | 45.46961622 |
| Central Asia | 257754.1628 | 51959.45477 | 75859.36071 | 129935.3473 | 20.15853176 | 29.43089643 | 50.41057181 |
| Central Europe | 317813.6948 | 165918.9587 | -48541.76389 | 200436.4999 | 52.2063591 | -15.27365393 | 63.06729483 |
| Central Latin America | 1181797.05 | 501878.2476 | 370389.9471 | 309528.8555 | 42.46738029 | 31.34124823 | 26.19137148 |
| Central Sub-Saharan Africa | 220644.9185 | 8534.262877 | 135668.1341 | 76442.52154 | 3.867871934 | 61.4870875 | 34.64504057 |
| East Asia | 3354614.243 | 1873175.015 | 759988.4657 | 721450.7619 | 55.83876057 | 22.65501816 | 21.50622127 |
| Eastern Europe | 390173.1982 | 125743.8225 | -64760.98109 | 329190.3568 | 32.22769351 | -16.59800862 | 84.37031511 |
| Eastern Sub-Saharan Africa | 400785.2872 | 26658.01979 | 272354.5612 | 101772.7062 | 6.651446707 | 67.95522936 | 25.39332393 |
| High-income Asia Pacific | 896366.1644 | 538030.3594 | 54622.90072 | 303712.9042 | 60.02350165 | 6.093815551 | 33.8826828 |
| High-income North America | 2135366.133 | 663207.5794 | 563417.553 | 908741.0005 | 31.05826065 | 26.38505614 | 42.55668321 |
| North Africa and Middle East | 3340433.554 | 768822.9335 | 1170584.542 | 1401026.078 | 23.01566312 | 35.04289259 | 41.94144429 |
| Oceania | 52458.81985 | 5738.956468 | 28628.43177 | 18091.43161 | 10.93992675 | 54.57315252 | 34.48692072 |
| South Asia | 4471485.906 | 1176323.251 | 1864301.152 | 1430861.502 | 26.30721143 | 41.69310139 | 31.99968718 |
| Southeast Asia | 2411333.394 | 827683.0434 | 687030.9078 | 896619.4428 | 34.32470373 | 28.49174277 | 37.18355351 |
| Southern Latin America | 250109.0427 | 71247.83675 | 76174.34077 | 102686.8652 | 28.48670963 | 30.45645209 | 41.05683828 |
| Southern Sub-Saharan Africa | 206321.3885 | 55244.62142 | 63874.30641 | 87202.46063 | 26.77600312 | 30.9586451 | 42.26535178 |
| Tropical Latin America | 807781.0313 | 340522.703 | 254109.611 | 213148.7173 | 42.15532302 | 31.45773436 | 26.38694263 |
| Western Europe | 1329243.989 | 563633.4785 | 296385.4954 | 469225.015 | 42.40255989 | 22.29729816 | 35.30014195 |
| Western Sub-Saharan Africa | 740053.9784 | -13324.6998 | 514304.9625 | 239073.7157 | -1.800503772 | 69.49560133 | 32.30490244 |

*SDI: sociodemographic index.*

**Table S10. Changes in DALYs of diabetes and kidney diseases according to population-level determinants including aging, population growth and epidemiological change from 1990 to 2021.**

| **location** | **Overll difference** | **Aging** | **Population** | **Epidemiological change** | **percent change of Aging** | **percent change of Population** | **percent change of Epidemiological change** |
| --- | --- | --- | --- | --- | --- | --- | --- |
| Global | 60596640.67 | 22522929.11 | 24255940.49 | 13817771.07 | 37.1686101 | 40.0285234 | 22.80286649 |
| **SDI** |  |  |  |  |  |  |  |
| High SDI | 9419243.074 | 4011295.748 | 2336248.157 | 3071699.169 | 42.58617934 | 24.80292884 | 32.61089182 |
| High-middle SDI | 7554824.289 | 4649225.887 | 1900417.379 | 1005181.024 | 61.53982818 | 25.15501759 | 13.30515424 |
| Middle SDI | 22060928.6 | 11764332.71 | 7203399.252 | 3093196.635 | 53.32655268 | 32.65229394 | 14.02115338 |
| Low-middle SDI | 15947444.92 | 5049451.589 | 6973130.009 | 3924863.323 | 31.6630759 | 43.72568799 | 24.61123611 |
| Low SDI | 5559657.921 | 497900.5277 | 5150359.461 | -88602.0681 | 8.955596456 | 92.63806397 | -1.593660426 |
| **Region** |  |  |  |  |  |  |  |
| Andean Latin America | 602626.2575 | 249437.8288 | 287594.6835 | 65593.74516 | 41.39179562 | 47.72355667 | 10.88464771 |
| Australasia | 166058.5187 | 66228.62217 | 81171.01776 | 18658.87879 | 39.8827008 | 48.88097183 | 11.23632737 |
| Caribbean | 581917.9259 | 265733.0291 | 176713.5398 | 139471.357 | 45.66503579 | 30.3674336 | 23.96753061 |
| Central Asia | 560548.5436 | 178648.1891 | 239068.9452 | 142831.4094 | 31.87024409 | 42.64910647 | 25.48064944 |
| Central Europe | 654897.8622 | 545418.5382 | -113650.832 | 223130.156 | 83.28299261 | -17.35397816 | 34.07098555 |
| Central Latin America | 4446412.036 | 2327108.602 | 1352940.149 | 766363.2846 | 52.33677364 | 30.42768277 | 17.23554359 |
| Central Sub-Saharan Africa | 923052.6128 | 52065.02023 | 850421.1888 | 20566.40372 | 5.640525741 | 92.13138851 | 2.228085749 |
| East Asia | 7627663.311 | 6457641.607 | 1887911.918 | -717890.2141 | 84.66081083 | 24.75085542 | -9.411666257 |
| Eastern Europe | 964594.8637 | 389063.2138 | -148797.351 | 724329.001 | 40.33436507 | -15.42589087 | 75.0915258 |
| Eastern Sub-Saharan Africa | 1692516.158 | 254402.9797 | 1963036.585 | -524923.4072 | 15.03105176 | 115.9833291 | -31.01438086 |
| High-income Asia Pacific | 1419817.585 | 1145808.217 | 87900.01947 | 186109.3477 | 80.70108652 | 6.190937514 | 13.10797597 |
| High-income North America | 4440182.848 | 1551496.963 | 1129080.322 | 1759605.563 | 34.9421863 | 25.42868979 | 39.62912391 |
| North Africa and Middle East | 6550527.038 | 1913382.304 | 2732550.97 | 1904593.764 | 29.20959325 | 41.71497887 | 29.07542787 |
| Oceania | 205233.9514 | 42978.00099 | 137625.4741 | 24630.47628 | 20.94098014 | 67.05784944 | 12.00117043 |
| South Asia | 14700319.61 | 5207404.016 | 6228705.206 | 3264210.392 | 35.42374692 | 42.371223 | 22.20503008 |
| Southeast Asia | 7200358.148 | 3415880.998 | 2631406.115 | 1153071.035 | 47.44043182 | 36.54548928 | 16.0140789 |
| Southern Latin America | 311705.9256 | 213231.1218 | 198230.8716 | -99756.0678 | 68.40778576 | 63.59547745 | -32.0032632 |
| Southern Sub-Saharan Africa | 923722.7796 | 317145.4067 | 323831.9126 | 282745.4603 | 34.33339674 | 35.05726174 | 30.60934152 |
| Tropical Latin America | 1910784.472 | 1312312.162 | 782415.973 | -183943.6624 | 68.67923519 | 40.94736923 | -9.626604413 |
| Western Europe | 2196758.02 | 1277977.841 | 518292.8225 | 400487.3563 | 58.17563108 | 23.59353273 | 18.2308362 |
| Western Sub-Saharan Africa | 2516942.198 | -115318.9765 | 2555793.579 | 76467.59602 | -4.581709366 | 101.5435945 | 3.038114903 |

*DALYs: disability-adjusted life years; SDI: sociodemographic index.*

**Table S11. Changes in deaths of diabetes and kidney diseases according to population-level determinants including aging, population growth and epidemiological change from 1990 to 2021.**

| **location** | **Overll difference** | **Aging** | **Population** | **Epidemiological change** | **percent change of Aging** | **percent change of Population** | **percent change of Epidemiological change** |
| --- | --- | --- | --- | --- | --- | --- | --- |
| Global | 1580927.192 | 787097.3329 | 624989.3711 | 168840.4878 | 49.78707033 | 39.5330901 | 10.67983957 |
| **SDI** |  |  |  |  |  |  |  |
| High SDI | 237421.6762 | 166170.6663 | 65291.32251 | 5959.687455 | 69.98967782 | 27.50015228 | 2.510169901 |
| High-middle SDI | 194907.0867 | 161182.8008 | 47474.42847 | -13750.14258 | 82.69725003 | 24.35746656 | -7.054716591 |
| Middle SDI | 597710.1893 | 391419.0572 | 183645.309 | 22645.82307 | 65.48642874 | 30.72480816 | 3.788763096 |
| Low-middle SDI | 418625.4669 | 161435.7865 | 177897.332 | 79292.34843 | 38.56329805 | 42.49558281 | 18.94111914 |
| Low SDI | 130839.7869 | 14775.26266 | 129741.623 | -13677.09876 | 11.29263736 | 99.16068046 | -10.45331782 |
| **Region** |  |  |  |  |  |  |  |
| Andean Latin America | 19830.87788 | 9832.310209 | 9123.741761 | 874.8259056 | 49.58081165 | 46.00775527 | 4.411433075 |
| Australasia | 5022.715046 | 3045.975315 | 2624.558859 | -647.8191272 | 60.64400004 | 52.25378774 | -12.89778777 |
| Caribbean | 14313.44122 | 8787.655174 | 4701.719578 | 824.0664657 | 61.39442668 | 32.8482823 | 5.757291019 |
| Central Asia | 9921.153112 | 3423.168413 | 4696.970801 | 1801.013898 | 34.50373534 | 47.34299278 | 18.15327188 |
| Central Europe | 19999.63255 | 19414.48 | -2852.387519 | 3437.540071 | 97.07418349 | -14.26219962 | 17.18801614 |
| Central Latin America | 133500.0367 | 79027.6863 | 38257.48599 | 16214.86439 | 59.1967525 | 28.657285 | 12.1459625 |
| Central Sub-Saharan Africa | 21629.02926 | 757.8859028 | 21717.89542 | -846.7520668 | 3.504021812 | 100.4108652 | -3.914887056 |
| East Asia | 172263.7236 | 206049.2135 | 41571.19771 | -75356.68769 | 119.6126551 | 24.13229951 | -43.74495462 |
| Eastern Europe | 34822.36072 | 10391.29408 | -3041.060274 | 27472.12692 | 29.84086622 | -8.733067523 | 78.89220131 |
| Eastern Sub-Saharan Africa | 47227.25612 | 8268.446766 | 54873.54964 | -15914.74029 | 17.50778564 | 116.1904251 | -33.69821073 |
| High-income Asia Pacific | 36021.35213 | 50424.27052 | 2178.445309 | -16581.3637 | 139.9843913 | 6.04765002 | -46.03204133 |
| High-income North America | 101753.2797 | 48583.5271 | 30855.69952 | 22314.0531 | 47.74639917 | 30.32403437 | 21.92956646 |
| North Africa and Middle East | 157720.4667 | 55538.76428 | 69551.06641 | 32630.63605 | 35.21341613 | 44.0976798 | 20.68890406 |
| Oceania | 4564.81168 | 1134.305002 | 3431.574159 | -1.067482183 | 24.84888933 | 75.17449569 | -0.023385021 |
| South Asia | 381287.9134 | 163987.9283 | 151379.0334 | 65920.9517 | 43.00895008 | 39.7020278 | 17.28902212 |
| Southeast Asia | 193924.3999 | 106218.6672 | 69294.46583 | 18411.2669 | 54.77323495 | 35.73272154 | 9.494043508 |
| Southern Latin America | 5730.210183 | 9197.277052 | 6935.113645 | -10402.18051 | 160.5050558 | 121.0272123 | -181.5322681 |
| Southern Sub-Saharan Africa | 27949.57939 | 10097.44766 | 10232.01193 | 7620.119811 | 36.12736891 | 36.60882256 | 27.26380853 |
| Tropical Latin America | 54866.45179 | 44454.51076 | 21511.6572 | -11099.71617 | 81.02311943 | 39.2073052 | -20.23042462 |
| Western Europe | 78989.24296 | 72224.48813 | 17880.21157 | -11115.45674 | 91.43585307 | 22.63626147 | -14.07211454 |
| Western Sub-Saharan Africa | 59589.25776 | -6465.363921 | 65954.94416 | 99.67751706 | -10.84988161 | 110.6826073 | 0.167274305 |

*SDI: sociodemographic index.*

**Figure S1. The joinpoint regression analysis on the top four regions with the highest ASR of AAPC of prevalence. ASR: age-standardized rate; AAPC: average annual percentage change.**

**
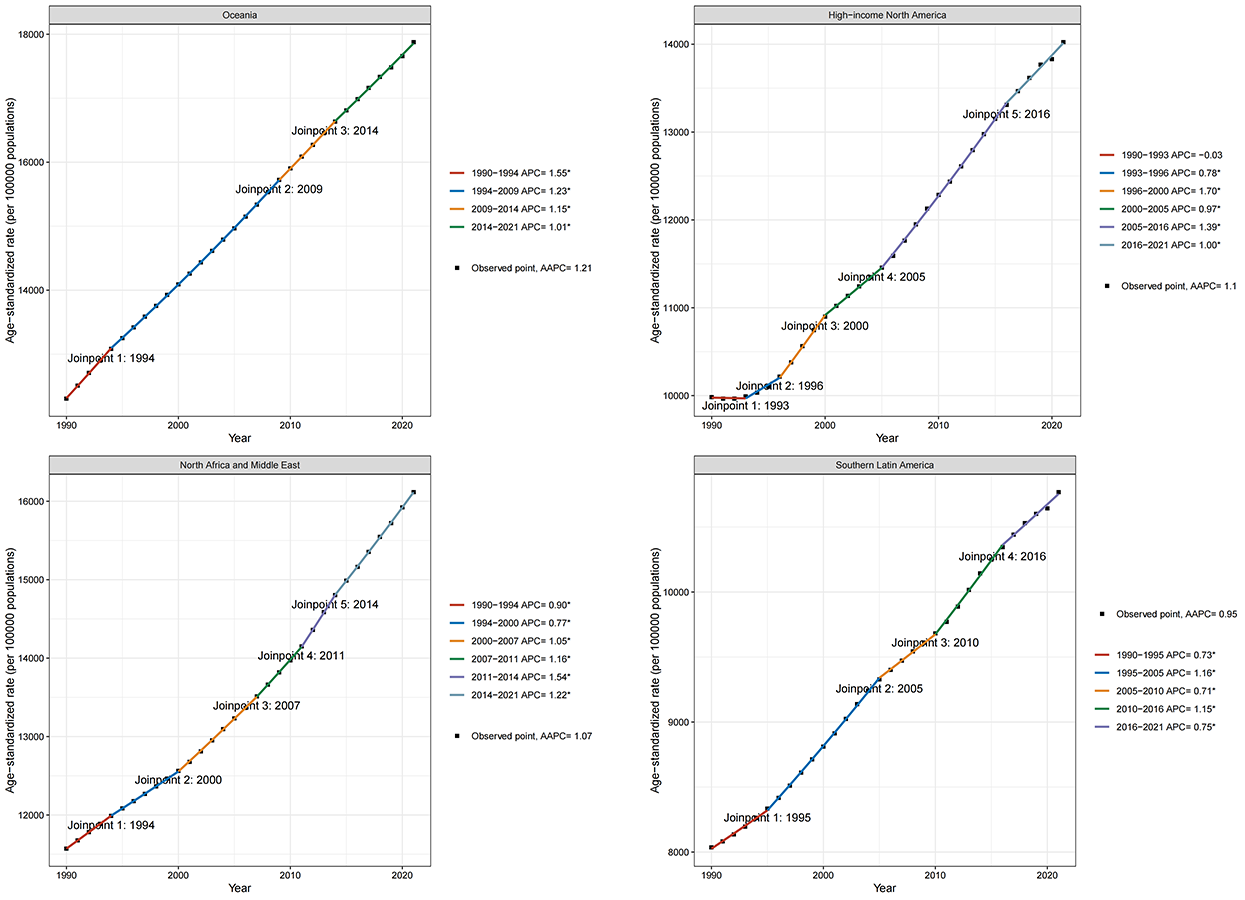
**

**Figure S2. The joinpoint regression analysis on the top four regions with the highest ASR of AAPC of incidence. ASR: age-standardized rate; AAPC: average annual percentage change.**

**
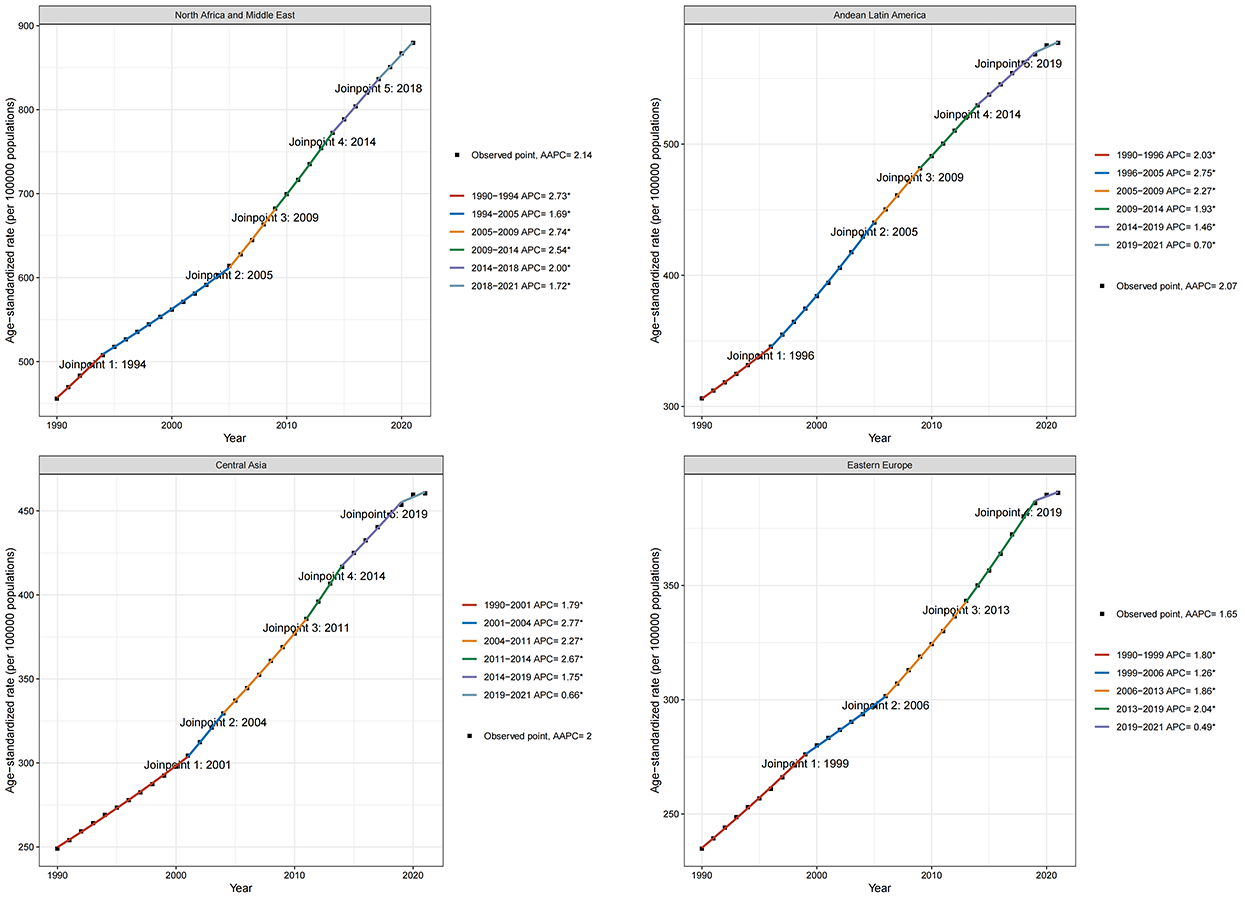
**

**Figure S3. The joinpoint regression analysis on the top four regions with the highest ASR of AAPC of DALYs. ASR: age-standardized rate; AAPC: average annual percentage change; DALYs: disability-adjusted life years.**

**
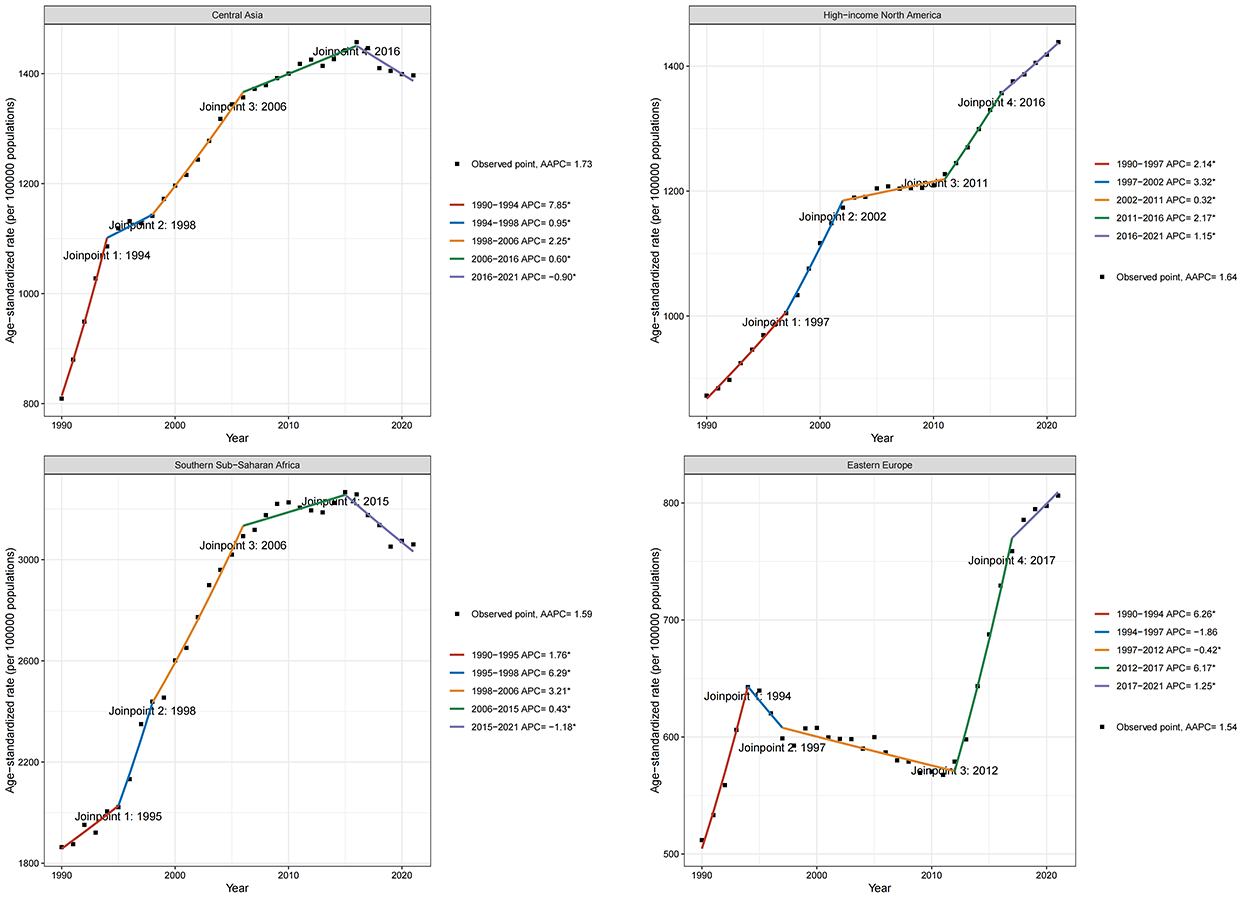
**

**Figure S4. The joinpoint regression analysis on the top four regions with the highest ASR of AAPC of mortality rate. ASR: age-standardized rate; AAPC: average annual percentage change.**

**
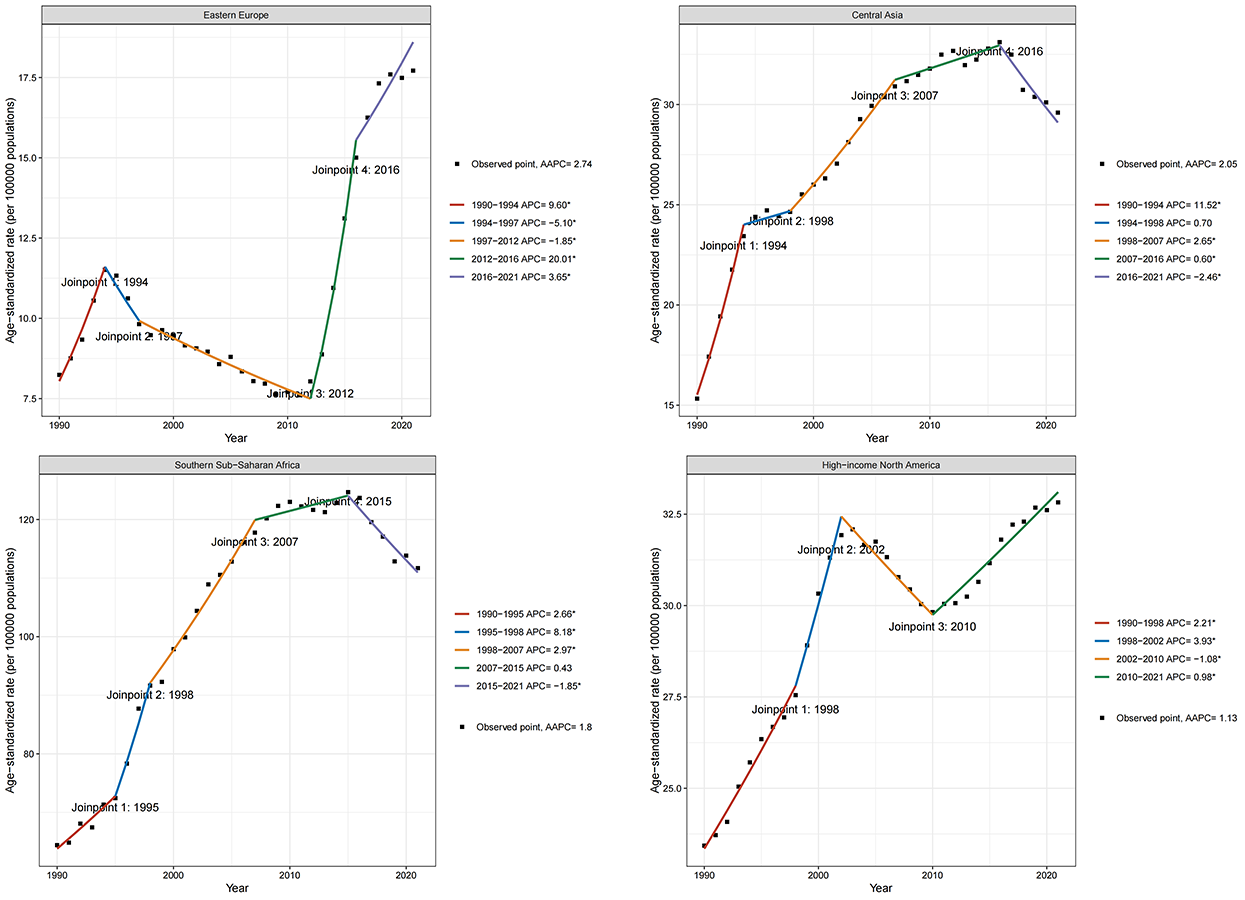
**

**Figure S5. The joinpoint regression analysis on the top four countries with the highest ASR of AAPC of prevalence. ASR: age-standardized rate; AAPC: average annual percentage change.**

**
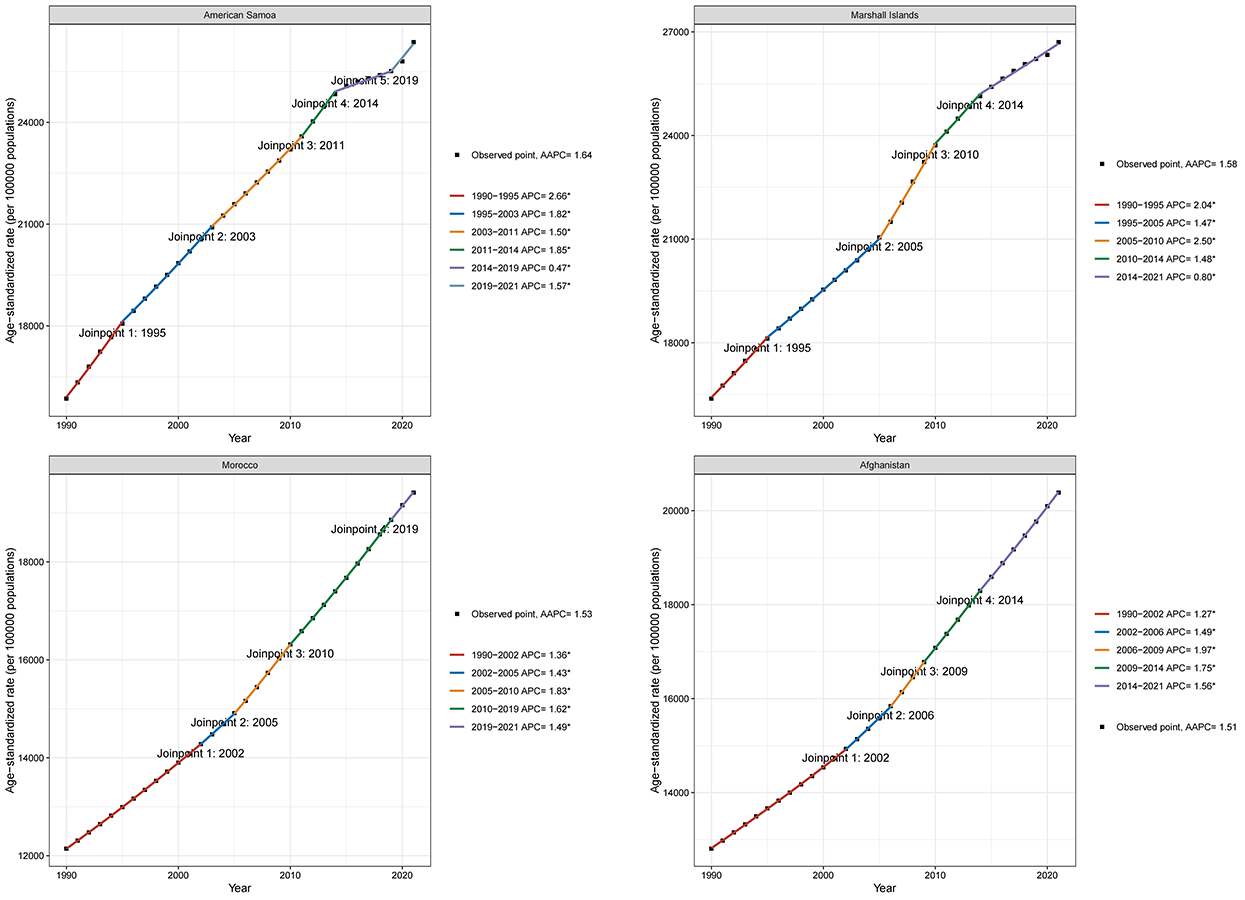
**

**Figure S6. The joinpoint regression analysis on the top four countries with the highest ASR of AAPC of incidence. ASR: age-standardized rate; AAPC: average annual percentage change.**

**
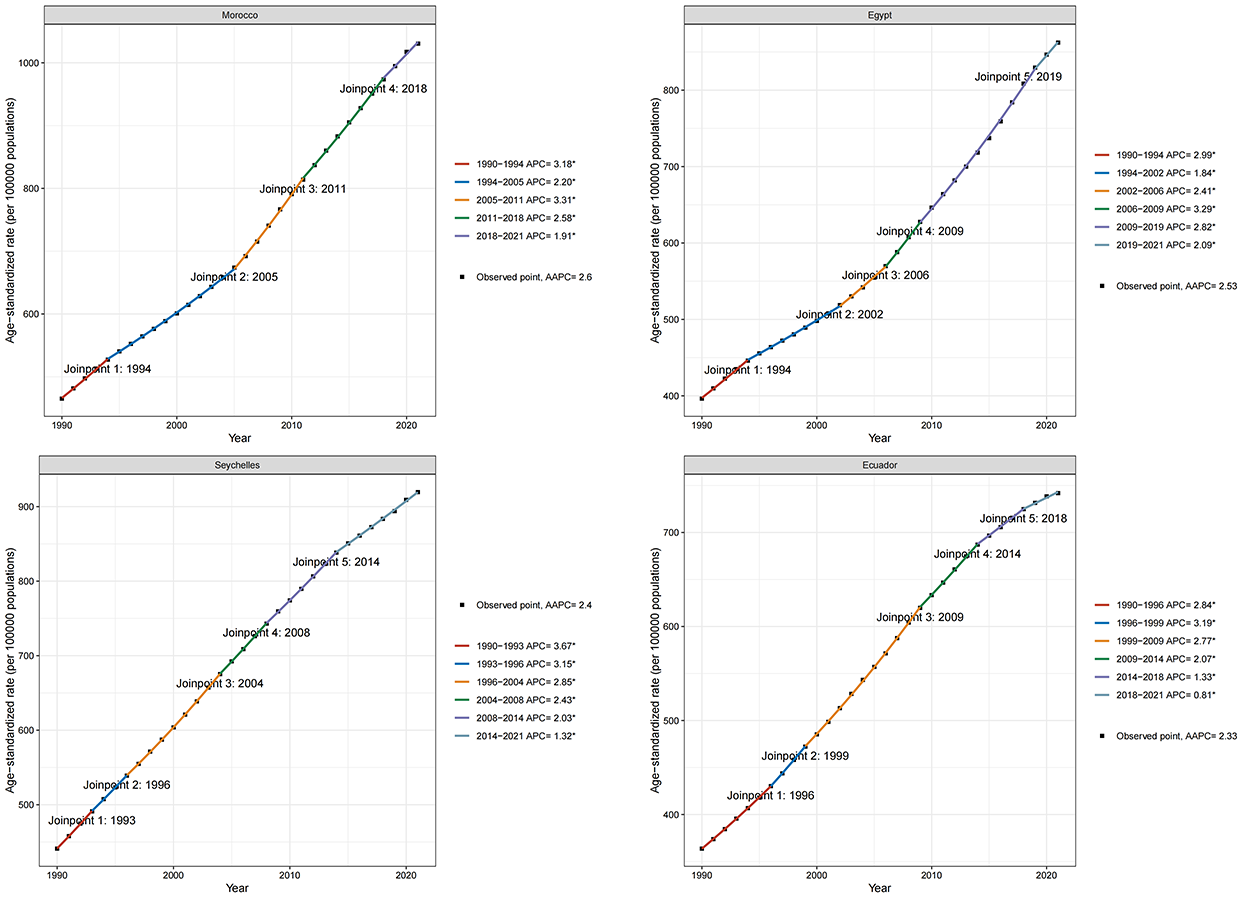
**

**Figure S7. The joinpoint regression analysis on the top four countries with the highest ASR of AAPC of DALYs. ASR: age-standardized rate; AAPC: average annual percentage change; DALYs: disability-adjusted life years.**

**
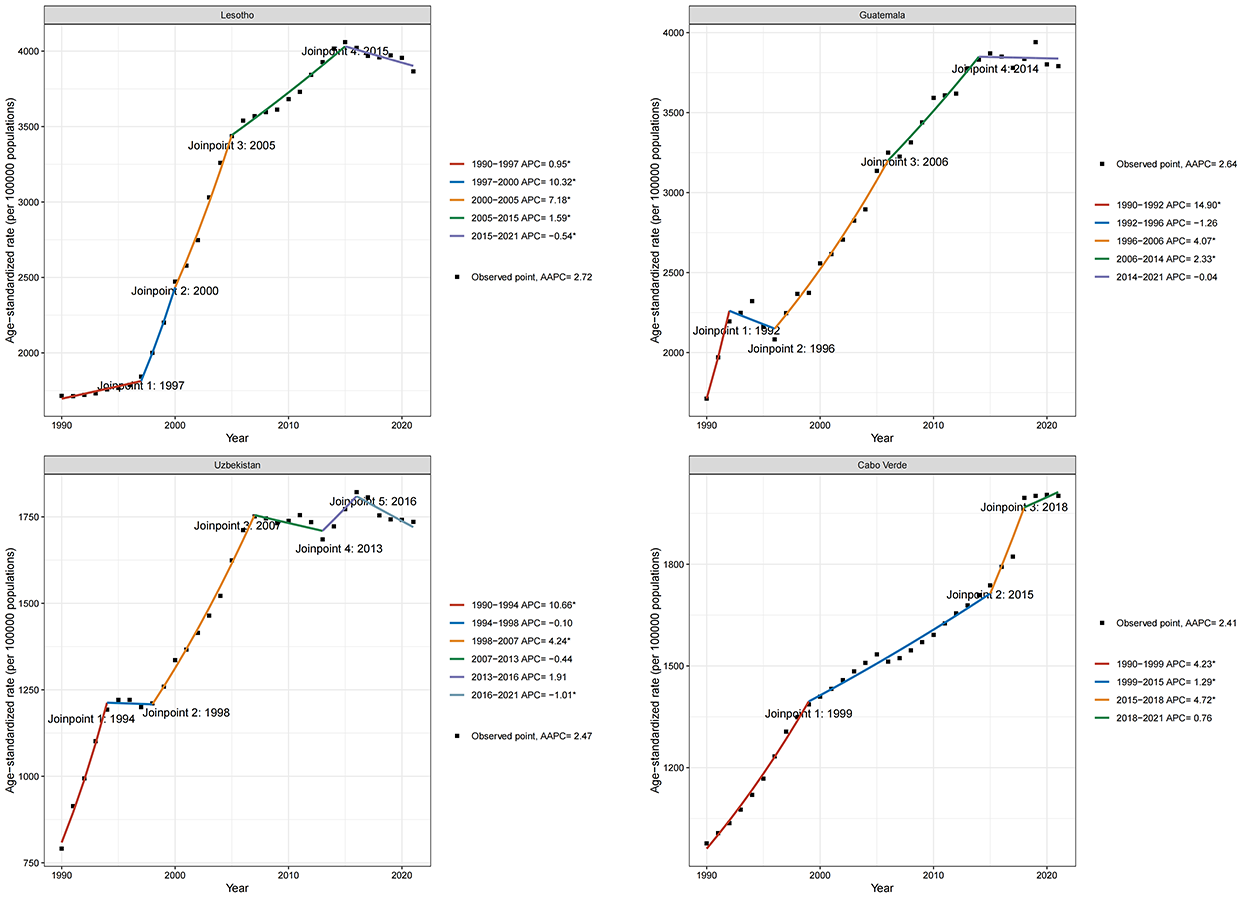
**

**Figure S8. The joinpoint regression analysis on the top four countries with the highest ASR of AAPC of mortality rate. ASR: age-standardized rate; AAPC: average annual percentage change.**

**
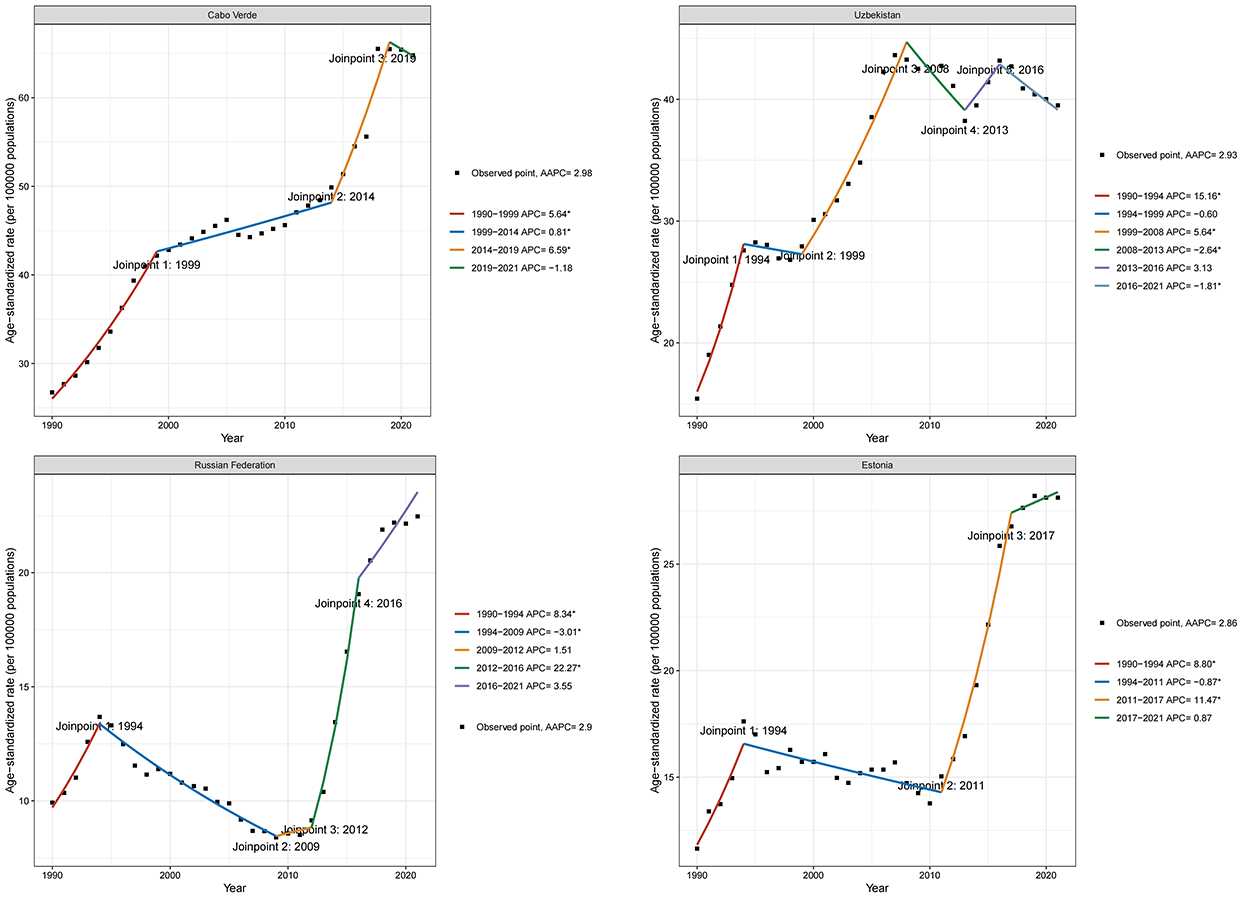
**

**Figure S9. Changes prevalence of diabetes and kidney diseases according to population, aging and epidemiologic change from 1990 to 2021 stratified by sexes (A: male; B: female); Changes incidence of diabetes and kidney diseases according to population, aging and epidemiologic change from 1990 to 2021 stratified by sexes (C: male; D: female); Changes DALYs of diabetes and kidney diseases according to population, aging and epidemiologic change from 1990 to 2021 stratified by sexes (E: male; F: female); Changes mortality of diabetes and kidney diseases according to population, aging and epidemiologic change from 1990 to 2021 stratified by sexes (G: male; H: female). DALYs: disability-adjusted life years.**

**
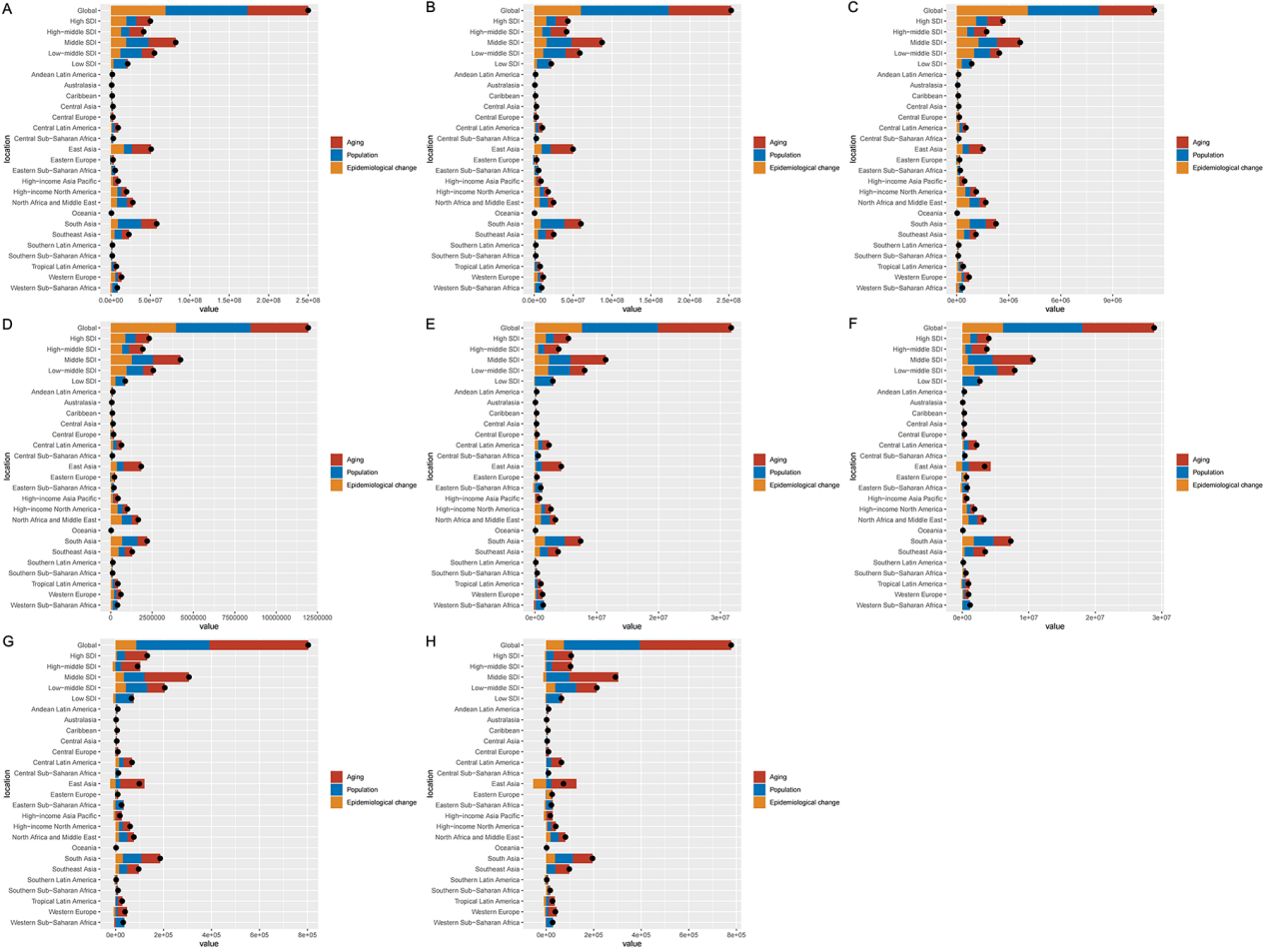
**
